# Adsorption and Oxidation of CO on Ceria Nanoparticles Exposing Single-Atom Pd and Ag: A DFT Modelling

**DOI:** 10.3390/ma14226888

**Published:** 2021-11-15

**Authors:** Vladimir A. Nasluzov, Elena A. Ivanova-Shor, Aleksey M. Shor, Svetlana S. Laletina, Konstantin M. Neyman

**Affiliations:** 1 Institute of Chemistry and Chemical Technology SB RAS, Federal Research Center “Krasnoyarsk Science Center SB RAS”, 660036 Krasnoyarsk, Russia; v.nasluzov@yandex.ru (V.A.N.); am.shor@yandex.ru (A.M.S.); shkulepo@rambler.ru (S.S.L.); 2 Departament de Ciència de Materials i Química Física and Institut de Quimica Teòrica i Computacional, Universitat de Barcelona, 08028 Barcelona, Spain; konstantin.neyman@icrea.cat; 3 ICREA (Institució Catalana de Recerca i Estudis Avançats), 08010 Barcelona, Spain

**Keywords:** CeO_2_-based nanomaterials, density functional calculations, single-atom catalysts, structure, reactivity

## Abstract

Various CO_x_ species formed upon the adsorption and oxidation of CO on palladium and silver single atoms supported on a model ceria nanoparticle (NP) have been studied using density functional calculations. For both metals M, the ceria-supported MCO_x_ moieties are found to be stabilised in the order MCO < MCO_2_ < MCO_3_, similar to the trend for CO_x_ species adsorbed on M-free ceria NP. Nevertheless, the characteristics of the palladium and silver intermediates are different. Very weak CO adsorption and the small exothermicity of the CO to CO_2_ transformation are found for O_4_Pd site of the Pd/Ce_21_O_42_ model featuring a square-planar coordination of the Pd^2+^ cation. The removal of one O atom and formation of the O_3_Pd site resulted in a notable strengthening of CO adsorption and increased the exothermicity of the CO to CO_2_ reaction. For the analogous ceria models with atomic Ag instead of atomic Pd, these two energies became twice as small in magnitude and basically independent of the presence of an O vacancy near the Ag atom. CO_2_-species are strongly bound in palladium carboxylate complexes, whereas the CO_2_ molecule easily desorbs from oxide-supported AgCO_2_ moieties. Opposite to metal-free ceria particle, the formation of neither PdCO_3_ nor AgCO_3_ carbonate intermediates before CO_2_ desorption is predicted. Overall, CO oxidation is concluded to be more favourable at Ag centres atomically dispersed on ceria nanostructures than at the corresponding Pd centres. Calculated vibrational fingerprints of surface CO_x_ moieties allow us to distinguish between CO adsorption on bare ceria NP (blue frequency shifts) and ceria-supported metal atoms (red frequency shifts). However, discrimination between the CO_2_ and CO_3_^2−^ species anchored to M-containing and bare ceria particles based solely on vibrational spectroscopy seems problematic. This computational modelling study provides guidance for the knowledge-driven design of more efficient ceria-based single-atom catalysts for the environmentally important CO oxidation reaction.

## 1. Introduction

Ceria, as a component of catalysts containing transition metals (M) Pd or Ag, is used in numerous applications ranging from the abatement of soot, volatile organic compounds, and CO [1,2,3,4,5,6,7,8,9,10] to the production of syngas [11] and CO_2_ activation [12,13]. As an active reducible support, ceria facilitates the dispersion of metals and MO_x_ phases on the surface [7,14,15,16,17] and provides lattice O atoms to oxidise reactants [2,9,17,18,19,20]. For instance, the interactions within Pd–ceria interfaces allow the synergistic oxidation/reduction of both subsystems [21], promote the oxidation of CO by lattice O atoms, and the oxidation of the reduced ceria by O atoms of CO_2_ [22]. Supported transition metals can also enhance the redox performance and oxygen storage capacity of ceria [23]. Often, high catalytic efficiency is achieved using a nanostructured ceria support via enhanced metal–support interaction, which improves the dispersion of metal particles and suppresses their sintering at elevated temperatures [3,4,7,8,9,11,19,24,25,26,27].

The M-containing surface phases of the aforementioned systems are represented by M_m_ [6,7,16,24,28,29] and MO_x_ [6,25,26,30] nanoparticles (NPs), charged metal clusters [7,24,31], solid M_x_Ce_1−x_O_2−δ_ solutions [25,26,27,32,33,34,35], and dispersed M_1_ or O_x_M_1_ ad-species [3,6,7,8,9,16,36,37]. Analysis of the crystalline environment of the Pd_1_ ad-species revealed that each Pd^2+^ ion in Pd/CeO_2_ catalysts prepared by the solution combustion method is coordinated, on average, by three O atoms [34]. This coordination mode of Pd_1_ is a feature of adsorption complexes with CO such as O_2_Pd_1_-CO/CeO_2_(111) [8], O_1_Pd_1_-CO/CeO_2_(111) [8], and O_1_Pd_1_-CO/CeO_2_(100) [9], while a Pd_1_-CO/CeO_2_(100) complex exhibits an O-Pd-O bridge [9]. In many cases, Pd_1_ is in a square-planar environment. Pd_1_ centres in Ce_1−x_Pd_x_O_2−δ_ crystals (x ≤ 0.15) reside on O_4_ units adjacent to Ce centres [32]. The doping of ceria with Pd results in a structure with the dopant ion displaced from the initial cationic position to the centre of the O_4_ unit [23]. Furthermore, Pd_1_ species are attached to the O_4_ unit of the Pd_x_Ce_1−x_O_2−x−δ_ lattice incorporating products of water dissociation [27]. Replacing every second upper-layer Ce^4+^ cation and one adjacent to it O^2−^ anion on the CeO_2_(110) surface with Pd^2+^ leads to a complex reconstruction and a low-energy surface geometry, with the dopant ion residing close to the centre of the square-planar O_4_ site [6]. Stable structures with square-planar O_4_Pd are also communicated on Pd-doped CeO_2_(111) [33] and edges of ceria NPs at intersecting {111} and {100} nanofacets [35]. Four-fold coordinated Pd adatoms are identified in the most stable O_4_Pd structures on the CeO_2_(110) surface [20] and {100} nanofacets [37]. The surface O_4_ sites are also capable of suppressing the sintering of Ag_1_ species, despite the fact that the Ag atom binds to the {100}-O_4_ pocket more weakly than other Group VIII–XI metal atoms [37,38]. The aforementioned thermally stable structures are relevant to the development of the single-atom catalysts [36,37,39].

To understand how the role of the ceria support varies in specific catalytic processes, it is crucial to examine the interactions of the involved reactants with various active centres of CeO_2_. For the ceria-supported metal catalysts of CO oxidation or CO_2_ activation, of primary interest are the interactions of O_2_, CO, and CO_2_ molecules with the metal–support interfaces [2,8,9,18,20,33,40,41,42,43]. Thus, modelling based on density functional theory (DFT) has recently addressed a variety of sites with M_1_-O_2_, M_1_-CO, and M_1_-CO_2_ entities on ceria [8,9,18,20,40]. The adsorption of CO on a single Pd atom embedded in the defect-free CeO_2_(111) surface and that containing O vacancies followed by NO reduction with CO was explored [18,40]. Surface complexes of CO and CO_2_ taking part in the catalytic cycle of CO oxidation on Pd_1_/CeO_2_(110), including Pd_1_-CO, Pd_1_-CO_2_, O_1_Pd_1_-CO species on the stoichiometric CeO_2_(110) surface and Pd_1_-CO_2_, Pd_1_-O_2_, O_2_-Pd_1_-CO ones on the O-deficient CeO_2_(110) surface, were calculated [20]. The CO oxidation routes passing via O_1_Pd_1_-CO, Pd_1_-CO, Pd_1_-CO_2_, O_2_Pd_1_-CO, and O_1_Pd_1_-CO_2_ moieties on the defect-free CeO_2_(111) surface [8] as well as on regular and O-deficient CeO_2_(100) surfaces [9] were also quantified. The catalytic CO oxidation according to the Mars-van-Krevelen mechanism combines the elementary steps of oxygen donation from a surface active centre to adsorbed CO and the subsequent replenishment of the support by stream oxygen; the much slower conversions of the first step are found to be rate-determining [8,9,19,20]. In addition to the M_1_-CO_2_ structures, surface carbonate complexes can be formed on M_1_–ceria interfaces before the desorption of CO_2_. To this end, the formation of tridentate Pd_1_-CO_3_ carbonates upon CO_2_ adsorption at the interface of Pd_1_ and O-deficient CeO_2_(111) surface was simulated [13] and the CO vibration frequencies of various ceria-supported Pd-CO species were calculated [8,9]. Unlike the quite extensive computational studies of Pd_1_CO_x_–ceria systems outlined above, no simulations of analogous Ag_1_CO_x_–ceria systems have been communicated so far to the best of our knowledge.

Previous studies have developed structural models of low-energy CeO_2_ NPs [44,45,46,47] and established that their {100} nanofacets notably stabilise single d-metal atoms [38,41,44,48,49,50,51]. In this work, we consider monoatomic Pd and Ag species located on a Ce_21_O_42_ NP [49] as models appropriately describing surface composites formed by single-atom Pd and Ag with nanostructured ceria. These two metals, which are neighbouring in the Periodic Table, interact very differently with ceria and behave as M-based species involved in CO oxidation. The quantification and in-depth understanding of such differences are still missing in the literature.

This study aims to (i) determine the structures of the lowest-energy complexes with CO, CO_2_, and CO_3_^2−^ moieties resulting from the interaction of CO with Pd and Ag single atoms anchored to the O_4_-pocket sites of the stoichiometric and O-deficient ceria NPs, (ii) analyse the structure and properties of these nanostructured adsorption systems versus earlier investigated analogues formed on extended ceria surface containing M_1_ centres, (iii) evaluate and rationalise the reactivity differences of Pd_1_/NP{100} and Ag_1_/NP{100} sites as active centres for CO oxidation (including the effect of M-atom on the formation of CO_3_^2−^ prior to CO_2_ desorption), and (iv) examine the vibrational fingerprints of the CO_x_ units accompanying the formation of various surface species. Obtained results related to all these aspects are summarised in the Conclusions section.

## 2. Models and Details of Calculations

**Figure 1:**
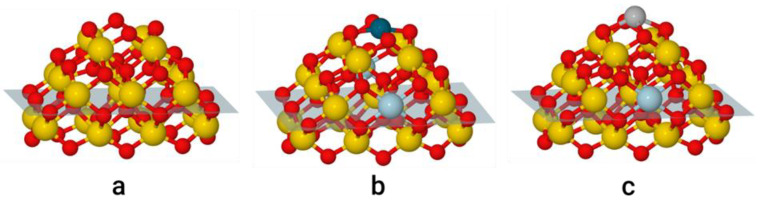
Models of Ce_21_O_42_ NP: (**a**) without metal atom; (**b**) with Pd atom anchored at {100} facet; (**c**) with Ag atom anchored at {100} facet. The Ce^4+^, Ce^3+^, Pd, Ag, and O atoms are shown in yellow, light blue, dark cyan, grey, and red, respectively. The atoms under the transparent plane are omitted for clarity in the Figure 2, Figure 3 and Figure 4.

Surface sites of the CeO_2_ substrate were represented by a putative global-minimum structure of stoichiometric NP Ce_21_O_42_ [46,47] exposing four O atoms on its top {100} nanofacet (a so-called O_4_-pocket [49]); see Figure 1a. M_1_/Ce_21_O_42_ models were created via anchoring a single M_1_ atom (M = Pd or Ag) to the O_4_-site of the NP; see Figure 1b,c. The removal of one O atom from the O_4_-pocket results in an O-deficient M_1_/Ce_21_O_41_ model with one O vacancy in ceria (not shown in Figures; see Supplementary Material for xyz-structures). CO adsorption does not change the number of O vacancies, whereas the oxidation of CO to CO_2_ and further transformation to CO_3_^2−^ require the expulsion of one or two O atoms from ceria, generating O vacancies. In the following, the number and origin of O vacancies in the NP Ce_21_O_42_ are labelled as NP[*n*/*l*], where *n* is a number of O vacancies present prior to CO adsorption (*n* = 0, 1) and *l* is a number of O vacancies created by the transfer of O atoms from ceria to the adsorbed CO to form CO_2_ or CO_3_^2−^ moieties (*l* = 0, 1, 2). For instance, the stoichiometric and O-deficient NP models M_1_/Ce_21_O_42_ and M_1_/Ce_21_O_41_ are referred to as M/NP[0/0] and M/NP[1/0], respectively.

The Vienna ab initio simulation package (VASP) [52,53] was employed to determine equilibrium structures of various isomers of the MCO_x_/NP[*n*/*x* − 1] complexes formed by the interaction of a CO molecule with Pd/NP[*n*/0] and Ag/NP[*n*/0] sites and transition state structures connecting selected equilibrium structures. The plane-wave basis with a 415 eV cutoff for the kinetic energy was used along with the projector-augmented wave description of the interactions of valence electrons (2s^2^2p^4^ for O, 4s^1^4d^9^ for Pd, 5s^1^4d^10^ for Ag and 5s^2^5p^6^6s^2^5d^1^4f^1^ for Ce) with the atomic cores [54,55]. The NP models were separated by a vacuum space of ~1 nm in the three Cartesian directions (typical cell dimensions 2 × 2 × 2 nm^3^) sufficient to eliminate the interaction between periodically repeated NP images [56,57]. All calculations were performed at the Γ-point of the reciprocal space. A generalised-gradient corrected (GGA) exchange-correlated functional PW91 [58] was utilised with the Hubbard-type on-site corrections U [59,60] for Ce4f states providing an improved description of Ce^3+^ ion formation in redox transitions [61,62]. The value of U = 4 eV (PW91 + U = 4 setup) were used in line with previous studies [45,48,61,63,64], though the usage of even such small U values may overestimate the formation energy of carbonates [62,65]. The minimum energy reaction paths MCO/NP[*n*/0] → MCO_2_/NP[*n*/1] → MCO_3_/NP[*n*/2] were represented with the points of the string method, and the transition states were approximated with polynomial splines [66,67].

Stabilities of the studied systems were quantified based on their formation energies *E^f^*:

(1) $$E^{f}=E\left(MCO_{x}/NP\left[0/x-1\right]\right)-E\left(CO\right)-E\left(M\right)-E\left(NP\left[0/0\right]\right)$$

for the MCO_x_/NP models obtained from defect-free MCO/Ce_21_O_42_ structure and

(2) $$E^{f}=E\left(MCO_{x}/NP\left[1/x-1\right]\right)-E\left(CO\right)-E\left(M\right)-E\left(NP\left[0/0\right]\right)+0.5\times E\left(O_{2}\right)$$

for the MCO_x_/NP models obtained from O-deficient MCO/Ce_21_O_41_ structure, where *E*(*O*_2_) is total energy of a free O_2_ molecule. The binding energies *E_b_* of CO and CO_2_ molecules in the models under scrutiny were calculated as follows:

(3) $$E_{b}\left(CO\right)=E\left(MCO_{x}/NP\left[n/x-1\right]\right)-E\left(CO\right)-E\left(M/NP\left[n/0\right]\right),\;x=1–3;\;n=0,\;1$$

(4) $$E_{b}\left(CO_{2}\right)=E\left(MCO_{x}/NP\left[n/x-1\right]\right)-E\left(CO_{2}\right)-E\left(M/NP\left[n/1\right]\right),\;x=2,\;3;\;n=0,\;1$$

The energies (3) and (4) of the same MCO_2_/NP complex were used to estimate the energy of the overall oxidation process CO(gas) + M/NP[*n*/0] → CO_2_(gas) + M/NP[*n*/1]:

(5) $$E_{ox}=E_{b}\left(CO\right)\left(MCO_{2}/NP\left[n/1\right]\right)-E_{b}\left(CO_{2}\right)\left(MCO_{2}/NP\left[n/1\right]\right)$$

or

(6) $$E_{ox}=E_{b}\left(CO\right)\left(MCO/NP\left[n/0\right]\right)+E_{CO2}^{*}-E_{b}\left(CO_{2}\right)\left(MCO_{2}/NP\left[n/1\right]\right),\;n=0,\;1$$

where E*_CO2_ is the energy of CO to CO_2_ oxidation at a metal site; i.e., the heat of MCO/NP[*n*/0] → MCO_2_/NP[*n*/1] transformation:

(7) $$E_{CO2}^{*}=E\left(MCO_{2}/NP\left[n/1\right]\right)-E\left(MCO/NP\left[n/0\right]\right)$$

where, the asterisk indicates that CO and CO_2_ molecules are adsorbed.

Harmonic vibrational frequencies of CO_x_ groups were calculated by diagonalising the mass-weighted Hessian matrix constructed of differences of the first derivatives of total energy, obtained by displacements by ±0.015 Å in all Cartesian directions of the M, C, and O_x_ atoms as well as neighbouring ceria atoms within 3.6 Å around them.

## 3. Results and Discussion

**Figure 2:**
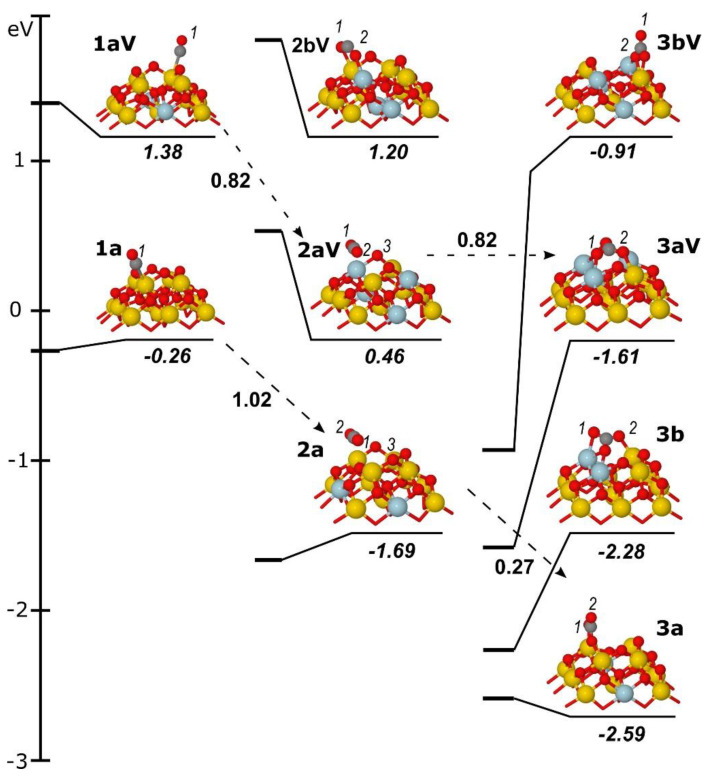
Structures and formation energies *E^f^* (in eV) of the complexes created by the interaction of CO with the Ce_21_O_42−*n*_ sites (*n* = 0, 1): CO/Ce_21_O_42_ (**1a**), CO/Ce_21_O_41_ (**1aV**), CO_2_/Ce_21_O_41_ (**2a**), CO_2_/Ce_21_O_40_ (**2aV, 2bV**), CO_3_/Ce_21_O_40_ (**3a, 3b**) and CO_3_/Ce_21_O_39_ (**3aV, 3bV**). Activation energies (*E^≠^*) of selected transition states are shown near the dashed arrows connecting corresponding initial reagent and product. The C atoms are shown in dark grey. O atoms numbered with “1”, “2”, and “3” enter CO, CO_2_, and CO_3_ moieties, respectively. For further explanations, refer to the main text and the Figure 1 caption.

**Figure 3:**
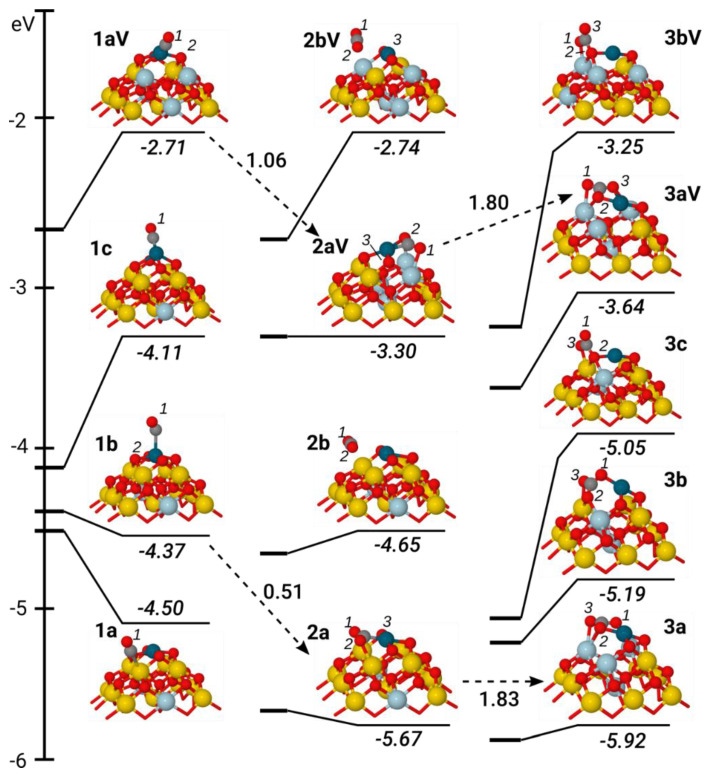
Structures and formation energies *E^f^* (in eV) of the complexes created by the interaction of CO with the PdCe_21_O_42−*n*_ sites (*n* = 0, 1): PdCO/Ce_21_O_42_ (**Pd1a, Pd1b, Pd1c**), PdCO/Ce_21_O_41_ (**Pd1aV**), PdCO_2_/Ce_21_O_41_ (**Pd2a, Pd2b**), PdCO_2_/Ce_21_O_40_ (**Pd2aV, Pd2bV**), PdCO_3_/Ce_21_O_40_ (**Pd3a, Pd3b, Pd3c**) and PdCO_3_/Ce_21_O_39_ (**Pd3aV, Pd3bV**). Activation energies (*E^≠^*) of selected transition states are shown near the dashed arrows connecting corresponding initial reagent and product. For further explanations, refer to the main text and the captions to Figure 1 and Figure 2.

**Figure 4:**
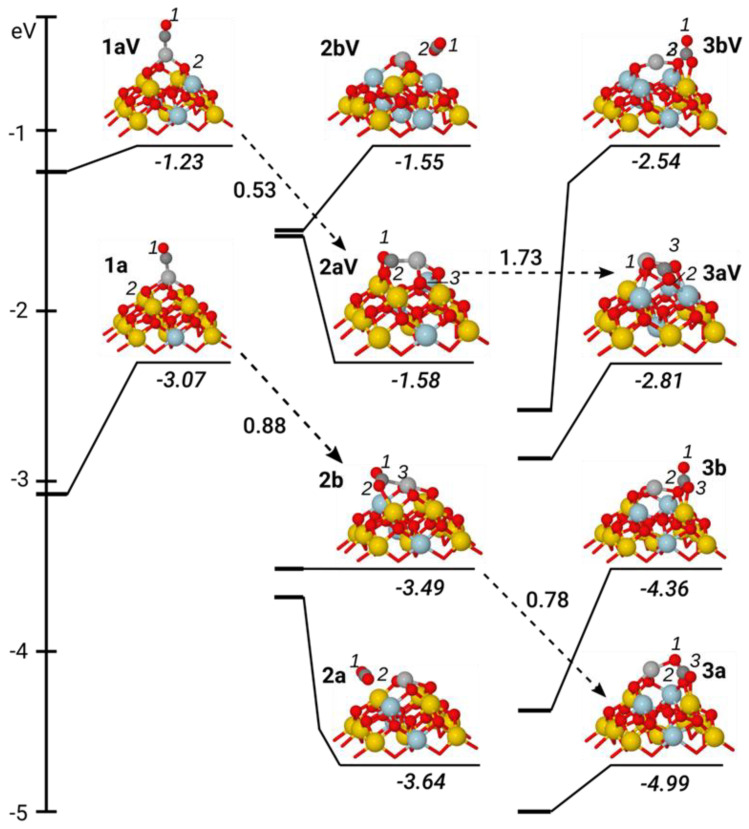
Structures and formation energies *E^f^* (in eV) of the complexes created by the interaction of CO with the Ag/Ce_21_O_42−*n*_ sites (*n* = 0, 1): AgCO/Ce_21_O_42_ (**Ag1a**), AgCO/Ce_21_O_41_ (**Ag1aV**), AgCO_2_/Ce_21_O_41_ (**Ag2a, Ag2b**), AgCO_2_/Ce_21_O_40_ (**Ag2aV, 2bV**), AgCO_3_/Ce_21_O_40_ (**Ag3a, Ag3b**) and AgCO_3_/Ce_21_O_39_ (**Ag3aV, Ag3bV**). Activation energies (*E^≠^*) of selected transition states are shown near the dashed arrows connecting corresponding initial reagent and product. For further explanations, refer to the main text and the captions to Figure 1 and Figure 2.

Lowest-energy geometries and formation energies *E^f^* of metal-free CO_x_/NP[*n*/*x* − 1] and metal-containing MCO_x_/NP[*n*/*x* − 1] complexes are shown in Figure 2, Figure 3 and Figure 4 (for xyz-structures see Supplementary Material). Table 1 displays the parameters used to specify attachment modes (coordination) of the CO_x_ groups. Along with the notations MCO_x_/NP[*n*/*x* − 1], shorter ones **MxL** and **MxLV** were used for complexes with *n* = 0 and *n* = 1, respectively, where **L** is a sequential identifier of the relative energy of a given isomer among isomers with the same **M**, **x**, and **n** (**a**—the most stable, **b**—the second most stable, **c**—the third most stable). Ce ions with magnetic moments close to 1, at a variance to 0 for most of the cations, were qualified as Ce^3+^ ions resulting from the reduction of Ce^4+^ by electrons of MCO_x_ moieties or O vacancies.

To specify the coordination modes of CO_2_ in MCO_2_/NP[*n*/1] and of CO_3_^2−^ in MCO_3_/NP[*n*/2] complexes, additional three-digit indices were used [68] resulting in the notation that being invoked only when discussing the coordination modes of CO_x_ species. For carbonate complexes, a notation abc (integer a, b and c range from 0 to 3) determines numbers of substrate atoms, to which each O atom in CO_3_^2−^ is coordinated. The middle digit b corresponds to the O atom with the highest coordination. A dot in indices a.bc or ab.c specifies that two O atoms of CO_3_^2−^ (corresponding to a and b or b and c, respectively) form a bidentate bond with one atom of the substrate. Dotless abc identifiers designate CO_3_^2−^ groups with each O atom coordinated to a different substrate atom. When the three-digits notation is used to specify coordination of CO_2_ in MCO_2_/NP[*n*/1] complexes, the first digit a gives the number of substrate atoms coordinated to C atom. For instance, in Figure 3, each of the atoms of CO_2_ in structure 111-**Pd2a** contacts different ions of the Pd/Ce_21_O_41_ subsystem, and in complex 1.21-**Pd2aV**, one of the O atoms of CO_2_ attaches to Pd and Ce atoms of Pd/Ce_21_O_40_, and C atom also to Pd.

### 3.1. Structure, Charge State, and Relative Energies of Surface Complexes with CO_x_

#### 3.1.1. Adsorption Sites

The particle Ce_21_O_42_ exposes a {100} nanofacet (Figure 1a), which can bind metal atoms much stronger than its {111} nanofacets do [38]. We anchored single Pd and Ag atoms to the {100} nanofacet composed of four nearly coplanar two-coordinated oxygen centres forming an O_4_-pocket with diagonals of 459 and 443 pm. This arrangement is appropriate for accommodating transition metal cations with typical M-O bond lengths of 185–210 pm [37]. The distances between the O_4_-pocket atoms and the neighboring Ce atoms, 214–215 pm, are shortened versus the Ce-O distances of three-coordinated surface O atoms and four-coordinated inner O atoms, 230–250 pm.

The Ag atom binds by 2.27 eV to the O_4_-site (Figure 1b), making the two diagonals of the latter almost equal. The two types of Ag-O bond lengths, 236 and 242 pm, agree with Ag-O distances of 239 and 241 pm calculated at the same theory level on a larger Ce_40_O_80_ NP [37]. Furthermore, the present location of the Ag atom 96 pm above the O_4_-plane is close to the elevation by 90 pm on Ce_40_O_80_ [37]. A slightly larger above-plane elevation, by 102 pm, was calculated for a two-fold coordinated Ag single atom adsorbed on the Fe_3_O_4_(001) surface with energy 2.75 eV [69]. Ag atom adsorption moves O_4_ centres upwards, elongating the involved Ce-O distances to 218–223 pm. One Ce ion reduced to +3 state, pointing to the Ag^+^ oxidation state.

The Pd adatom is located nearly in the O_4_ plane (Figure 1c), with an out-of-plane displacement of 18 pm, adopting a favourable planar coordination. All four Pd-O bonds are equal to 205 pm—exactly the same as for the Pd/Ce_40_O_80_ model [37]. The formation of PdO_4_ species moves O atoms closer to Pd, contracting diagonals of the O_4_ square to 408 pm and elongating the corresponding Ce-O distances to 230 pm—the value typical for three-coordinated O centres. The high adsorption energy of the Pd atom, 4.24 eV, further increases upon interactions with the O_4_-site of larger ceria NPs [37]. Two Ce^3+^ ions appeared upon Pd adsorption, indicating the oxidation state Pd^2+^.

An O-deficient site is created by the removal of the weakest bonded O atom (with *E(O_V_)* = 1.87 eV, Table 2) from the O_4_-pocket of the Ce_21_O_42_ particle (Figure 1). Two Ce^3+^ centres resulting from the O removal in the second Ce layer cause the elongation of the Ce^3+^-O bonds by ~0.1 Å. O vacancy formation in the Ag/Ce_21_O_42_ model requires 2.08 eV (Table 2), nearly the same amount as for the metal-free O_4_-site. Thus, an extra energy cost due to breaking the Ag-O bond (in addition to two Ce-O bonds of Ce_21_O_42_) is estimated at ~0.2 eV. Two Ag-O bonds are contracted to 2.15 Å upon O removal, whereas the third bond is elongated from 2.36 to 2.49 Å. A similar bonding situation has been found in our previous work, where an Ag single atom was anchored to the bottom {100} nanofacet of Ce_21_O_42_ NP [38]. Alternatively, the creation of an O-deficient Pd/Ce_21_O_41_ site requires substantial energy costs of 3.08 eV (Table 2).

Evidently, an extra energy of ~1.2 eV is needed to cleave one Pd-O bond. Note that the Pd atom remains after O removal in a virtual square-planar environment with one coordination site empty and the lengths of the remaining three Pd-O bonds unchanged, 206–209 pm, vs. the PdO_4_ site of Pd/Ce_21_O_42_. No reduction of Ag^+^ and Pd^2+^ ions occurs upon O vacancy creation, since the leaving O atom donates two electrons to two Ce^4+^ ions, increasing the number of Ce^3+^ centres by two (to three for Ag/NP and to four for Pd/NP).

#### 3.1.2. Carbonyl Species

Let us first consider the adsorption of a CO molecule on a Ce ion of bare ceria particles (Figure 2). For both the pristine and O-deficient structures, CO adsorption energy is very small, at −0.26 and −0.23 eV, respectively (Table 1). No geometry changes are calculated upon bringing together the CO molecule and ceria species. In particular, the C-O bond is retained at 114 pm as in the free CO molecule. The C end of the CO adsorbate is 293 (**1a**) and 297 pm (**1aV**) away from the nearest Ce^4+^ ion of NP (Table 1). CO forms angles at 176° and 172° with the Ce site. Thus, CO is rather physisorbed than chemisorbed on the Ce site of the pristine and reduced forms of the metal-free NP. The same geometry and adsorption energy of −0.26 eV (Table 1) was calculated for the Pd-containing **Pd1a** structure with the CO molecule bound to a Ce^4+^ ion (Figure 3), indicating that the filling of the O_4_-pocket by the Pd atom mainly affects the local structure of the PdO_4_ site.

An even smaller CO adsorption energy, −0.13 eV, was calculated for the coordinatively saturated Pd centre of the PdO_4_ site of the unreduced model (**Pd1b**, Figure 3). Here, CO binds the Pd atom at an angle of 131° and rather long Pd–C contact of 241 pm.

CO adsorption causes minor distortions in the Pd/Ce_21_O_42_ structure: Pd-O bonds extend from 205 to 207 pm, and Pd moves by 27 pm above the O_4_ plane. The C-O bond elongates by just 1 pm vs. gas-phase CO. No CO adsorption was reported on Pd atoms in the PdO_4_ environment saturated by two O centres of the CeO_2_{100} surface and two O adatoms [9]. Furthermore, *E_b_(CO)* < −0.2 eV was calculated for coordinatively saturated Pd atoms in PdO(100). Thus, a very low *E_b_(CO)* for **Pd1b** is related to the saturation of the coordination sphere of the Pd atom in the O_4_-pocket of the {100} nanofacet by O atoms; the formation of an additional Pd-C bond competes with quite strong Pd-O bonds. The exceptional stability of Pd^2+^ ions in square-planar oxygen environment in CeO_2_ materials is also claimed in other experiments [6,27,32]. Note that CO adsorption on all Pd-containing models does not change the oxidation state Pd^2+^, except for **Pd1c** with an endothermic mode by 0.13 eV CO adsorption (Table 1), where a Ce^3+^ → Ce^4+^ transition indicated a reduction to Pd^+^.

In an O-deficient structure, **Pd1aV** (Figure 3), the adsorbed CO occupies one of four places around Pd. This results in Pd-C bond shortening to 187 pm (Table 1), as in Pd_1_-CO/CeO_2_(110) complexes bonded with two (Pd-C = 184 pm) and three O surface atoms (Pd-C = 188 pm) [20]. The Pd-C-O angle in **Pd1aV** is close to 180°. The elongation of the C-O distance from 114 to 116 pm indicates noticeable d → 2π* back-donation. In **Pd1aV**, CO binds at a vacant coordination site around Pd, no bonds are broken, and adsorption induced geometry changes are minor. As a result, PdCO/Ce_21_O_41_ is stabilised by 1.7 eV with respect to separated Pd/Ce_21_O_41_ and CO fragments (Table 1). Interestingly, similarly strong CO binding as that for **Pd1aV**, 1.77 eV, and a Pd-C distance of 186 pm were calculated for the two-fold coordinated single Pd atom in Pd_1_/Fe_3_O_4_(001) [69]. The CO bond to the isolated Pd atom is also similarly strong, at 1.8 eV [70]. The values of 1.6 eV [8] and 1.9 eV [9] were calculated for Pd_1_-CO/CeO_2_(111) and O_1_Pd_1_-CO/CeO_2_(100) complexes, respectively, with Pd_1_ coordinated to three O atoms including that of isolated O_1_Pd_1_ species. A CO adsorption energy of −1.5 eV was calculated for the Pd_1_-CO/CeO_2_(110) complex with the Pd^+^ ion between two three-fold O atoms [20]. In the series of complexes Pd_1_-CO/CeO_2_(111), O_1_Pd_1_-CO/CeO_2_(111), and O_2_Pd_1_-CO/CeO_2_(111), *E_b_(CO)* decreases (by module) with the growth of the Pd_1_ coordination number from 1.6 to 0.9 and to 0.6 eV [8]. 

The binding of CO to O-defect-free **Ag1a** and O-deficient **Ag1aV** complexes is moderately strong, at about 0.8 eV (Table 1), and essentially independent of the coordination—AgO_4_ or AgO_3_—of the Ag atom. This adsorption energy value fits the calculated values of −0.85 eV for CO adsorption at the two-fold coordinated Ag single atom in the Ag_1_/Fe_3_O_4_(001) site and −0.94 eV for the two-fold coordinated Ag atom at the AgO_2_(111) surface well [69]. Structures of the AgCO fragments in **Ag1a** and **Ag1aV** are very similar (Figure 4). The Ag-C distance of ~200 pm (Table 1) coincides with the value computed for the Ag_1_CO moiety at Fe_3_O_4_(001). The C-O bond, 115 pm, is 1 pm longer than that in the free CO molecule. The Ag-C-O angle of ~170° points to slight deviation from the typically favoured linear bonding geometry. In both structures, CO adsorption triggers a further displacement of Ag atom out-of-plane of neighbouring O atoms, reaching values of 132 and 152 pm for **Ag1a** and **Ag1aV**, respectively (vs. 96 and 60 pm for CO-free structures). Ag-O bond lengths vary substantially, ranging from 237 to 258 pm for the AgO_4_ unit and from 224 to 264 pm for the AgO_3_ unit. The notable distortions at the Ag/CeO_2_ interface are not reflected in CO adsorption energies. This finding is in line with the quite weak Ag-O bonding estimated at 0.2 eV (see Section 3.1.1).

In summary, the modification of the ceria NP with single Pd and Ag atoms strongly affects its affinity to CO. Effects of Pd and Ag atoms are different. Due to strong Pd-O bonds, the saturated PdO_4_ site is almost inactive towards CO adsorption, whereas the PdO_3_ unit with a vacant coordination place readily traps CO with a substantial energy gain. In contrast, AgO_4_ and AgO_3_ centres with weak Ag-O bonds and a more flexible geometry are more prone to adsorb CO molecules with equally moderate energies.

#### 3.1.3. Carbon Dioxide Species

For the metal-free ceria, only weakly adsorbed CO_2_ species were calculated: binding energies are −0.25 and −0.15 eV for **2a** and **2aV** models, respectively (Table 1). These energies are comparable with the values calculated for linearly-adsorbed CO_2_ at extended CeO_2_(100), (110), and (111) surfaces [71,72,73,74,75,76]. In both complexes, the linear geometry of CO_2_ as in the gas-phase molecule is preserved: C-O bond lengths are 118 pm and the O-C-O angle is 178°. The C atoms of CO_2_ molecules are located above the O vacancy parallel to the surface of NP (Figure 2); in case of **2a**, the O atom from the low-lying layer moves up and forms an O-C contact of 288 pm (vs. 390 pm in **2aV**).

The contacts of O atoms of the CO_2_ group with Ce atoms are ~300 pm in **2a** and 304–331 pm in **2aV** (Table 1). All attempts to locate more stable structures with a bent CO_2_ moiety have led to carbonate moieties (described in detail in Section 3.1.4). No minima corresponding to bent CO_2_ structures of carboxylate type were found. The only located structure with distorted CO_2_ (**2bV** at Figure 2) was found to be 0.75 eV less stable relative to the linear **2aV** model. It features C-O bond lengths of 125 pm and an O-C-O angle of 131°. The O atoms of CO_2_ are at 252 and 257 pm from Ce atoms. The positive CO_2_ binding energy in **2bV** of 0.6 eV (Table 1) indicates exothermic CO_2_ release. Since only one Ce^3+^ ion is present after CO to CO_2_ transformation, CO_2_ in **2bV** is negatively charged, forming a CO_2_^−^ anion. Thus, CO_2_ binds weakly and easily desorbs from bare Ce_21_O_42−x_ NP.

The metal-containing Pd/NP and Ag/NP models also feature structures with “linear” and “bent” CO_2_ (Figure 3 and Figure 4). Similar to the bare ceria, linear CO_2_ is weakly bound in **Pd2b**, **Pd2bV**, **Ag2a,** and **Ag2bV**, by less than 0.2 eV (Table 1). All distances between atoms of CO_2_ molecule and ceria are longer than 300 pm. In contrast, in “bent” CO_2_ structures **Pd2a**, **Pd2aV**, **Ag2b,** and **Ag2aV,** CO_2_ approaches more closely to the NP surface and forms C-M and O-Ce bonds at 193–234 and 250–270 pm, respectively. Interestingly, despite the sizable changes at the metal–adsorbate interface when going from “linear” to “bent” structures, the pairs **Ag2aV**/**Ag2bV** and **Ag2a**/**Ag2b** are isoenergetic within 0.15 eV. This observation supports our earlier finding that the formation of a new Ag-ligand bond (e.g., Ag-C with CO_2_) does not require substantial energy (limited to 0.2 eV). The bonds of CO_2_ in **Ag2b** are symmetrically stretched to 121 pm and the O-C-O angle is reduced to 145°, thus indicating the formation of a weakly-bound carboxylate-like complex. CO_2_ is coordinated to Ag via the C atom at a distance of 234 pm and forms an (O)C-Ag-O angle of 175° with the Ag-O bond in trans-position. The Ag atom in AgCO_2_/Ce_21_O_41_ rises above the O_3_-plane by 52 pm (vs. 132 pm in AgCO/Ce_21_O_42_), similar to O-deficient adsorbate-free Ag/Ce_21_O_41_ complex (60 pm). Thus, the surrounding of the Ag center in **Ag2b** is nearly square-planar. The formation of the CO_2_ moiety in **Ag2aV** does not lead to the entire detachment of the surface O* atom by adsorbed CO (Figure 4). Rather, the **Ag2aV** model can be viewed as a complex with CO inserted into the O*-Ag bond; as a result, the O*-Ag distance is stretched from 227 to 287 pm. Upon this insertion, Ce-O* bonds of the Ce-O*-Ce bridge are elongated from 218–233 pm in **Ag1aV** to 249–257 pm in **Ag2aV** (Table 1). The shortest Ce-O* distance in **Ag2b** is 268 pm. The CO_2_ fragment in **Ag2aV** is more strongly distorted than in **Ag2b**: C-O bonds are 130 pm and the O-C-O angle is quite small, at 114°. The Ag-CO_2_ distance (207 pm) is typical for metal–CO_2_ complexes with an η^1^-C type of CO_2_ coordination [77]. Energies of **Ag2b** and **Ag2aV** models differ by 1.9 eV, the value close to the O vacancy formation energy at the O_4_-pocket (Table 2).

The PdCO_2_-containing complexes, **Pd2a** and **Pd2aV**, with a “bent” CO_2_ moiety (Figure 3) are characterised by substantial CO_2_ binding energies of −1.2 and −0.7 eV, respectively (Table 1). Both models exhibit a carboxylate-like structure of CO_2_: C-O bonds are 124–130 pm long and the O-C-O angle is 130 ± 3°. In the **Pd2a** structure, CO_2_ is η^1^-coordinated to the Pd atom with the Pd-C distance, 206 pm, comparable to three Pd-O bonds around the metal center, 208–220 pm. In the **Pd2aV** complex, the CO_2_ molecule is η^2^-coordinated to Pd via the short Pd-C bond, 193 pm, and long Pd-O contact of 237 pm; two other Pd-O bond lengths are 205–220 pm. The Pd atom in both **Pd2a** and **Pd2aV** complexes is nearly in a square-planar environment with Pd shifting from the ligand plane by 25–30 pm. Interestingly, no ceria-supported PdO_3_ structures with calculated CO_2_ binding energies more than −0.4 eV (by module) were found in the literature [8,9,20]. The larger values −0.82 eV [20] and −0.96 eV [8] were calculated for PdO_2_ units at CeO_2_(110) and CeO_2_(111) surfaces. In part, this can be explained by a favourable square-planar geometry within the O_3_Pd-CO_2_ fragment formed at the {100} nanofacet of Ce_21_O_42_ NP. Indeed, such structures are hindered at extended CeO_2_ surfaces by geometrical constrains.

Thus, similar to the CO case, the strongest bonding is calculated for the PdO_3_ site with one vacant coordination place. This is followed by the PdO_2_ unit with two vacant valences, which binds CO_2_ in a side-on fashion. Both structures are characterised by CO_2_ bending. The adsorption of CO_2_ in linear mode results in an energy gain less than 0.2 eV for both Pd and Ag derivatives. In contrast to the Pd derivatives, both ”linear” and “bent” modes have similar small adsorption energies for the Ag systems. Thus, AgO_3_ and AgO_2_ sites again do not show differences in adsorption properties, whereas their Pd analogs do.

#### 3.1.4. Carbonate Species

Carbonate species are formed upon the coordination of the CO_2_ molecule via the C atom to an O center of ceria NP. Thus, CO_3_^2−^ formation can be considered as a form of CO_2_ adsorption, and CO_2_ binding energy can be applied to the estimation of the stability of these carbonate species.

The carbonate-like CO_2_ adsorption at bare ceria NPs with binding energies of 0.84–2.22 eV (Table 1) is the most exothermic of all types of CO_2_ coordination discussed above. The strongest binding of 2.22 eV is calculated for the tridentate 2.2.2-structure (**3aV**, Figure 2), with each O atom of the CO_3_ moiety coordinated to two Ce ions. The flat-lying carbonate fragment of **3aV** fills two O vacancies with its oxygen atoms; i.e., all O atoms of the CO_3_ moiety are in neighbouring O-positions of the O_4_-pocket. The E_b_(CO_2_) energy for **3aV** is between –1.9 and –2.3 eV, respectively, calculated for its formal 2.2.2-CO_3_ analog on regular CeO_2_(100) [78] and bidentate 220-CO_3_ on O-deficient CeO_2_(110) [74]. The 2.2.2-model features three C-O bonds equally stretched to 130 pm and O-C-O angles of 118 ± 2°. Three of four Ce^3+^ ions are located in the Ce_4_ square just below the O_4_-pocket of the {100} nanofacet. The other tridentate 1.21-structure **3b** is stabilised by only 0.84 eV relative to the sum CO_2_ + NP (Table 1). Here, each of the two O atoms of CO_2_ has only one contact with Ce ions of 238 and 247 pm. As in the **3aV** model, the CO_3_ fragment in **3b** has a slightly distorted C_3V_ symmetry, with C-O bonds of 130 ± 2 pm and O-C-O angles of 117–122°. Its CO_3_ plane is tilted by 40° relative to the Ce_4_ layer. The CO_2_ binding energy in the 1.21-carbonate is comparable to value −0.72 eV calculated for the flat-lying 2.21-CO_3_ structure [71] formed upon CO adsorption at the stoichiometric CeO_2_(100) surface with every second atom of the O layer removed. The deletion of an O atom from 1.21-**3b** to give 2.2.2-**3aV** costs only 0.67 eV. Thus, energy for the removal of a lattice O atom (~2.3 eV for formation of a 2nd O vacancy) is compensated by the formation of two new O-Ce contacts in **3aV** with ~1.6 eV energy gain. At variance, the energy difference between two 1.20-structures **3a** → **3bV**, with a bidentate coordination, 1.68 eV, does not decrease with respect to *E(O_v_)* for Ce_21_O_42_ → Ce_21_O_41_ transition, since both models have a similar bonding pattern of carbonate species. Thus, not surprisingly, the CO_2_ binding energies for **3a** and **3bV** models are not very different, at –1.15 and −1.52 eV, respectively (Table 1). In both **3a** and **3bV** models, the CO_3_ moiety is tied by three O-Ce bonds at 227, 238, and 245 pm. The three O-C bond lengths are different and increase from 122 to 133 and to 137 pm with the growing coordination number of O atom from 0 to 1 and to 2, respectively. Notably, at the O-deficient ceria, the flat-lying structure **3aV** is preferred over the standing one **3bV** by 0.7 eV. A similar difference of 0.84 eV was calculated for CO_3_ moieties oriented parallel and perpendicular to the O-defective CeO_2_ (100) facet [71]. The trend of destabilising carbonate species at ceria substrates upon surface enrichment by O atoms [71] is also supported by the present data.

Three types of carbonate structures—standing (perpendicular), tilted and flat-lying (parallel)—were also located for metal-containing ceria NPs (Figure 3 and Figure 4). The “standing”-type carbonate species is coordinated in a bidentate way in 1.30-models **Pd3c**, **Pd3bV**, **Ag3b**, and **Ag3bV**. These structures are very similar to 1.20-carbonates **3a** and **3bV** at bare ceria NPs, with the difference that the CO_3_ moiety is additionally bound with the M center via one short M-O bond of 211 pm (233 pm in **Ag3b**). This extra metal–oxygen bond induces the elongation of other three O-Ce contacts by 5–15 pm as well as elongates O⋯CO_2_ contact by 4–9 pm, which probably weakens CO_2_ binding from −1.15–1.52 eV in metal-free models to −0.86–1.13 and −0.39–0.50 eV in Ag- and Pd-carbonates, respectively (Table 1). Thus, for Pd-systems, carbonate-like CO_2_ adsorption in a “standing” mode is weaker than adsorption in carboxylate PdCO_2_ form. Notably, Pd is in a zero-oxidation state in both types of complexes.

Stronger CO_2_ bindings, of −1.44 and −1.40 eV, were calculated for the “flat-lying” structures 2.2.1-**Pd3a** and 2.2.2.-**Ag3aV**, respectively (Table 1). The CO_3_ fragment is bidentately coordinated with two M-O contacts: almost equal in **Pd3a** (207 and 211 pm) and asymmetric in **Ag3aV** (216 and 265 pm). In the **Pd3a** structure, all O atoms of the CO_3_ moiety leave their lattice positions to form short Pd-O bonds (Figure 3). This leads to the creation of a CO_3_^2−^ unit with a formal charge of −2 and oxidation of Pd to the +2 oxidation state. Even shorter M-O bonds are formed between metal centres and O atoms of the ceria support: 201–202 pm for Pd-O and 206 pm for Ag-O bonds. Thus, the Pd atom is four-coordinated whereas Ag is three-coordinated by O ligands. Importantly, the PdO_4_ unit in **Pd3a** is in a slightly distorted stable square-planar configuration. Likely, this contributes greatly to making **Pd3a** the most energetically favourable among all studied ceria-supported PdCO_x_ species. The **Pd3a** complex is even 0.25 eV more stable than another square-planar structure: carboxylate **Pd2a** complex with O_3_PdCO_2_ unit. The plane of the CO_3_ subsystem forms a small angle of about 20^°^ with the O_3_M plane. The “lying” **Ag3aV** structure is only 0.27 eV stabilised with respect to “standing” **Ag3bV,** whereas the analogous stabilisation for **Pd3a** → **Pd3c** transition reaches 0.87 eV.

The **Ag3a** 1.21-complex with the “tilted” coordination mode of CO_3_ is the most favourable Ag-carbonate structure with a CO_2_ adsorption energy of −1.49 eV (Table 1). Similar to the 1.30-model **Ag3b**, the CO_2_ moiety in the **Ag3a** structure is tied in an η-C,O fashion with ceria support by means of two bonds. While the distance O-C-O⋯Ce is the same in **Ag3a** and **Ag3b**, the O⋯CO_2_ contact in **Ag3a** is shortened to 135 pm from 141–146 pm in **Ag3b**. The shorter Ag-O contact of 221 pm (vs. 233 pm in **Ag3b**) is formed with the O atom, which has no bonds with CeO_2_; this is comparable to the Ag-O bond length of 218 pm with ceria. Remarkably, “tilted” 1.21-complex **Ag3a** is by 0.63 eV stabilised with respect to “standing” 1.30-model **Ag3b** with an *E(CO_2_)* of −0.86 eV and reaches a CO_2_ adsorption energy of −1.52 eV of the metal-free “standing” **3bV** model. 1.21-**Ag3a** complex has a nearly identical CO_2_ adsorption energy (within 0.1 eV) to the “lying” 2.2.2-**Ag3aV** model. In both complexes, the cationic Ag^+^ center is three-coordinated. Despite the closer contact of the CO_3_ unit with ceria support in the 2.2.2-model, its formation from the **Ag3a** structure by the removal of O bound to the Ag atom requires a substantial energy of 2.18 eV. In contrast, O deletion from 1.21-**Pd3b** to give 2.2.1-**Pd3aV** is endothermic by only 1.55 eV. Both “tilted” complexes, **Pd3b** and **Pd3aV**, have CO_2_ adsorption energies of −0.75 and −1.06 eV, respectively, bracketing the value for the “tilted” metal-free **3b** complex. Note that the **Pd3b** → **Pd3aV** transition is associated with the reduction of the Pd centre from Pd^+^ to Pd^0^.

In summary, carbonate CO_3_^2−^ species show coordination patterns different from CO and CO_2_ moieties: they tie to the ceria support or metal centres via O atoms, whereas the C atom does not participate in adsorbate–substrate interaction. From the comparison of CO_2_ binding energies at the metal-free and M-containing sites, it follows that CO_2_ binds slightly more strongly in MCO_3_/NP[0/2] structures than in CO_3_/NP[0/2]. Conversely, the creation of an extra O vacancy stronger stabilises the formation of the carbonates at metal-free NPs than that at metal-containing NPs.

### 3.2. Reaction Energies

In this section, we consider the energies of CO to CO_2_ oxidation, E*_ox_, and of CO_2_ to CO_3_ transformation, E*_CO3_, along with the corresponding activation barriers, *E^≠^*, calculated for Pd/NP and Ag/NP models in comparison with the bare NP model. Data in Table 2 show that i) all these reactions are exothermic and ii) activation barriers for the carbonate formation are often higher than for the oxidation of CO.

Bare NPs adsorb CO very weakly, by less than 0.3 eV (Table 1, Figure 2). The extraction of lattice O and the formation of ceria-supported CO_2_ yield energies of 0.9–1.4 eV and require 0.8–1.0 eV of activation (Table 2). Because of the low CO_2_ desorption barriers, 0.15–0.25 eV (Table 1, *E_des_(CO_2_)* = −*E_b_(CO_2_)* for **2a** and **2aV**), the formed CO_2_/Ce_21_O_42_ and CO_2_/Ce_21_O_41_ complexes can quite easily decompose. Otherwise, they are expected to exothermically transform into carbonates with activation barriers ranging from low—0.27 eV (for CO_2_/NP[0/1])—to moderate—0.82 eV (for CO_2_/NP[1/1])—values. The thermodynamic stability of such surface carbonate complexes makes them most probable candidates for experimental detection [68].

CO binds very weakly at the defect-free PdO_4_ site of the Pd/NP[0/0] complex with an adsorption energy of −0.1 eV (**Pd1b** in Table 1). CO_2_ can form via the interaction of the adsorbed CO with a lattice O atom. The process, which is exothermic by 1.3 eV, requires the overcoming of a barrier of 0.5 eV (Table 2). The formed CO_2_ molecule is quite strongly bound, with a desorption energy of 1.2 eV (Table 1, *E_des_(CO_2_)* = −*E_b_(CO_2_)* for **Pd2a**). Overcoming an even higher barrier of 1.8 eV (Table 2) is needed to extract one more lattice O centre and activate the transformation of CO_2_ to CO_3_^2−^, which is exothermic by only 0.25 eV. CO is strongly, by 1.7 eV, adsorbed at the O-deficient PdO_3_ site (**Pd1aV** in Table 1). The formed stable O_3_PdCO species can transform to O_2_PdCO_2_ with an activation barrier of 1.1 eV and moderate reaction exothermicity of 0.6 eV (Table 2). Slightly exothermic by 0.3 eV, the formation of carbonate is hindered by a high energy barrier of 1.8 eV. A much lower energy barrier of 0.7 eV (−*E_b_(CO_2_)* for **Pd2aV** in Table 1) is required to desorb the CO_2_ molecule into the gas phase. Thus, the most likely ceria-supported Pd-intermediates to be detected in reaction medium are saturated square-planar O_3_PdCO/NP (**Pd1aV**) and O_3_PdCO_2_/NP (**Pd2a**) complexes whose formation proceeds with a sizable energy release (1.3–1.7 eV) and moderate activation barriers of 0.5 eV and whose decomposition is hindered by substantial barriers of about 1.1–1.2 eV.

The reactivity of Ag-containing systems is different (Table 2). CO adsorption on defect-free and O-deficient Ag/NP systems occurs with a moderate energy gain of 0.8 eV (Table 1). The formed O_4_AgCO and O_3_AgCO species are converted into the corresponding O_3_AgCO_2_ and O_2_AgCO_2_ species with a similar exothermicity around 0.4 eV, but the activation barrier for the more O-saturated complex O_4_AgCO, 0.9 eV, is 0.4 eV higher than that for O_3_AgCO (Table 2). Despite the high exothermicity of 1.2–1.5 eV, the transformation to carbonates is hindered by barriers of ~0.8–1.7 eV. Alternatively, the decomposition with CO_2_ desorption should proceed quite readily (see −*E_b_(CO_2_)* values for **Ag2b** and **Ag2aV** in Table 1). Thus, the carbonyl complexes O_4_AgCO/NP (**Ag1a**) and O_3_AgCO (**Ag1aV**), which are easily formed with notable energy gains, are expected to be detectable in a reaction medium. The detection of carboxylate AgCO_2_/NP (**Ag2b** and **Ag2aV**) complexes seems problematic due to their instability with respect to CO_2_ desorption.

We estimated the propensity of CO to CO_2_ transformation by the energy of the CO(gas) + M/NP[*n*/0] → CO_2_(gas) + M/NP[*n*/1] oxidation reaction, *E_ox_* (Equations (5) and (6)). It is directly connected with the ease of O release from the ceria lattice and O vacancy creation (Table 2).

Let us compare the oxidation reaction energy *E_ox_* calculated for the O_4_-site of CeO_2_ NP with the calculated energies for clean ceria surfaces [65,71,74,79,80] and the Pd_1_–ceria interfaces [8,9,20]. This reaction was found to be slightly, by 0.4–0.6 eV, exothermic on bare CeO_2_(111) [74,79] and notably more exothermic on CeO_2_(110) [65,74,78,79], at 1.1–1.8 eV. The calculated reaction exothermicity further drastically increases to 3.1 eV for the CeO_2_(100) surface with the most exposed O atoms [71]. Our calculated *E_ox_* energies for the stoichiometric and O-deficient NP{100} sites, −1.44 and −1.01 eV, respectively (Table 2), are considerably lower than those for the CeO_2_(100) surface, but in a similar range to that for CeO_2_(110) (assuming that the *E_ox_* value should increase by ca. 0.5 eV when the U value is increased from 4 to 5 eV [62]).

At the Pd_1_/CeO_2_(100) and O_1_Pd_1_/CeO_2_(100) sites, the conversion of CO to CO_2_ was characterised by energy yields of 0.6 and 1.2 eV [9], respectively—markedly lower than at the pristine CeO_2_(100) surface. The reaction is also moderately exothermic at the Pd_1_/CeO_2_(110) interface, by 1.2 eV [20], and highly exothermic at the isolated O_1_Pd_1_ and O_2_Pd_1_ species on the CeO_2_(111) surface, by 2.9 and 2.7 eV [8]—much more exothermic than at the regular CeO_2_(111) surface because of exposing weakly bonded O atoms. Compared to the above-mentioned transformations, that at Pd/NP[0/0] has the lowest *E_ox_* of −0.23 eV. There, the formal migration of an O-atom of the O_4_Pd moiety to become a part of CO_2_ molecule is difficult even in comparison with the three-coordinated O centre of the regular CeO_2_(111) surface. For the conversion at the Pd/NP[1/0] site with *E_ox_* = −1.61 eV, the relocation of the second O atom of the O_4_Pd moiety is more favourable than of the O atom of the isolated O_1_Pd_1_ moiety on CeO_2_(100) or Pd_1_ at CeO_2_(110), but less beneficial than that of O_1_Pd_1_ or O_2_Pd_1_ ad-species on CeO_2_(111). For the reactions on Ag/NP[*n*/0] sites, the energy *E_ox_*, −1.0–1.2 eV, is slightly lower than on the Pd/NP[1/0] site, thus approaching *E_ox_* values for O_1_Pd_1_/CeO_2_(100) and Pd_1_/CeO_2_(110) systems. Therefore, our model ceria particle with the Pd atom adsorbed on the O-defective {100} nanofacet appears to be more reactive in CO to CO_2_ oxidation than its formal analogue of the Pd-doped extended CeO_2_(100) surface.

Thus, the trend for lowering *E_ox_* in bare and M-containing ceria systems is Pd/NP[1/0] > NP[*n*/0] ≈ Ag/NP[*n*/0] > Pd/NP[0/0], which correlates with the growth of the O vacancy formation energy in the same row (Table 2). Note that the reactivity in CO oxidation of both Ag/NP complexes—with and without an O vacancy near the Ag atom—is similar. Conversely, the presence of an O vacancy in the vicinity of Pd atom is mandatory for CO oxidation at Pd/NP systems to proceed. This makes two consequent steps of CO oxidation at the Pd/Ce_21_O_42_ nanoparticle problematic. Such characteristics of the studied models as moderately strong CO adsorption, exothermic overall CO oxidation process, sufficiently low barriers of MCO to MCO_2_ transformations, and ease of CO_2_ desorption render CO oxidation by lattice ceria oxygen atoms more favourable at the sites with Ag than with Pd. Comparing the reaction and activation energies of CO to CO_2_ and CO_2_ to CO_3_ conversions for M-containing ceria NPs, we conclude that the most probable species to be observed experimentally are AgCO and PdCO carbonyls and carboxylate PdCO_2_ species. Unlike purely ceria nanoparticles, the formation of silver and palladium carbonates is prohibited by high activation barriers. We note, however, that for precise information on the species present in the reaction medium at equilibrium, a microkinetic modelling is required, which is out of the scope of the present study.

### 3.3. CO_x_ Vibrational Fingerprints

Several studies reported measured [7,9,16,25,26,28,68,81] and calculated [8,9,68,82,83,84] vibrational frequencies of CO, CO_2_, and CO_3_^2−^ groups in the complexes formed by the interaction of CO or CO_2_ with cerium-based substrates. The calculated frequencies for selected stretching vibrations of CO_x_ subsystems of the structures displayed in Figure 2, Figure 3 and Figure 4 are collected in Table 3.

Our modelling revealed that the CO stretching frequency, ν(CO), for the molecule attached to a cerium ion in M-free systems **1a** and **1aV** and Pd-containing model **Pd1a** shifts by 27–34 cm^−1^ to the short-wave region, which agrees with the measured blue shifts of 27–32 cm^−1^ (vs. 2143 cm^−1^ for free molecule [85]) for CO interacting with the Ce^4+^ centres of the nanostructured CeO_2_ [19,86]. Note that the quantitatively precise reproduction of measured vibrational frequencies of CO on ceria requires going beyond the U-corrected generalised-gradient exchange-correlation functionals to hybrid-type functionals [81]. In contrast, ν(CO) for the fragments with M_1_-CO bonding formed on M/NP[*n*/0] substrates shows redshifts of 50–113 cm^−1^, consistent with the C-O bond elongation by 1–2 pm. Among the models containing Pd_1_ species, the **Pd1b** complex with Pd^2+^ cation coordinated by four two-coordinated O anions reveals a medium redshift of 84 cm^−1^, and the maximum redshifts of 109–113 cm^−1^ are identified for the other two complexes, **Pd1c** and **Pd1aV**, with three-fold coordinated Pd^+^ and Pd^2+^ cations (Table 3). For the earlier examined systems with Pd-CO bonds, the redshifts Δν(CO) were calculated to increase from 6 to 96 cm^−1^ in the order O_2_Pd_1_/CeO_2_(111) < O_1_Pd_1_/CeO_2_(100) ≈ O_1_Pd_1_/CeO_2_(111) < Pd_1_/CeO_2_(100) < Pd_1_/CeO_2_(111) [8,9]. These values are comparable with those attributed to the CO molecule contacting one Pd atom in experimental studies of Pd/CeO_2_, 13–123 cm^−1^ [7,28] and 10–69 cm^−1^ [9,16,25] and PdO/CeO_2_, 58 cm^−1^ [26]. Note that the Δν(CO) redshifts for CO coordinated to the supported Pd cations are opposite to the blue shift for the free PdCO^+^ ion, measured at 63 cm^−1^ and calculated at 75 cm^−1^ (B3LYP), but approach a redshift for the neutral PdCO molecule, measured at 87 cm^−1^ and calculated at 99 cm^−1^ [70]. The redshifts of ν(CO) for **Ag1a** and **Ag1aV** complexes, at 50 and 61 cm^−1^, respectively, are smaller than those for the Pd_1_-CO moieties and correspond to about one-third of the calculated redshift for the AgCO molecule, 144 cm^−1^ [87]. Similarly large frequency redshifts to those obtained for CO on single Ag and Pd cations on a ceria NP were calculated for CO adsorbed on Pt^+^ and Pd^2+^ cations anchored to ceria [83].

The highest frequencies of CO_2_ stretching vibrations in **2a**, **2aV**, **Ag2a**, **Ag2bV**, **Pd2b**, and **Pd2bV** complexes with a linear CO_2_ moiety are close to that of the IR active CO_2_ asymmetric stretching frequency of the free molecule; the negative shift Δν(CO_2_) does not exceed 40 cm^−1^ (Table 3). The redshift Δν(CO_2_) for a bent CO_2_ fragment in **2bV**, 706 cm^−1^, is comparable to the experimental value for the free anion CO_2_^−^, 691 cm^−1^ [88] (vs. measured ν(CO_2_) of free molecule 2349 cm^−1^ [76]) and CO_2_^−^ species at TiO_2_ surface, 709 cm^−1^ [89]. The redshift Δν(CO_2_) for the MCO_2_/NP[*n*/1] systems having the M-C bond from 413 to 1031 cm^−1^ is associated with the bending of the CO_2_ moiety and C-O bond elongation by 3 to 14 pm. The redshifts for PdCO_2_/NP systems, 828 cm^−1^ (**Pd2a**) and 735 cm^−1^ (**Pd2aV**), are in the range of values 610–850 cm^−1^ reported for coordination compounds with CO_2_ attached to a single d-metal atom [76], while redshift Δν(CO_2_) in **Ag2b** is smaller, at only 413 cm^−1^. Δν(CO_2_) for the CO_2_^2−^ group in **Ag2aV** with CO bonds elongated by 14 pm compared to those of free CO_2_ matches the measured redshifts of 1020–1079 cm^−1^ for carbonite ions at CeO_2_ [90] and in Cs_2_CO_2_ [91]. Thus, the increased redshift Δν(CO_2_) seems to correlate with C-O bond elongation, **Ag2b** < **Pd2aV < Pd2a < Ag2aV** (Table 1).

Differences in the length of the intramolecular C-O bonds are considered among the main factors determining the frequency splitting of CO_3_^2−^ stretching vibrations [68]. In tridentate CO_3_/NP[*n*/2] and MCO_3_/NP[*n*/2] complexes, the C-O bond lengths are 125–135 pm for the 1.11- and 1.21-modes, and in less symmetrical bidentate 1.20- and 1.30-structures, the C-O bonds cover a wider interval 121–146 pm.

Overall, the splitting of C-O frequencies is lower for more symmetrical species. In particular, for the two highest frequencies (ν_1_ and ν_2_), it is only 47 cm^−1^ for 2.2.2-structures, increasing to 97–133 cm^−1^ for 2.2.1-isomers and 162–252 cm^−1^ for 1.2.1- and 1.1.1-isomers (Table 3). For 1.20- and 1.30-carbonates, the splitting becomes as high as 563–632 cm^−1^. According to earlier calculations, carbonate groups attached to nanostructured ceria surface by three oxygen atoms, represented by sets of 1.21-, 1.2.1- and 1.3.1-structures, are characterised by ν_1_ values from 1590 to 1490 cm^−1^ [68]. With the inclusion of ν_1_ values for tridentate structures **3b** and **3aV**, this range extends to 1400 cm^−1^. The calculated ν_1_ for tridentate carbonates corresponds to a broad experimental region of 1620–1450 cm^−1^ attributed to the high-frequency vibrations of the carbonate groups formed on surfaces with oxygen vacancies, as well as on facet, edge, and corner sites of ceria particles [68]. The ν_1_ frequencies for tridentate carbonate **Ag3a**, **Pd3a**, **Pd3b**, **Ag3aV**, and **Pd3aV** complexes, of 1573–1434 cm^−1^, fall between the limits of the clean surface of the nanostructured ceria. The ν_1_ values for 1.20-carbonate groups in **3a** and **3bV** systems are in a narrow range of 1718–1698 cm^−1^ for bidentate carbonates [68] corresponding to the measured frequency interval of 1732–1722 cm^−1^ [68]. The ν_1_ frequencies of the M-containing 1.30-complexes **Pd3c, Pd3bV, Ag3b,** and **Ag3bV** are in a broader range of 1752–1689 cm^−1^, which includes the interval for bidentate CO_3_^2−^ on M-free ceria substrates.

The calculated ν_2_ frequency range of the complexes on the clean ceria surface is 1353–1227 cm^−1^. This corresponds to the experimental frequencies of 1380 and 1280 cm^−1^ [68]. The ν_2_ values for tridentate 1.21- and 2.2.1-carbonates **P3b, Pd3aV, Ag3a,** and **Ag3aV** for M-containing systems, at 1340–1220 cm^−1^, are between these limits, and that for the 1.11-**Pd3a** isomer is only 6 cm^−1^ below the low-end threshold. The ν_2_ frequencies are distributed between 1194 and 1082 cm^−1^ for bidentate carbonates depicted in Figure 2 and those examined in [68]; the related experimental values are 1147–1133 cm^−1^ [68].

The range of ν_2_ for the CO_3_ moieties coordinated in a bidentate way at M/NP systems is 1170–1120 cm^−1^. Thus, the frequency ranges are similar for the metal-free CO_3_/NP and MCO_3_/NP sites, making the discrimination between the metal-containing and bare ceria particles solely on the basis of the vibrational spectroscopy data problematic.

In summary, our calculations show that the C-O stretching vibrations of the ceria-supported PdCO and AgCO fragments feature redshifts up to ~110 cm^−1^, which is at variance with the blue shift at metal-free ceria. Redshifts of CO_2_ asymmetric stretching frequency of the M-CO_2_ fragments are much higher, up to ~830 cm^−1^ for carboxylate MCO_2_ and further increasing by ~200 cm^−1^ for carbonite AgCO_2_. The two highest ν(CO_3_) stretching frequencies of M-CO_3_ structures lie in intervals 1755–1690 cm^−1^ and 1170–1120 cm^−1^ for the CO_3_ moiety coordinated in a bidentate fashion and 1575–1430 cm^−1^ and 1340–1220 cm^−1^ for the CO_3_ in the tridentate coordination.

## 4. Conclusions

CO_x_ intermediates formed upon CO adsorption and oxidation on single M = Pd and Ag atoms coordinated to the O_4_-site on the {100} facet of a Ce_21_O_42_ nanoparticle have been studied computationally. Equilibrium structures, CO_x_ vibrational frequencies, and energetic parameters of various MCO_x_-containing complexes have been determined. The influence of the creation of an O vacancy nearby the M atom has been also investigated. The stability of the CO_x_ moieties anchored to the ceria-supported M atom is found to increase in the order MCO < MCO_2_ < MCO_3_, similar to the trend for CO_x_ species adsorbed on M-free ceria NP.

Except for the Pd atom saturated by four O atoms of the ceria surface O_4_-site, which is unable to properly adsorb CO, the doping of the ceria nanoparticle with Pd and Ag atom increases its propensity to bind the CO molecule with respect to bare ceria material. In particular, the CO adsorption energy value reaches −1.7 eV for a PdCO unit on a ceria nanoparticle with a nearby O vacancy. CO binding in AgCO complexes, regardless of the presence or absence of a nearby O vacancy, is moderately strong, at −0.8 eV. All these species are the most probable candidates to be detected experimentally, also due to the presence of moderate barriers for CO oxidation (0.5–1.0 eV). In contrast to the blue shift for CO adsorbed on pristine ceria, red shifts of the C-O stretching (vs. free CO) have been calculated for MCO species anchored to ceria. The red shifts of the CO stretching frequency are higher for complexes of Pd and increase with the decreasing coordination of M from MO_4_ to MO_3_ for a particular metal: Ag/Ce_21_O_42_ (50 cm^−1^) < Ag/Ce_21_O_41_ (61 cm^−1^) < Pd/Ce_21_O_42_ (84 cm^−1^) < Pd/Ce_21_O_41_ (113 cm^−1^).

Carboxylate CO_2_^−^ and carbonite CO_2_^2−^ (for Ag-doped NP with an O vacancy) complexes featuring a bent CO_2_ moiety are formed upon CO oxidation at the M/ceria interface. Contrary to AgCO_2_-species, which are easily decomposed via CO_2_ detachment, PdCO_2_ moieties are prone to withstand decomposition due to significant CO_2_ desorption energies of 0.7–1.2 eV. These PdCO_2_ moieties anchored to ceria particles could be experimentally detected by the red shifts of the CO_2_ asymmetric stretching frequency (vs. that of free CO_2_ molecule) by 828 cm^−1^ (one O vacancy nearby Pd) and 735 cm^−1^ (two O vacancies nearby Pd).

Unlike pristine ceria, carbonate structures at ceria-supported Pd and Ag atoms are hardly formed before CO_2_ desorption due to the high barriers of CO_2_ transformation to CO_3_^2−^ (up to 1.8 eV for PdCO_3_ moieties) and weak CO_2_ binding (below ~0.2 eV for AgCO_3_ moieties). Detailed analysis of the vibrational spectra of MCO_3_/NP complexes has shown that the two highest ν(CO_3_) stretching frequencies lie in the well-resolved intervals 1755–1690 and 1170–1120 cm^−1^ for the CO_3_ moiety coordinated in a bidentate fashion and 1575–1430 and 1340–1220 cm^−1^ for the carbonate groups in the tridentate coordination. These frequency ranges are similar to those for the M-free CO_3_/NP sites. Thus, discrimination between the M-containing and bare ceria particles solely using vibrational spectroscopy data seems hardly possible.

In summary, such characteristics of the studied models as moderately strong CO adsorption, an exothermic CO oxidation process, sufficiently low barriers of MCO to MCO_2_ transformations, and ease of CO_2_ desorption render CO oxidation by lattice ceria oxygen atoms more favourable at the sites with Ag than with Pd.

## Figures and Tables

**Table 1:** Calculated parameters of the surface complexes shown in Figure 2, Figure 3 and Figure 4, created by the interaction of CO with the pristine ceria Ce_21_O_42−*n*_ and metal–ceria M/Ce_21_O_42−*n*_ sites (M = Pd, Ag; *n* = 0, 1): interatomic distances—r, number of the Ce^3+^ ions—N, difference between the numbers of spin-up and spin-down electrons—m, binding energies of CO—*E_b_(CO)* (Equation (3)) and CO_2_–*E_b_(CO_2_)* (Equation (4)). Negative energy values correspond to exothermic processes.

System *^a^*	r(M-C)	r(M-O)	r(Ce-O) *^b^*	r(C-O) *^c^*	N	m	*E_b_(CO)*	*E_b_(CO_2_)*
	pm	pm	pm	pm			eV	eV
Complexes with CO								
**Pd1a**	296 *^d^*	-	-	114	2	2	−0.26	-
**Pd1b**	241	-	-	115	2	2	−0.13	-
**Pd1c**	188	-	-	116	1	0	0.13	-
**Pd1aV**	187	-	-	116	4	4	−1.74	-
**Ag1a**	201	-	-	115	1	1	−0.80	-
**Ag1aV**	198	-	-	115	3	3	−0.78	-
**1a**	293 *^d^*	-	-	114	0	0	−0.26	-
**1aV**	297 *^d^*	-	-	114	2	2	−0.23	-
Complexes with CO_2_								
**Pd2a**	206	-	247	2 × 127	2	0	−1.43	−1.20
**Pd2b**	-	-	314	2 × 118	2	0	−0.41	−0.18
**Pd2aV**	193	237	268	129; 124	4	4	−2.33	−0.72
**Pd2bV**	330	-	321	2 × 118	4	4	−1.77	−0.16
**Ag2a**	356	-	316	2 × 118	3	3	−1.37	−0.16
**Ag2b**	234	-	268	122; 121	3	1	−1.22	−0.01
**Ag2aV**	207	-	249	2 × 132	3	1	−1.13	−0.10
**Ag2bV**	342	-	348	2 × 118	5	1	−1.10	−0.07
**2a**	-	-	300	2 × 118	2	0	−1.69	−0.25
**2aV**	-	-	310	2 × 118	4	2	−1.16	−0.15
**2bV**	-	-	254	2 × 125	3	2	−0.42	0.59
Complexes with CO_3_^2−^								
**Pd3a**	249	207	260	2 × 133; 125	4	2	−1.68	−1.44
**Pd3b**	265	209	248	129; 135; 129	3	2	−0.99	−0.75
**Pd3c**	-	212	245	133; 143; 121	2	0	−0.74	−0.50
**Pd3aV**	275	211	258	132; 132; 128	4	2	−2.67	−1.06
**Pd3bV**	288	211	247	131; 143; 122	4	0	−2.28	−0.67
**Ag3a**	277	221	245	130; 135; 127	3	1	−2.72	−1.49
**Ag3b**	289	233	247	132; 141; 122	3	1	−2.09	−0.86
**Ag3aV**	273	216	265	130; 132; 130	5	1	−2.36	−1.40
**Ag3bV**	291	211	255	129; 146; 122	5	1	−2.09	−1.13
**3a**	-	-	237	133; 138; 122	2	0	−2.59	−1.15
**3b**	-	-	250	129; 132; 129	2	2	−2.28	−0.84
**3aV**	-	-	262	130; 131; 130	4	2	−3.23	−2.22
**3bV**	-	-	229	133; 137; 122	4	2	−2.53	−1.52

*^a^* For notations, see Figure 2, Figure 3 and Figure 4; *^b^* Average distances of the O of CO_x_ moiety and nearest neighbour Ce atom; ^*c*^ Bond lengths within CO_x_ moiety; *^d^* Ce-C contact.

**Table 2:** Reaction and activation energies (*E^≠^*) on the pristine ceria Ce_21_O_42−*n*_ and metal–ceria M/Ce_21_O_42−*n*_ models (M = Pd, Ag; *n* = 0, 1) depicted in Figure 2, Figure 3 and Figure 4. Oxygen vacancy formation energies *E(O_V_*) are also shown. Negative energy values correspond to exothermic processes. All energies are in eV.

Model *^a^*		CO → CO_2_			CO_2_ → CO_3_	
	*E(O_V_)*	*E_ox_ *^b^**	E*_CO2_ *^c^*	*E^≠^*	E*_CO3_ *^d^*	*E^≠^*
Ce_21_O_42_	1.87	−1.44	−1.43	1.02	−0.90	0.27
Ce_21_O_41_	2.31	−1.01	−0.92	0.82	−2.07	0.82
Ag/Ce_21_O_42_	2.08	−1.21	−0.52	0.88	−1.50	0.78
Ag/Ce_21_O_41_	2.28	−1.03	−0.35	0.53	−1.23	1.73
Pd/Ce_21_O_42_	3.08	−0.23	−1.30	0.51	−0.25	1.83
Pd/Ce_21_O_41_	1.70	−1.61	−0.59	1.06	−0.34	1.80

*^a^* System on which initial CO adsorption takes place; *^b^* reaction energy of the CO oxidation CO(gas) + NP[*n*/0] → CO_2_(gas) + NP[*n*/1] or CO(gas) + M/NP[*n*/0] → CO_2_(gas) + M/NP[*n*/1]; *^c^* reaction energy of the CO_2_ formation CO/NP[*n*/0] → CO_2_/NP[*n*/1] or MCO/NP[*n*/0] → MCO_2_/NP[*n*/1]; *^d^* reaction energy of CO_2_/NP[*n*/1] → CO_3_/NP[*n*/2] or MCO_2_/NP[*n*/1] → MCO_3_/NP[*n*/2] conversion.

**Table 3:** Calculated vibrational frequencies ν(CO), ν(CO_2_) and ν(CO_3_) for the systems depicted in Figure 2, Figure 3 and Figure 4 along with the corresponding frequency shifts Δν(CO) and Δν(CO_2_) with respect to calculated vibrational frequencies of free molecules CO (ν(free CO) = 2131 cm^−1^) and CO_2_ (ν_asym_(free CO_2_) = 2363 cm^−1^).

System *^a^*	ν(CO) cm^−1^	Δν(CO) cm^−1^	ν(CO_2_) cm^−1^	Δν(CO_2_) cm^−1^	ν(CO_3_) cm^−1^
Complexes with CO					
**Pd1a**	2158	27	-	-	-
**Pd1b**	2047	−84	-	-	-
**Pd1c**	2022	−109	-	-	-
**Pd1aV**	2018	−113	-	-	-
**Ag1a**	2081	−50	-	-	-
**Ag1aV**	2070	−61	-	-	-
**1a**	2162	31	-	-	-
**1aV**	2165	34	-	-	-
Complexes with CO_2_					
Linear O-C-O					
**Pd2b**	-	-	2323	−40	-
**Pd2bV**	-	-	2332	−31	-
**Ag2a**	-	-	2348	−15	-
**Ag2bV**	-	-	2350	−13	-
**2a**	-	-	2354	−9	-
**2aV**	-	-	2360	−3	-
**Bent O-C-O**					
**Pd2a**	-	-	1535	−828	-
**Pd2aV**	-	-	1628	−735	-
**Ag2aV**	-	-	1332	−1031	-
**Ag2b**	-	-	1950	−413	-
**2bV**	-	-	1657	−706	-
Complexes with CO_3_^2−^					
Tridentate					
**Pd3a**	-	-	-	-	1573; 1221
**Pd3b**	-	-	-	-	1451; 1243
**Pd3aV**	-	-	-	-	1442; 1309
**Ag3a**	-	-	-	-	1511; 1233
**Ag3aV**	-	-	-	-	1434; 1337
**3b**	-	-	-	-	1442; 1280
**3aV**	-	-	-	-	1400; 1353
**Bidentate**					
**Pd3c**	-	-	-	-	1746; 1118
**Pd3bV**	-	-	-	-	1729; 1122
**Ag3b**	-	-	-	-	1689; 1126
**Ag3bV**	-	-	-	-	1752; 1169
**3a**	-	-	-	-	1714; 1082
**3bV**	-	-	-	-	1704; 1101

***^a^*** For system designations see Figure 2, Figure 3 and Figure 4.

## Data Availability

Data is contained within the article or Supplementary Material.

## Supplementary Materials

The following data are available online at https://www.mdpi.com/article/10.3390/ma14226888/s1: Coordinates of the atomic positions of all considered Ce_21_O_42−δ_, CO_x_/Ce_21_O_42−δ_, AgCO_x_/Ce_21_O_42−δ_ and PdCO_x_/Ce_21_O_42−δ_ complexes and selected transition state structures connecting these complexes along with their total energies.
