# Supplementary material for: Adsorption and Oxidation of CO on Ceria Nanoparticles Exposing Single-Atom Pd and Ag: A DFT Modelling
Source: Materials (Basel). 2021 Nov 15;14(22):6888. doi: 10.3390/ma14226888 (PMC8618484; doi:10.3390/ma14226888)
Supplement: Supplementary file 1 [file materials-14-06888-s001.zip › materials-1452603-supplementary.pdf]

Adsorption and oxidation of CO on ceria nanoparticles exposing single-atom Pd and Ag: A DFT modelling

Vladimir A. Nasluzov[1], Elena A. Ivanova-Shor[1*], Aleksey M. Shor[1],
Svetlana S. Laletina[1], Konstantin M. Neyman[2,3]

[1] *Institute of Chemistry and Chemical Technology SB RAS, Federal Research Center "Krasnoyarsk Science Center SB RAS", Krasnoyarsk, Russia*

[2] *Universitat de Barcelona, Barcelona, Spain*

[3] *Institució Catalana de Recerca i Estudis Avançats (ICREA), Barcelona, Spain*

* Corresponding author, e-mail: eshor1977@gmail.com

XYZ coordinates of all intermediates and transition states and the total energies, *E*, and the formation energies, E^f, in eV.

Structures at Fig. 1

63

Structure **A**, E=-494.877 eV

Ce	13.557920	8.187340	9.958100
Ce	12.370300	14.866019	7.352960
Ce	14.029900	14.451441	10.466480
Ce	10.831720	6.264740	11.169519
Ce	5.714620	8.094900	7.939200
Ce	9.065080	7.281600	14.238340
Ce	9.291200	13.276700	8.351820
Ce	9.995480	9.995520	9.997900
Ce	7.267040	7.150580	11.085700
Ce	5.785320	8.652600	14.091200
Ce	11.291879	12.039160	5.485140
Ce	6.528740	11.331660	9.561820
Ce	11.302680	12.783279	12.131259
Ce	9.115820	6.687200	7.945060
Ce	14.645880	11.204979	11.772360
Ce	13.437340	11.498200	8.364700
Ce	11.945961	9.206120	13.162020
Ce	4.129420	9.050940	10.974760
Ce	11.847640	8.617560	6.736160
Ce	8.542680	10.842340	13.339980
Ce	8.424620	10.070280	6.538880
O	14.117060	13.571780	8.279180
O	6.953060	6.488500	8.915780
O	13.088660	15.842820	9.123740
O	9.587220	12.720480	13.426460

O	4.548880	11.030700	10.380239
O	11.168300	7.078860	13.397440
O	7.777300	8.545320	12.887640
O	12.897739	6.240220	10.583040
O	7.567340	7.954780	6.630680
O	9.188980	12.243119	6.202280
O	12.916360	10.694580	6.286420
O	10.758520	6.771480	6.559400
O	13.986480	9.061400	12.145140
O	7.179980	7.305380	15.195740
O	13.121620	12.306740	10.532579
O	12.068260	9.839040	8.877720
O	10.302340	12.435760	10.158640
O	12.889600	11.328540	13.156240
O	9.848920	8.863440	7.843260
O	11.552240	8.439520	11.053261
O	9.161560	7.796020	10.153641
O	6.224480	8.921860	9.975480
O	15.477240	13.130420	11.532580
O	7.253540	13.288701	9.046600
O	12.506599	14.565420	11.921160
O	6.722620	10.505840	14.458580
O	4.091980	9.502520	13.080320
O	14.759060	10.236760	9.741160
O	12.171839	13.953919	5.326960
O	7.985620	10.808600	11.174520
O	3.870520	8.102520	8.975520
O	10.533320	10.554240	12.112221
O	10.387900	9.946480	5.388580
O	8.629640	11.020560	8.531660
O	10.000620	9.300180	14.431280
O	13.483840	7.798020	7.854040
O	10.329620	15.099660	7.840640
O	10.079780	5.398920	9.360400
O	11.573400	12.690800	7.621220
O	5.367520	7.405300	12.127800
O	6.298680	10.199680	7.476640
O	8.793100	6.040200	12.378860

64

Structure **B**, E= -497.317 eV

Ce	13.551496	8.253419	9.957032
Ce	12.374371	14.895145	7.380422
Ce	14.036007	14.480094	10.487561
Ce	10.778784	6.292459	11.189318
Ce	5.662479	8.085333	7.974170
Ce	9.061226	7.289093	14.259968
Ce	9.285367	13.272213	8.305039
Ce	9.958933	10.021826	9.953455
Ce	7.229442	7.157780	11.112503

Ce	5.778247	8.662179	14.122688
Ce	11.332073	12.084453	5.474396
Ce	6.512558	11.323154	9.525644
Ce	11.295829	12.779497	12.101936
Ce	9.033065	6.732654	7.948868
Ce	14.651276	11.227276	11.788775
Ce	13.446344	11.536362	8.368412
Ce	11.934996	9.222339	13.156377
Ce	4.113197	9.073362	11.015468
Ce	11.809834	8.698835	6.713281
Ce	8.537539	10.840894	13.314872
Ce	8.353570	10.094615	6.421198
O	14.135128	13.604934	8.311796
O	6.932932	6.461713	8.987356
O	13.114294	15.877814	9.169789
O	9.579072	12.717879	13.399632
O	4.556655	11.044325	10.424205
O	11.163992	7.075598	13.383245
O	7.773163	8.541678	12.909619
O	12.883520	6.199645	10.483342
O	7.490124	7.828268	6.676840
O	9.277039	12.364591	6.154122
O	12.974550	10.716537	6.320851
O	10.805072	6.710032	6.591334
O	13.982947	9.062092	12.130977
O	7.195532	7.309952	15.228738
O	13.138571	12.319069	10.545806
O	12.085370	9.832413	8.861809
O	10.314578	12.403460	10.126958
O	12.886733	11.327064	13.159039
O	9.845788	8.843628	7.811168
O	11.556946	8.427895	11.047843
O	9.154236	7.765889	10.149398
O	6.203158	8.910391	10.010568
O	15.482976	13.141584	11.574045
O	7.243349	13.283607	9.003506
O	12.494003	14.565270	11.931218
O	6.734815	10.514046	14.462001
O	4.087816	9.515421	13.137927
O	14.761884	10.240206	9.746587
O	12.235051	14.016826	5.359632
O	7.999819	10.781815	11.143148
O	3.820311	8.100451	9.057912
O	10.532026	10.545576	12.092800
O	10.527211	9.978217	5.330973
O	8.607297	11.029880	8.497370
O	10.007083	9.303246	14.429375
O	13.498166	7.763596	7.832885
O	10.347145	15.117681	7.883344
O	10.090159	5.354203	9.344687

O	11.605835	12.712971	7.628624
O	5.346903	7.411480	12.183086
O	6.156331	10.163509	7.524675
O	8.794586	6.039888	12.387471
Ag	12.202901	5.761657	8.236834

64

Structure **C**, E=-500.606 eV

Ce	13.490504	8.307867	9.993598
Ce	12.363608	14.924132	7.308831
Ce	14.049385	14.506152	10.405477
Ce	10.648128	6.392592	11.283683
Ce	5.542296	8.233103	8.033115
Ce	8.950419	7.390933	14.317540
Ce	9.296517	13.304919	8.302363
Ce	9.929404	10.092396	10.016760
Ce	7.104542	7.304438	11.187100
Ce	5.712832	8.873580	14.181508
Ce	11.270721	12.094505	5.436841
Ce	6.505766	11.441842	9.565138
Ce	11.353648	12.802892	12.103435
Ce	8.876043	6.828765	8.019152
Ce	14.673741	11.253936	11.722409
Ce	13.408889	11.565406	8.327428
Ce	11.976712	9.257939	13.282454
Ce	4.032441	9.282945	11.078657
Ce	11.716757	8.745927	6.732090
Ce	8.566605	10.928133	13.361495
Ce	8.272192	10.162119	6.436816
O	14.114925	13.624164	8.217070
O	6.821314	6.580749	9.051623
O	13.110236	15.904024	9.070567
O	9.653994	12.786930	13.426988
O	4.535551	11.226526	10.453573
O	11.010600	7.013954	13.419248
O	7.690259	8.666707	12.947444
O	10.789305	6.643735	6.750759
O	7.381952	7.905162	6.720741
O	9.231969	12.391807	6.134072
O	12.903221	10.683575	6.293550
O	14.074844	9.080436	12.029092
O	7.066225	7.480242	15.279330
O	13.147558	12.349180	10.475112
O	12.059760	9.822707	8.831022
O	10.341043	12.406510	10.106415
O	13.004097	11.465116	13.102714
O	9.748851	8.887884	7.805170
O	11.533183	8.446184	11.088643
O	9.073399	7.810441	10.187266

O	6.117507	9.052362	10.053434
O	12.724388	6.161773	10.319270
O	15.508300	13.182124	11.445607
O	7.270190	13.380380	9.037235
O	12.522331	14.609145	11.853008
O	6.719854	10.696509	14.477319
O	4.029233	9.757270	13.178194
O	14.714414	10.235144	9.634151
O	12.175126	14.003952	5.286401
O	8.004259	10.854853	11.162403
O	3.717369	8.290321	9.109463
O	10.533601	10.606133	12.126826
O	10.442300	9.971353	5.332685
O	8.576691	11.090630	8.514045
O	9.869937	9.351417	14.523522
O	13.309847	7.531260	7.845107
O	10.341322	15.144796	7.834798
O	10.143235	5.400673	9.279143
O	11.575028	12.733129	7.576755
O	5.228369	7.609424	12.248509
O	6.093026	10.280299	7.558900
O	8.632596	6.131146	12.392790
Pd	11.831340	6.250031	8.475092

Structures at Fig. 3

66

Structure **Pd1a** E = -515.680 eV (E^f = -4.50 eV)

Ce	13.569840	8.344120	10.025640
Ce	12.334620	14.945320	7.374720
Ce	13.996580	14.543540	10.486960
Ce	10.760140	6.343080	11.251180
Ce	5.653980	8.098220	7.959020
Ce	9.009040	7.267380	14.272900
Ce	9.285760	13.264160	8.331380
Ce	9.975640	10.044680	10.020480
Ce	7.203960	7.172120	11.116940
Ce	5.746000	8.667440	14.119320
Ce	11.324260	12.115940	5.470840
Ce	6.522420	11.314800	9.543480
Ce	11.309060	12.774540	12.148360
Ce	9.018020	6.758760	7.960560
Ce	14.674881	11.300800	11.784641
Ce	13.452500	11.612000	8.376380
Ce	12.002800	9.221360	13.295881
Ce	4.089940	9.078120	11.010320
Ce	11.829920	8.768640	6.742260
Ce	8.551620	10.812800	13.352261

Ce	8.361420	10.111620	6.434560
O	14.110900	13.682719	8.289760
O	6.954220	6.464880	8.967800
O	13.051800	15.932940	9.145220
O	9.587380	12.701920	13.441900
O	4.554240	11.044140	10.415400
O	11.085980	6.950040	13.395979
O	7.732820	8.524740	12.905220
O	12.854400	6.173420	10.315740
O	7.514040	7.835560	6.662680
O	9.270960	12.361380	6.158240
O	12.980560	10.734940	6.329180
O	10.964400	6.640800	6.721920
O	14.111340	9.106000	12.073120
O	7.108660	7.291160	15.214520
O	13.138040	12.372000	10.527660
O	12.127800	9.838200	8.854760
O	10.326459	12.384501	10.141220
O	12.984140	11.460021	13.147560
O	9.852260	8.859560	7.787940
O	11.600080	8.422160	11.093740
O	9.169500	7.735060	10.157720
O	6.189700	8.909300	9.997020
O	15.479881	13.240700	11.523941
O	7.244460	13.277760	9.036060
O	12.455180	14.602420	11.921800
O	6.699660	10.515220	14.444500
O	4.038920	9.516200	13.111720
O	14.765080	10.299300	9.694360
O	12.184260	14.046960	5.340520
O	8.016560	10.742900	11.154480
O	3.818180	8.106040	9.017540
O	10.543940	10.557179	12.142320
O	10.539640	9.981060	5.337420
O	8.613720	11.025740	8.516640
O	9.877720	9.247340	14.511960
O	13.438801	7.583540	7.867640
O	10.301580	15.124120	7.888340
O	10.307339	5.355980	9.227280
O	11.594400	12.742360	7.620380
O	5.310520	7.417460	12.162360
O	6.167280	10.164920	7.517620
O	8.742260	6.018380	12.332460
O	8.224980	3.449900	5.736300
Pd	11.986001	6.258320	8.460160
C	8.421640	4.375280	6.369500

66

Structure **Pd1b,** E=-515.558 eV (E^f=-4.37 eV)

Ce	13.572420	8.345800	10.027940
Ce	12.331860	14.945420	7.371500
Ce	13.995680	14.542760	10.485100
Ce	10.772680	6.340420	11.256020
Ce	5.667040	8.079620	7.960440
Ce	9.015760	7.266960	14.276561
Ce	9.284460	13.258580	8.323520
Ce	9.980900	10.050480	10.024199
Ce	7.215500	7.171760	11.119180
Ce	5.749040	8.666320	14.114360
Ce	11.324140	12.111779	5.469040
Ce	6.523420	11.304179	9.535520
Ce	11.312920	12.775140	12.147599
Ce	9.046640	6.767120	7.975340
Ce	14.679400	11.302080	11.786540
Ce	13.454260	11.613580	8.377260
Ce	12.008400	9.220940	13.302000
Ce	4.092720	9.069320	11.004400
Ce	11.840820	8.768700	6.745560
Ce	8.555680	10.813880	13.351740
Ce	8.359160	10.102381	6.427740
O	14.110800	13.683900	8.287900
O	6.979100	6.460720	8.976360
O	13.042880	15.928400	9.144840
O	9.589700	12.702720	13.439920
O	4.555880	11.036220	10.409120
O	11.094800	6.952560	13.401400
O	7.742220	8.524460	12.904580
O	12.868580	6.181280	10.334181
O	7.530820	7.817900	6.673160
O	9.271480	12.357600	6.153380
O	12.985240	10.734740	6.331860
O	10.974400	6.633260	6.723020
O	14.117340	9.107940	12.074720
O	7.112320	7.291420	15.211420
O	13.139220	12.373320	10.528580
O	12.126440	9.841720	8.858340
O	10.328881	12.374940	10.136859
O	12.987500	11.462700	13.147740
O	9.861180	8.848200	7.791900
O	11.602520	8.424440	11.095840
O	9.183800	7.729760	10.149900
O	6.195780	8.904280	9.996060
O	15.480220	13.243400	11.521320
O	7.245000	13.266600	9.026200
O	12.454680	14.602180	11.922100
O	6.703360	10.514700	14.439861
O	4.043020	9.512600	13.107181
O	14.767200	10.303720	9.696740
O	12.180880	14.043800	5.338240

O	8.022880	10.737160	11.149780
O	3.828840	8.089140	9.017100
O	10.548460	10.558000	12.142540
O	10.549960	9.972140	5.341160
O	8.618740	11.016080	8.512600
O	9.881680	9.249260	14.512740
O	13.449380	7.602740	7.871620
O	10.298040	15.119060	7.884900
O	10.320240	5.349660	9.239320
O	11.594120	12.740680	7.618520
O	5.318800	7.415000	12.160980
O	6.166980	10.150120	7.516060
O	8.755360	6.020660	12.334501
O	12.465140	3.293840	6.979380
Pd	12.042120	6.180420	8.434960
C	12.951880	4.154820	7.565580

66

Structure **Pd1c** E=-515.293 eV (E^f=-4.11 eV)

Ce	13.535460	8.269180	9.982020
Ce	12.344160	14.922160	7.384500
Ce	14.008580	14.520360	10.494820
Ce	10.844200	6.324940	11.176280
Ce	5.658060	8.078700	7.977220
Ce	9.074080	7.291800	14.242520
Ce	9.261100	13.280441	8.302140
Ce	9.949100	10.041320	9.957840
Ce	7.241060	7.161840	11.113640
Ce	5.778820	8.645500	14.124680
Ce	11.322480	12.103120	5.478320
Ce	6.496160	11.319340	9.524520
Ce	11.262760	12.812160	12.106060
Ce	9.032480	6.746680	7.961500
Ce	14.627440	11.271100	11.798240
Ce	13.444459	11.576400	8.376800
Ce	11.923240	9.264940	13.164240
Ce	4.108040	9.061760	11.022680
Ce	11.830800	8.738860	6.730680
Ce	8.519020	10.848780	13.319980
Ce	8.349640	10.096540	6.423320
O	14.115160	13.648380	8.311620
O	6.943860	6.451560	8.992100
O	13.069921	15.903360	9.175980
O	9.543840	12.737380	13.401361
O	4.541440	11.032220	10.426980
O	11.171380	7.117680	13.381560
O	7.773400	8.544040	12.904220
O	12.939020	6.316720	10.702159
O	7.495340	7.830880	6.683560
O	9.260560	12.364320	6.151780

O	12.984600	10.746100	6.325760
O	10.839440	6.691920	6.586800
O	13.959360	9.118840	12.148660
O	7.204280	7.290600	15.209900
O	13.115680	12.358980	10.548420
O	12.085741	9.865280	8.843220
O	10.292240	12.414600	10.122060
O	12.861340	11.379220	13.164639
O	9.847840	8.849140	7.800380
O	11.529700	8.490220	11.032720
O	9.153760	7.760460	10.132500
O	6.198400	8.905680	10.009520
O	15.462960	13.185500	11.568460
O	7.220560	13.281020	9.008940
O	12.464679	14.594860	11.938380
O	6.723440	10.502620	14.469740
O	4.086520	9.502220	13.142320
O	14.744040	10.271020	9.757940
O	12.191380	14.045780	5.359480
O	7.986500	10.778360	11.141360
O	3.820000	8.087340	9.056100
O	10.511900	10.581540	12.099380
O	10.530640	9.989120	5.342440
O	8.593040	11.031500	8.494400
O	9.992340	9.322980	14.430660
O	13.365279	7.664360	7.867880
O	10.310320	15.127780	7.888540
O	10.291640	5.445960	9.214500
O	11.588800	12.733400	7.629660
O	5.351960	7.404960	12.182860
O	6.147400	10.156519	7.523780
O	8.827880	6.047800	12.360640
O	14.086061	3.495580	8.868460
Pd	12.130280	5.483420	7.663040
C	13.337120	4.244040	8.397420

65

Structure **Pd1aV** E = -509.008 eV (E^f = -2.71 eV)

Ce	13.717120	8.378140	9.904660
Ce	12.327440	14.964780	7.310400
Ce	13.982821	14.575861	10.444200
Ce	10.469379	6.182980	11.387360
Ce	5.509320	8.237940	8.067140
Ce	8.906040	7.437660	14.400780
Ce	9.252360	13.341700	8.274720
Ce	9.866480	10.122480	9.958280
Ce	7.053220	7.362980	11.256720
Ce	5.678440	8.987580	14.216620

Ce	11.254400	12.110140	5.446920
Ce	6.465720	11.470660	9.540900
Ce	11.366340	12.756000	12.075800
Ce	8.834540	6.838200	8.092000
Ce	14.683220	11.316600	11.765620
Ce	13.386101	11.638860	8.367920
Ce	12.028739	9.109700	13.203760
Ce	3.996720	9.351540	11.096280
Ce	11.724100	8.784520	6.773040
Ce	8.584580	10.891300	13.339860
Ce	8.246460	10.152760	6.433140
O	14.070520	13.697180	8.230700
O	6.789540	6.603700	9.112300
O	13.025060	15.956160	9.072500
O	9.689420	12.720979	13.394980
O	4.501800	11.288420	10.414960
O	10.879520	6.967800	13.603940
O	7.647300	8.717820	12.985420
O	10.768760	6.686560	6.823880
O	7.363960	7.890320	6.767700
O	9.204740	12.392499	6.115240
O	12.889680	10.714360	6.329440
O	14.222960	9.209760	12.081840
O	6.994860	7.632040	15.354320
O	13.117540	12.405500	10.493600
O	12.091579	9.792640	8.876240
O	10.300480	12.391340	10.074880
O	12.944820	11.452880	13.094720
O	9.725980	8.903260	7.835080
O	11.929920	7.869240	11.159880
O	9.091440	7.768420	10.244319
O	6.082080	9.099800	10.068600
O	15.443500	13.305280	11.502481
O	7.227040	13.414381	9.000880
O	12.437160	14.636540	11.863160
O	6.721240	10.793620	14.449440
O	3.994220	9.878900	13.170420
O	14.792680	10.421600	9.612820
O	12.137660	14.024140	5.286100
O	8.000340	10.860519	11.124361
O	3.682320	8.320760	9.134880
O	10.590520	10.426400	11.998020
O	10.442780	9.964320	5.362840
O	8.520360	11.118259	8.475180
O	9.878940	9.397840	14.494081
O	13.271040	7.397340	7.680120
O	10.283240	15.176780	7.802900
O	10.148380	5.297460	9.101700
O	11.533620	12.773319	7.581260
O	5.166400	7.699340	12.287019

O	6.059080	10.274400	7.548300
O	8.430060	6.132080	12.508620
O	13.812020	3.969060	9.627600
Pd	11.944540	5.885580	8.226380
C	13.104700	4.783700	9.200080

66

Structure **Pd2a** E=-516.855 eV (E^f = -5.67 eV)

Ce	13.559375	8.410863	10.133216
Ce	12.379528	14.965034	7.342820
Ce	14.007571	14.628309	10.483727
Ce	10.868003	6.404518	11.516416
Ce	5.694300	8.115974	7.935587
Ce	8.991755	7.450124	14.385432
Ce	9.323576	13.293884	8.290058
Ce	9.999865	10.111706	10.054755
Ce	7.286787	7.232423	11.134359
Ce	5.725906	8.792026	14.089217
Ce	11.390420	12.092576	5.480374
Ce	6.555055	11.345148	9.491081
Ce	11.301811	12.889891	12.141865
Ce	9.010774	6.777698	7.887870
Ce	14.668235	11.405337	11.855861
Ce	13.464082	11.649796	8.434532
Ce	11.995596	9.384148	13.400307
Ce	4.127550	9.106688	10.955374
Ce	11.870337	8.764297	6.844611
Ce	8.530895	10.947230	13.345872
Ce	8.408228	10.131052	6.413306
O	14.135345	13.712249	8.307131
O	6.987632	6.529438	8.957589
O	13.084713	15.986120	9.101683
O	9.544204	12.848055	13.394356
O	4.594823	11.075196	10.371424
O	11.123103	7.106615	13.603714
O	7.754927	8.640776	12.920759
O	12.861887	6.247253	10.498377
O	7.540293	7.840676	6.608692
O	9.325899	12.367265	6.128191
O	13.022132	10.726401	6.408076
O	10.988270	6.629992	6.842561
O	14.096085	9.224987	12.185731
O	7.067425	7.489635	15.277077
O	13.144566	12.457095	10.564644
O	12.097349	9.901568	8.946711
O	10.349915	12.417872	10.123637
O	12.957871	11.608545	13.200395
O	9.845788	8.858802	7.828085
O	11.581904	8.506378	11.220219

O	9.289305	7.499937	10.132568
O	6.248336	8.944106	9.967510
O	15.476719	13.336900	11.564957
O	7.278790	13.303832	8.978142
O	12.443155	14.713243	11.893754
O	6.648015	10.666785	14.375094
O	4.001971	9.549990	13.059827
O	14.754800	10.360082	9.786550
O	12.260840	14.016398	5.327968
O	8.050650	10.773289	11.120472
O	3.854412	8.128088	8.988286
O	10.543895	10.670995	12.176342
O	10.608300	9.958797	5.383579
O	8.664227	11.057639	8.500766
O	9.839528	9.443658	14.574398
O	13.425207	7.694936	8.027516
O	10.342052	15.147697	7.835333
O	9.688006	4.440361	8.330160
O	11.628971	12.770505	7.620319
O	5.378774	7.487109	12.138569
O	6.207294	10.185276	7.488204
O	8.797262	6.165091	12.499981
O	10.713978	4.235518	10.336621
Pd	11.978790	6.140299	8.610364
C	10.607414	4.702490	9.133109

66

Structure **Pd2b** E = -515.835 eV (E^f = 4.65 eV)

Ce	13.555700	8.380120	10.072160
Ce	12.390701	14.950320	7.345020
Ce	14.028220	14.598260	10.477940
Ce	10.859520	6.453640	11.491040
Ce	5.687240	8.135040	7.948020
Ce	8.965280	7.436180	14.365619
Ce	9.324540	13.312200	8.292720
Ce	9.994040	10.126220	10.049220
Ce	7.254540	7.244360	11.139380
Ce	5.709080	8.807620	14.092240
Ce	11.388520	12.087080	5.484740
Ce	6.553800	11.369200	9.494040
Ce	11.315540	12.886200	12.143600
Ce	9.022840	6.846220	7.921840
Ce	14.670640	11.367480	11.833940
Ce	13.471320	11.630820	8.421480
Ce	11.999600	9.385580	13.381020
Ce	4.109020	9.129520	10.963980
Ce	11.900200	8.744820	6.855580
Ce	8.534760	10.951080	13.346061
Ce	8.413400	10.151660	6.410800

O	14.147100	13.697881	8.301440
O	6.978240	6.513360	8.979340
O	13.096600	15.970320	9.106620
O	9.553300	12.840600	13.407919
O	4.590160	11.092180	10.379840
O	11.102240	7.096960	13.572741
O	7.739940	8.636840	12.917080
O	12.797101	6.192980	10.499680
O	7.546600	7.859200	6.635120
O	9.317820	12.381420	6.132740
O	13.011360	10.741600	6.396120
O	10.915560	6.587140	6.865400
O	14.090480	9.208640	12.153600
O	7.047780	7.476040	15.264320
O	13.140140	12.441640	10.557960
O	12.074701	9.907260	8.949080
O	10.337480	12.435360	10.133760
O	12.962840	11.589880	13.196100
O	9.837920	8.881260	7.833640
O	11.579640	8.508900	11.214120
O	9.256200	7.562060	10.150880
O	6.234180	8.954520	9.971580
O	15.487640	13.307320	11.543241
O	7.269840	13.324660	8.982540
O	12.462640	14.700400	11.900860
O	6.651860	10.666320	14.384880
O	3.994220	9.588920	13.068340
O	14.757860	10.359740	9.774840
O	12.264720	14.016200	5.333460
O	8.037800	10.788940	11.126360
O	3.843120	8.145440	8.992780
O	10.541240	10.672340	12.173541
O	10.601860	9.962080	5.390540
O	8.652300	11.076900	8.500460
O	9.830580	9.418060	14.564140
O	13.614780	7.928620	7.929840
O	10.344000	15.161361	7.838960
O	9.271120	3.676460	8.752800
O	11.623740	12.772660	7.629740
O	5.338840	7.507120	12.145920
O	6.196060	10.197120	7.492020
O	8.762940	6.130560	12.467260
O	10.512160	3.565920	10.751160
Pd	11.920480	6.197560	8.630820
C	9.928740	3.651700	9.728180

65

Structure **Pd2aV** E= -509.603 eV (E^f = -3.30 eV)

Ce	13.512760	8.364320	10.113860

Ce	12.357579	14.967520	7.381760
Ce	14.035480	14.603500	10.514400
Ce	10.810961	6.302720	11.601740
Ce	5.584480	8.108700	8.073060
Ce	8.972720	7.556160	14.466560
Ce	9.310480	13.240660	8.286480
Ce	9.954360	9.962720	10.073940
Ce	7.195100	7.248380	11.288821
Ce	5.706360	8.915580	14.209740
Ce	11.357700	12.136700	5.476120
Ce	6.544020	11.325620	9.500620
Ce	11.247280	12.927320	12.100520
Ce	8.774440	6.554720	8.068680
Ce	14.622860	11.352060	11.893580
Ce	13.471240	11.659380	8.460380
Ce	11.883579	9.412800	13.314660
Ce	4.093620	9.222160	11.084501
Ce	11.978700	8.874300	6.798060
Ce	8.479620	11.057640	13.341841
Ce	8.393280	9.962920	6.449760
O	14.158300	13.702260	8.343620
O	6.760280	6.455340	9.184260
O	13.088380	15.963420	9.154360
O	9.535000	12.937360	13.384440
O	4.568580	11.139280	10.402100
O	11.131279	7.285860	13.702980
O	7.732660	8.736480	12.999320
O	12.999980	6.259260	10.507960
O	7.361560	7.750200	6.744880
O	9.322000	12.308980	6.105360
O	13.082401	10.772440	6.397040
O	13.977040	9.206760	12.247820
O	7.081180	7.639840	15.405920
O	13.128400	12.428960	10.585420
O	12.122680	9.853220	8.865700
O	10.294780	12.570300	10.150700
O	12.878560	11.506400	13.233000
O	9.976020	8.444280	7.554700
O	11.589320	8.483360	11.252880
O	9.191680	7.444240	10.202841
O	6.211280	8.970040	10.077840
O	15.469860	13.251960	11.615900
O	7.268700	13.277620	8.902020
O	12.502960	14.708400	11.927680
O	6.657660	10.773860	14.477921
O	4.003560	9.714180	13.189501
O	14.725339	10.305760	9.825800
O	12.216780	14.065620	5.344700
O	7.925700	10.937800	11.200880
O	3.752420	8.214100	9.149820

O	10.516840	10.697680	12.205180
O	10.671480	9.962100	5.371860
O	8.670780	10.936580	8.441540
O	9.924780	9.562940	14.554120
O	13.334939	7.586000	7.910940
O	10.336220	15.131561	7.828120
O	10.441540	4.495020	7.866260
O	11.639040	12.752500	7.626440
O	5.309600	7.587080	12.309420
O	6.179140	10.140460	7.530860
O	8.681080	6.202480	12.708220
O	10.418620	4.086920	10.150959
Pd	12.223279	5.843840	8.651920
C	10.872340	4.533800	9.082020

65

Structure **Pd2bV** E = -509.039 eV ((E^f = -2.74 eV)

Ce	13.583781	8.383360	10.315520
Ce	12.368400	14.891440	7.337760
Ce	14.014380	14.558460	10.440240
Ce	10.896660	6.402680	11.583260
Ce	5.675240	8.102400	8.042280
Ce	8.988460	7.521060	14.451360
Ce	9.265500	13.302400	8.294880
Ce	9.982540	10.052299	10.012500
Ce	7.283260	7.251640	11.232520
Ce	5.720180	8.853540	14.174600
Ce	11.339080	12.061700	5.479700
Ce	6.517540	11.389400	9.524500
Ce	11.231180	12.940440	12.101340
Ce	9.001000	6.809420	7.971680
Ce	14.625160	11.399980	11.913860
Ce	13.436840	11.529400	8.407160
Ce	11.868740	9.483380	13.393580
Ce	4.121800	9.129240	11.046619
Ce	11.771760	8.682240	6.659260
Ce	8.467200	11.050940	13.337660
Ce	8.330500	10.144759	6.421520
O	14.120560	13.595060	8.305780
O	6.980600	6.489120	9.086760
O	13.159000	15.942000	9.068520
O	9.509180	12.936400	13.391380
O	4.537940	11.074179	10.402000
O	11.155339	7.302700	13.713560
O	7.768540	8.703580	12.989420
O	10.873719	6.409860	6.951940
O	7.491380	7.795920	6.700000
O	9.282260	12.375640	6.134380
O	13.026100	10.737700	6.353840

O	14.001260	9.235900	12.360860
O	7.102700	7.570160	15.378280
O	13.119000	12.364700	10.577700
O	12.306360	9.609560	8.844280
O	10.290740	12.577720	10.155900
O	12.857341	11.549079	13.266540
O	9.788200	8.846360	7.765820
O	11.630120	8.543580	11.333480
O	9.285860	7.496840	10.191340
O	6.254520	8.950820	10.051060
O	15.468100	13.281120	11.605020
O	7.213260	13.347160	8.970200
O	12.488379	14.701281	11.866120
O	6.634520	10.716300	14.464400
O	3.998680	9.607160	13.156240
O	14.785579	10.277760	9.898180
O	12.221100	14.003260	5.331580
O	7.914220	10.933520	11.212640
O	3.835180	8.105220	9.112860
O	10.505800	10.709500	12.218800
O	10.497180	9.989880	5.286160
O	8.577140	11.092740	8.486400
O	9.915820	9.559960	14.583941
O	13.065640	6.304820	10.412660
O	10.351640	15.139920	7.819900
O	9.577720	3.715840	8.603300
O	11.599640	12.688040	7.644400
O	5.379500	7.528040	12.257500
O	6.135760	10.151780	7.532780
O	8.749020	6.178360	12.648320
O	11.255100	3.093440	10.134521
Pd	12.051080	6.200620	8.622940
C	10.439520	3.417680	9.351080

66

Structure **Pd3a** E = -517.107 eV (E^f = -5.92 eV)

Ce	13.481441	8.081320	10.073560
Ce	12.516260	14.759080	7.445360
Ce	14.096280	14.286560	10.589660
Ce	10.301260	6.089240	11.687080
Ce	5.474100	8.327540	7.919440
Ce	8.574120	7.496760	14.441900
Ce	9.352920	13.300160	8.401420
Ce	9.868020	9.857500	10.020740
Ce	6.894600	7.316040	11.126820
Ce	5.418460	9.037100	14.049380
Ce	11.316460	12.001520	5.541120
Ce	6.489180	11.520240	9.543180
Ce	11.267819	12.709860	12.164540

Ce	8.712760	6.731160	7.887500
Ce	14.533600	11.020080	11.948280
Ce	13.394660	11.356480	8.492680
Ce	11.716120	9.109840	13.272740
Ce	3.881720	9.416240	10.894541
Ce	11.700220	8.605960	6.764600
Ce	8.376600	10.971040	13.323700
Ce	8.317640	10.173520	6.551240
O	14.176680	13.381341	8.398620
O	6.522680	6.631720	8.950000
O	13.273780	15.718960	9.208020
O	9.529260	12.769001	13.421280
O	4.463900	11.341020	10.310180
O	10.693660	7.057780	13.725320
O	7.461300	8.719740	12.912860
O	13.338240	7.445960	7.662100
O	7.322300	8.061160	6.599040
O	9.226580	12.287120	6.205940
O	12.876540	10.553360	6.384660
O	10.821840	6.518060	6.784240
O	13.820140	8.925200	12.320360
O	6.665080	7.695560	15.311019
O	13.087519	12.161980	10.636480
O	12.044120	9.606420	8.909740
O	10.289561	12.484560	10.211740
O	12.753880	11.204540	13.274980
O	9.659420	8.805380	7.774340
O	11.479020	8.028420	11.268200
O	8.940020	7.381820	10.164061
O	6.034140	9.123220	9.951400
O	15.445960	12.911080	11.711620
O	7.307700	13.439000	9.035140
O	12.551121	14.461941	11.989940
O	6.467760	10.814720	14.367720
O	3.756840	9.890900	12.991461
O	14.683760	10.074860	9.867800
O	12.290440	13.857340	5.399900
O	7.828480	10.939140	11.194280
O	3.605300	8.486560	8.900520
O	10.432540	10.455260	12.175120
O	10.339900	9.929880	5.407880
O	8.564800	11.079980	8.514900
O	9.672960	9.405620	14.525300
O	10.498300	15.082840	7.892380
O	11.163419	4.546140	9.476000
O	11.608620	12.603860	7.688560
O	4.999900	7.725680	12.101880
O	6.199900	10.387861	7.444620
O	8.210900	6.127040	12.628421
O	12.095020	4.219320	11.507040

O	13.233221	5.288620	9.846440
Pd	12.140100	5.920660	8.200420
C	12.196780	4.606600	10.318420

66

Structure **Pd3b** E=-516.371 eV (E^f = -5.19 eV)

Ce	13.569740	8.235600	10.096120
Ce	12.463599	14.781060	7.346140
Ce	14.058360	14.402961	10.512460
Ce	10.375839	6.166580	11.731000
Ce	5.585640	8.169480	7.995580
Ce	8.672780	7.624700	14.528620
Ce	9.333580	13.278880	8.352180
Ce	9.914120	9.930500	10.074120
Ce	6.982740	7.339440	11.268840
Ce	5.474140	9.109860	14.109600
Ce	11.326460	11.931680	5.525480
Ce	6.499360	11.457080	9.524460
Ce	11.274199	12.809600	12.137361
Ce	8.900480	6.701560	8.144060
Ce	14.571919	11.176380	11.946940
Ce	13.430520	11.442840	8.468860
Ce	11.786320	9.227500	13.306820
Ce	3.925820	9.340560	10.922821
Ce	11.787640	8.575320	6.876820
Ce	8.400600	11.068100	13.337981
Ce	8.382740	10.057000	6.593040
O	14.169559	13.469000	8.325200
O	6.777020	6.553760	9.069640
O	13.179719	15.775200	9.104780
O	9.535180	12.878680	13.390300
O	4.462500	11.251960	10.259060
O	10.798140	7.203040	13.820740
O	7.510460	8.805720	12.983960
O	11.433080	4.148780	10.922720
O	7.490600	7.886120	6.757900
O	9.233660	12.183321	6.183000
O	12.909180	10.560880	6.398160
O	10.737020	6.519960	6.969500
O	13.911560	9.087440	12.365479
O	6.742700	7.822300	15.390160
O	13.098940	12.267621	10.600801
O	12.099220	9.683580	8.951700
O	10.283020	12.521560	10.188860
O	12.788460	11.356220	13.263119
O	9.744760	8.777060	7.904560
O	11.593060	8.115460	11.318260
O	9.005660	7.585600	10.294420
O	6.080620	9.059440	10.003279
O	15.438060	13.081980	11.667200

O	7.278040	13.378640	8.971880
O	12.520660	14.578000	11.921020
O	6.491220	10.915540	14.383060
O	3.813320	9.913940	12.987260
O	14.743980	10.207100	9.872720
O	12.255820	13.807480	5.333460
O	7.839500	10.978420	11.202199
O	3.715680	8.295440	8.950320
O	10.467240	10.545640	12.211120
O	10.391620	9.821020	5.482160
O	8.579860	11.044919	8.519040
O	9.716320	9.547920	14.573280
O	13.500400	7.584420	7.785440
O	10.433880	15.067260	7.789980
O	9.892720	4.939340	9.504500
O	11.605080	12.616380	7.657620
O	5.064600	7.727740	12.199180
O	6.251780	10.218680	7.443920
O	8.291660	6.225500	12.757260
O	11.791900	3.958500	8.693660
Pd	12.487720	5.809360	8.021040
C	11.071579	4.317560	9.694360

66

Structure **Pd3c** E= -516.239 eV ($E^{f} =$ -5.05 eV)

Ce	13.637720	8.519360	10.184840
Ce	12.330060	14.896719	7.194220
Ce	13.914580	14.678160	10.374980
Ce	10.579420	6.357980	11.700300
Ce	5.661820	8.136520	8.070080
Ce	8.843900	7.605180	14.544740
Ce	9.290820	13.292420	8.286360
Ce	9.934740	10.073960	10.091200
Ce	7.134280	7.380380	11.273720
Ce	5.611640	9.049860	14.173959
Ce	11.285720	11.924380	5.486980
Ce	6.506420	11.429020	9.551560
Ce	11.289740	12.907020	12.095660
Ce	9.051140	6.659600	8.086460
Ce	14.642780	11.520040	11.877980
Ce	13.371700	11.624520	8.428320
Ce	11.973500	9.377380	13.449301
Ce	4.013420	9.282640	11.023180
Ce	11.796000	8.555260	6.917020
Ce	8.508620	11.030480	13.377920
Ce	8.397060	10.022960	6.610800
O	14.053960	13.676600	8.218220
O	6.826240	6.587020	9.174160
O	12.991780	15.969160	8.924280
O	9.576700	12.885799	13.381720

O	4.519960	11.225680	10.351260
O	10.907741	7.171120	13.772640
O	7.641320	8.790960	13.044820
O	11.159101	4.128380	11.442879
O	7.499700	7.940580	6.761780
O	9.183280	12.178220	6.184280
O	12.895300	10.615520	6.433000
O	10.816340	6.794720	6.547480
O	14.147340	9.361640	12.243180
O	6.917040	7.750240	15.418260
O	13.105299	12.494280	10.537920
O	12.100680	9.861000	9.105340
O	10.307339	12.427860	10.117280
O	12.914140	11.676760	13.199160
O	9.812340	8.874840	7.989120
O	11.829540	8.187340	11.407140
O	9.192820	7.730060	10.401061
O	6.152680	9.079540	10.063640
O	15.391459	13.468940	11.533240
O	7.263860	13.360440	8.987360
O	12.344820	14.773980	11.764600
O	6.602320	10.889540	14.402840
O	3.921900	9.847840	13.090720
O	14.755200	10.436500	9.789900
O	12.152400	13.832420	5.232440
O	8.006880	10.872120	11.171980
O	3.791400	8.243560	9.075900
O	10.555040	10.592360	12.152100
O	10.392140	9.856260	5.491360
O	8.607280	11.054980	8.557580
O	9.791980	9.570560	14.641840
O	13.478240	7.552160	8.260360
O	10.290740	15.104440	7.682020
O	10.400060	5.086800	9.565660
O	11.541460	12.714460	7.588160
O	5.216860	7.706880	12.272660
O	6.276580	10.230020	7.524020
O	8.565600	6.250380	12.666759
O	10.849100	2.829900	9.584380
Pd	12.070040	6.199480	8.877860
C	10.837380	3.878320	10.181479

65

Structure **Pd3aV** E = -509.937 (E^f = -3.64 eV)

Ce	13.452480	8.004720	10.283620
Ce	12.539500	14.708420	7.438200
Ce	14.104580	14.237820	10.564280
Ce	10.438960	6.128900	11.647440
Ce	5.454320	8.353300	7.906800
Ce	8.613860	7.472060	14.419000

Ce	9.351320	13.306880	8.407300
Ce	9.915520	9.894100	10.023700
Ce	6.963900	7.319640	11.126020
Ce	5.441360	8.987100	14.052700
Ce	11.324641	11.985520	5.541380
Ce	6.489500	11.525840	9.554960
Ce	11.249460	12.727140	12.172860
Ce	8.681300	6.825300	7.858500
Ce	14.519240	11.011240	11.965380
Ce	13.388580	11.271280	8.485400
Ce	11.680640	9.175800	13.342240
Ce	3.899980	9.388340	10.909140
Ce	11.594180	8.563460	6.653020
Ce	8.361460	10.989940	13.330719
Ce	8.287000	10.246620	6.529560
O	14.176420	13.302900	8.394180
O	6.618260	6.640660	8.881600
O	13.322980	15.684021	9.176280
O	9.493340	12.804680	13.416359
O	4.458480	11.312160	10.328540
O	10.722179	7.080840	13.740840
O	7.490920	8.712460	12.907220
O	13.378760	5.567620	9.348480
O	7.304080	8.081480	6.592660
O	9.206960	12.316360	6.222260
O	12.887700	10.570000	6.336840
O	10.358620	6.408880	6.652500
O	13.782780	8.935520	12.418740
O	6.694140	7.649920	15.293940
O	13.072720	12.111700	10.635020
O	12.135201	9.441340	8.826460
O	10.291040	12.448560	10.203140
O	12.732440	11.249460	13.298120
O	9.593360	8.839500	7.772780
O	11.392519	8.209840	11.300679
O	8.981640	7.370260	10.104340
O	6.043380	9.104160	9.931580
O	15.447080	12.896800	11.709100
O	7.303520	13.445280	9.068060
O	12.549520	14.453800	11.954240
O	6.446560	10.787940	14.365420
O	3.747680	9.819160	13.001920
O	14.682740	10.014300	9.931380
O	12.301580	13.845220	5.400200
O	7.811000	10.930080	11.199460
O	3.607780	8.447200	8.902000
O	10.393560	10.517180	12.211940
O	10.245740	9.975140	5.355720
O	8.544940	11.092460	8.517840
O	9.662860	9.420660	14.551719

O	10.520680	15.064180	7.905640
O	11.750200	4.364300	10.439380
O	11.600580	12.558100	7.694220
O	5.057540	7.697600	12.089800
O	6.163120	10.409480	7.435560
O	8.275920	6.137280	12.586319
O	13.066280	5.804400	11.549040
Pd	11.851240	5.916220	7.941100
C	12.748100	5.171760	10.436380

65

Structure **Pd3bV** E = -509.549 (E^f = -3.25 eV)

Ce	13.669720	8.523980	10.166460
Ce	12.315420	14.928240	7.231260
Ce	13.921340	14.694560	10.412101
Ce	10.518380	6.359020	11.710740
Ce	5.596000	8.183320	8.064540
Ce	8.787580	7.639420	14.554000
Ce	9.314380	13.253500	8.302100
Ce	10.007520	9.968300	10.097460
Ce	7.021580	7.352140	11.331240
Ce	5.564460	9.099140	14.196180
Ce	11.313241	11.973320	5.476960
Ce	6.533740	11.418860	9.546820
Ce	11.262540	12.918460	12.105800
Ce	8.861740	6.622720	8.264360
Ce	14.637541	11.523499	11.880120
Ce	13.466660	11.680160	8.410500
Ce	11.944780	9.388060	13.424560
Ce	3.977780	9.347840	11.021841
Ce	12.012440	8.711860	6.911700
Ce	8.480980	11.077040	13.362240
Ce	8.470020	9.959340	6.607400
O	14.097840	13.738340	8.235040
O	6.743520	6.546100	9.216220
O	12.942320	15.977319	8.990000
O	9.553580	12.924500	13.406860
O	4.502440	11.243740	10.318700
O	10.875380	7.164680	13.719960
O	7.584120	8.793620	13.036420
O	11.193500	4.170520	11.347400
O	7.461420	7.865260	6.805060
O	9.222980	12.134560	6.183340
O	13.033240	10.733280	6.383400
O	14.108820	9.320100	12.204580
O	6.869420	7.783440	15.435780
O	13.113580	12.490320	10.525980
O	12.216340	9.852600	8.994140
O	10.283840	12.567900	10.156400

O	12.939160	11.655680	13.202320
O	9.888400	8.624580	7.806500
O	11.760020	8.187100	11.319619
O	9.089100	7.622100	10.382760
O	6.116580	9.062120	10.082721
O	15.409080	13.457640	11.536959
O	7.265240	13.324460	8.927200
O	12.405640	14.762540	11.843760
O	6.586580	10.899060	14.446260
O	3.903200	9.917920	13.091840
O	14.806560	10.437080	9.792000
O	12.132280	13.896880	5.240980
O	7.900420	10.979520	11.226979
O	3.734080	8.309080	9.052520
O	10.540940	10.640841	12.224200
O	10.478860	9.869980	5.442900
O	8.646480	10.963800	8.521140
O	9.755800	9.550720	14.657140
O	13.551320	7.447920	8.289940
O	10.283540	15.097980	7.681240
O	10.381700	5.050980	9.453440
O	11.590880	12.717800	7.599980
O	5.126840	7.719180	12.301040
O	6.288760	10.193959	7.506120
O	8.508000	6.236900	12.688720
O	10.944700	2.823080	9.516020
Pd	12.045760	6.164900	8.795220
C	10.879740	3.884100	10.106980

Structures at Fig. 4

66

Structure **Ag1a** E = -512.945 eV (E^f = -3.07 eV)

Ce	13.538110	8.267855	9.975679
Ce	12.370509	14.900661	7.374157
Ce	14.029242	14.491572	10.485835
Ce	10.787239	6.312901	11.179490
Ce	5.676737	8.085738	7.975729
Ce	9.056804	7.292733	14.257408
Ce	9.277451	13.278809	8.306845
Ce	9.950734	10.037774	9.963017
Ce	7.231280	7.161654	11.113701
Ce	5.774223	8.664985	14.118663
Ce	11.329346	12.081574	5.479293
Ce	6.506090	11.330330	9.524952
Ce	11.289589	12.790323	12.110279
Ce	9.058623	6.745903	7.952743
Ce	14.646748	11.242796	11.797228
Ce	13.442045	11.545832	8.373849

Ce	11.929464	9.236650	13.172832
Ce	4.110104	9.072167	11.013000
Ce	11.804104	8.695713	6.727446
Ce	8.531294	10.849425	13.321196
Ce	8.351019	10.101045	6.423579
O	14.132431	13.614706	8.312008
O	6.947699	6.461877	8.992946
O	13.106924	15.884522	9.165662
O	9.573103	12.728026	13.407679
O	4.549040	11.044582	10.418959
O	11.163032	7.089444	13.389908
O	7.770401	8.548657	12.910041
O	12.871073	6.225789	10.459418
O	7.498333	7.841853	6.679692
O	9.274274	12.370560	6.157790
O	12.979530	10.712868	6.329949
O	10.813824	6.735023	6.622949
O	13.977389	9.077187	12.148641
O	7.189792	7.310457	15.223294
O	13.133560	12.329488	10.551602
O	12.095109	9.834612	8.865296
O	10.311158	12.409199	10.131153
O	12.880747	11.346787	13.168546
O	9.844619	8.866477	7.825150
O	11.555588	8.450050	11.060225
O	9.156756	7.773001	10.156684
O	6.205835	8.914582	10.013868
O	15.478067	13.154218	11.576976
O	7.236993	13.293319	9.007078
O	12.491742	14.575166	11.933209
O	6.727096	10.519662	14.461291
O	4.085781	9.520050	13.133072
O	14.759824	10.249149	9.753112
O	12.226510	14.016486	5.356576
O	7.996373	10.789086	11.148314
O	3.829433	8.094479	9.052809
O	10.527177	10.560124	12.104737
O	10.522531	9.982604	5.336002
O	8.601200	11.042525	8.503272
O	9.998425	9.311113	14.438171
O	13.407105	7.657346	7.883446
O	10.342203	15.122033	7.882299
O	10.201963	5.414815	9.303442
O	11.602075	12.717510	7.630771
O	5.344405	7.414496	12.182761
O	6.155131	10.170469	7.528428
O	8.794371	6.043670	12.383919
O	13.030418	2.663369	6.870400
Ag	12.484649	5.493351	8.146961

65
Structure **Ag1aV** E = -506.218 eV (E^f = -1.23 eV)

Ce	13.685011	8.453608	9.826648
Ce	12.267054	14.978310	7.330579
Ce	13.945863	14.577595	10.442783
Ce	10.553550	6.078572	11.231673
Ce	5.605255	8.114803	7.983847
Ce	8.990811	7.240471	14.291379
Ce	9.208635	13.300428	8.304366
Ce	9.869331	10.068613	9.926851
Ce	7.153625	7.199502	11.132033
Ce	5.745741	8.717222	14.146311
Ce	11.228725	12.119821	5.447378
Ce	6.457322	11.341703	9.534352
Ce	11.359329	12.677755	12.076662
Ce	8.995134	6.781462	7.944791
Ce	14.706213	11.331511	11.718919
Ce	13.380843	11.662300	8.310161
Ce	12.061046	9.004120	13.156321
Ce	4.058403	9.113565	11.042380
Ce	11.751913	8.732291	6.722617
Ce	8.613622	10.719448	13.326581
Ce	8.276259	10.120722	6.399345
O	14.043187	13.731185	8.225355
O	6.908531	6.497036	8.986407
O	12.987217	15.967511	9.110751
O	9.704095	12.572008	13.408369
O	4.509598	11.087726	10.414002
O	10.992210	6.872619	13.478769
O	7.717354	8.529920	12.912931
O	7.442468	7.870588	6.679015
O	9.185028	12.397661	6.130120
O	12.894363	10.737877	6.310830
O	10.728226	6.807240	6.442077
O	14.223959	9.153093	11.918221
O	7.087134	7.356953	15.261008
O	13.142000	12.386804	10.472571
O	12.108943	9.842281	8.907635
O	10.284834	12.365133	10.102015
O	13.010982	11.372762	13.066051
O	9.805155	8.888897	7.811461
O	12.042384	7.795808	11.039057
O	9.171900	7.721578	10.190877
O	6.147251	8.952190	10.009387
O	15.454398	13.299339	11.486999
O	7.189546	13.315521	9.039910
O	12.418306	14.570678	11.879822
O	6.772097	10.535338	14.448591
O	4.055761	9.605009	13.137145
O	14.799279	10.388097	9.539534

O	12.101213	14.061922	5.313838
O	8.013424	10.748148	11.121085
O	3.772821	8.122639	9.069813
O	10.641847	10.325608	11.969502
O	10.456439	10.006830	5.330980
O	8.527737	11.068106	8.466110
O	9.939952	9.222901	14.441350
O	13.283938	7.551275	7.923144
O	10.232882	15.155770	7.864101
O	10.290009	5.395498	9.014785
O	11.514789	12.773222	7.593064
O	5.261054	7.472059	12.189368
O	6.089380	10.192697	7.522705
O	8.541426	5.962412	12.392793
O	13.774955	2.765507	7.062508
Ag	11.984330	5.288901	7.504796
C	13.182687	3.745766	7.169175
C	12.942375	3.697490	7.361389

66

Structure **Ag2a** E = -513.524 eV (E^f = -3.65 eV)

Ce	13.568827	8.446339	10.138514
Ce	12.384492	14.955493	7.321326
Ce	13.991650	14.653546	10.475171
Ce	10.862635	6.439385	11.575840
Ce	5.690135	8.123986	7.942045
Ce	8.954251	7.483619	14.392517
Ce	9.311535	13.323821	8.258983
Ce	10.012119	10.194337	10.045273
Ce	7.267350	7.210137	11.104152
Ce	5.703381	8.805705	14.058491
Ce	11.386223	12.069963	5.486915
Ce	6.552925	11.365471	9.481109
Ce	11.295792	12.930869	12.146755
Ce	8.999094	6.708968	7.815695
Ce	14.658439	11.453530	11.873946
Ce	13.474672	11.654357	8.433381
Ce	11.980042	9.429221	13.411443
Ce	4.119883	9.124069	10.942222
Ce	11.875236	8.745203	6.871199
Ce	8.518730	10.975832	13.334019
Ce	8.410637	10.108004	6.434402
O	14.138687	13.719752	8.305068
O	6.851113	6.495330	9.010553
O	13.084954	16.003410	9.080732
O	9.533773	12.875459	13.392611
O	4.604743	11.097738	10.384190
O	11.116954	7.146002	13.634018
O	7.759148	8.641854	12.898196

O	12.770169	6.161512	10.547369
O	7.459271	7.877348	6.588091
O	9.324756	12.374712	6.121296
O	13.014988	10.742733	6.418650
O	11.114970	6.621538	6.784539
O	14.067796	9.260371	12.192168
O	7.032359	7.516673	15.275328
O	13.136058	12.487661	10.575459
O	12.083663	9.942548	8.991900
O	10.340707	12.463690	10.121671
O	12.954159	11.654115	13.223338
O	9.857243	8.839827	7.858731
O	11.550505	8.497551	11.228290
O	9.331220	7.223967	10.117584
O	6.266382	8.959073	9.975669
O	15.465006	13.388435	11.575994
O	7.275975	13.333608	8.951322
O	12.416966	14.757998	11.883384
O	6.627187	10.688576	14.330169
O	3.968767	9.548548	13.060536
O	14.762785	10.409425	9.826688
O	12.269033	14.005349	5.324399
O	8.061869	10.801847	11.113772
O	3.826088	8.175044	8.996554
O	10.553713	10.710177	12.174615
O	10.614657	9.947783	5.397103
O	8.671354	11.088246	8.494664
O	9.816420	9.479041	14.574643
O	13.621352	7.950321	8.061522
O	10.340410	15.171407	7.796872
O	9.287050	3.500920	8.662252
O	11.626999	12.778988	7.628179
O	5.369614	7.489589	12.130527
O	6.210380	10.222363	7.511122
O	8.788909	6.133623	12.548932
O	10.394443	3.524213	10.738572
Ag	12.188170	5.969971	8.505887
C	9.845575	3.505876	9.695733

66

Structure **Ag2b** E = -513.362 eV (E^f = -3.49 eV)

Ce	13.536211	8.399993	10.146611
Ce	12.381524	14.960502	7.352303
Ce	14.025809	14.613832	10.487322
Ce	10.892351	6.325181	11.608163
Ce	5.662507	8.131909	7.971495
Ce	8.965539	7.479315	14.416654
Ce	9.312235	13.305176	8.296435
Ce	10.005370	10.143799	10.079361
Ce	7.291524	7.236738	11.183771

Ce	5.717487	8.834646	14.115365
Ce	11.383343	12.098858	5.490312
Ce	6.549455	11.353279	9.505922
Ce	11.310955	12.908633	12.157512
Ce	8.956543	6.811369	7.916000
Ce	14.656259	11.390527	11.871527
Ce	13.457776	11.642715	8.445933
Ce	11.965517	9.380475	13.410872
Ce	4.117728	9.113639	10.997391
Ce	11.859754	8.775273	6.850388
Ce	8.522638	10.980568	13.361416
Ce	8.391960	10.144076	6.432133
O	14.138507	13.702537	8.307011
O	6.956553	6.524410	8.965899
O	13.095834	15.981776	9.098965
O	9.552768	12.880890	13.401084
O	4.598103	11.080536	10.390790
O	11.083266	7.141480	13.720225
O	7.742934	8.679941	12.931045
O	12.955643	6.247725	10.435803
O	7.521061	7.853563	6.628795
O	9.315748	12.372969	6.128213
O	13.002096	10.729403	6.406128
O	10.914181	6.573153	6.880345
O	14.091603	9.230719	12.203557
O	7.023786	7.550346	15.314798
O	13.138616	12.457951	10.568932
O	12.071279	9.909299	8.953331
O	10.345076	12.433474	10.129645
O	12.952473	11.625106	13.209369
O	9.824011	8.848364	7.857795
O	11.578563	8.492646	11.236494
O	9.298826	7.364687	10.111517
O	6.244953	8.944812	9.984089
O	15.479436	13.325626	11.565326
O	7.269153	13.316238	8.978887
O	12.465976	14.723853	11.897054
O	6.632919	10.715637	14.380203
O	3.975261	9.584390	13.078973
O	14.737120	10.357274	9.797977
O	12.256137	14.016714	5.329736
O	8.048671	10.789590	11.123608
O	3.830948	8.133051	9.020632
O	10.536507	10.695044	12.185776
O	10.588365	9.947532	5.406423
O	8.660280	11.067752	8.503750
O	9.804462	9.498315	14.587994
O	13.447798	7.788471	7.962322
O	10.339392	15.155464	7.837315
O	9.682693	4.295798	8.505921

O	11.620653	12.772675	7.626095
O	5.364612	7.515104	12.172325
O	6.194851	10.195776	7.505318
O	8.673434	6.152868	12.590481
O	11.257104	3.661009	10.094565
Ag	12.131932	5.950481	8.506523
C	10.694585	4.233368	9.187419

65

Structure **Ag2aV** E = -506.574 eV (E^f = -1.58 eV)

Ce	13.794620	8.365100	9.819960
Ce	12.296860	14.954960	7.345120
Ce	13.962380	14.557360	10.457500
Ce	10.589660	6.189600	11.521140
Ce	5.613740	8.110060	7.960680
Ce	8.925360	7.328620	14.422539
Ce	9.239400	13.289040	8.302940
Ce	9.922940	10.061880	9.922420
Ce	7.182000	7.238060	11.134940
Ce	5.689480	8.779100	14.134440
Ce	11.256620	12.108400	5.458560
Ce	6.486680	11.332620	9.534360
Ce	11.367500	12.690001	12.088580
Ce	8.992380	6.781380	7.897040
Ce	14.708360	11.306961	11.746221
Ce	13.391840	11.621400	8.349440
Ce	12.114760	9.096560	13.196400
Ce	4.049300	9.108400	11.008340
Ce	11.771380	8.720720	6.716340
Ce	8.597980	10.746779	13.312680
Ce	8.297160	10.117739	6.400540
O	14.050740	13.697640	8.251100
O	6.952320	6.515900	8.987920
O	12.999320	15.941540	9.119820
O	9.669300	12.580880	13.403540
O	4.523180	11.070580	10.416340
O	10.977139	6.881580	13.564900
O	7.708820	8.534860	12.961460
O	7.436420	7.849460	6.655220
O	9.198920	12.380780	6.137720
O	12.890360	10.746140	6.320220
O	10.633121	6.746940	6.585920
O	14.227980	9.193640	12.052540
O	7.020220	7.439120	15.327240
O	13.125941	12.374160	10.500979
O	12.109040	9.779720	8.903680
O	10.298600	12.323641	10.105460
O	12.959000	11.397380	13.107860
O	9.773900	8.867540	7.849520

O	11.984580	7.744980	11.143360
O	9.242240	7.606740	10.240901
O	6.155440	8.936840	9.998560
O	15.445300	13.295760	11.512120
O	7.208920	13.294981	9.066600
O	12.409660	14.569960	11.887980
O	6.706700	10.591620	14.390020
O	3.982320	9.581920	13.115860
O	14.837540	10.430100	9.598620
O	12.129580	14.042940	5.326620
O	8.025720	10.691780	11.115299
O	3.779320	8.116780	9.038460
O	10.627880	10.314060	12.002240
O	10.460680	9.981660	5.341720
O	8.550320	11.057520	8.472780
O	9.894420	9.254000	14.511300
O	13.483500	7.673560	7.563440
O	10.248220	15.136020	7.869040
O	10.184300	5.061020	9.246840
O	11.523660	12.756140	7.607480
O	5.288620	7.492200	12.176760
O	6.101500	10.182440	7.522380
O	8.610800	6.011000	12.481641
O	11.828580	4.356660	10.529040
Ag	12.682920	5.720560	8.004100
C	11.463420	4.698740	9.329420

65

Structure **Ag2bV** E = -506.536 eV (E^f = -1.55 eV)

Ce	13.681060	8.457120	10.267320
Ce	12.316160	14.973840	7.358320
Ce	13.979620	14.587981	10.432060
Ce	10.983140	6.369660	11.499979
Ce	5.624620	8.143520	7.953760
Ce	9.057800	7.367000	14.352540
Ce	9.246900	13.317800	8.354640
Ce	10.016700	10.054420	10.002919
Ce	7.348260	7.203220	11.116160
Ce	5.788340	8.685120	14.106340
Ce	11.257740	12.216820	5.439660
Ce	6.533980	11.342560	9.573180
Ce	11.275200	12.853020	12.120260
Ce	8.938800	6.896040	7.804320
Ce	14.690400	11.445620	11.847000
Ce	13.404760	11.590540	8.339440
Ce	12.009680	9.391460	13.448900
Ce	4.132920	9.049260	11.029140
Ce	11.725320	8.826380	6.487540
Ce	8.557680	10.899980	13.346660

Ce	8.270320	10.267180	6.387940
O	14.074500	13.653760	8.258820
O	7.004500	6.506520	8.908360
O	13.132960	16.010780	9.065020
O	9.555120	12.809380	13.417720
O	4.563320	11.011519	10.470639
O	11.175320	7.084060	13.630520
O	7.818520	8.572040	12.900300
O	7.452380	7.891900	6.570560
O	9.205200	12.488821	6.146920
O	12.956080	10.863740	6.251100
O	10.902441	6.492060	6.807320
O	14.208640	9.229320	12.235121
O	7.135080	7.368480	15.260640
O	13.140180	12.376360	10.516580
O	12.382380	9.539380	8.698380
O	10.325140	12.495620	10.141439
O	13.011800	11.606340	13.195720
O	9.774980	8.895780	7.639120
O	11.747880	8.485980	11.253680
O	9.380740	7.409560	10.002340
O	6.250140	8.903620	9.973660
O	15.480340	13.369200	11.526361
O	7.210140	13.322320	9.088640
O	12.434880	14.683640	11.838160
O	6.674320	10.555021	14.404840
O	4.003520	9.418260	13.134060
O	14.808060	10.344480	9.770220
O	12.143940	14.145080	5.325880
O	7.978000	10.798740	11.180120
O	3.812840	8.097900	9.033960
O	10.573300	10.638480	12.211061
O	10.420420	10.139199	5.190600
O	8.570700	11.092620	8.470960
O	9.874720	9.379680	14.594780
O	13.136801	6.301400	10.192519
O	10.304060	15.178760	7.903280
O	9.597580	3.723500	8.749500
O	11.551400	12.746021	7.613860
O	5.441340	7.413480	12.134399
O	6.098000	10.200760	7.514520
O	8.804280	6.082760	12.440881
O	11.411901	3.131320	10.134000
Ag	12.195200	6.126400	8.369040
C	10.515560	3.423240	9.427900

66

Structure **Ag3a** E = -514.868 eV (E^f = -4.99 eV)

Ce	13.787520	8.436980	10.191100
Ce	12.341280	14.863660	7.218800
Ce	13.911721	14.671040	10.403700
Ce	10.492680	6.367560	11.901340
Ce	5.649840	8.140820	8.094620
Ce	8.735800	7.707040	14.660701
Ce	9.286240	13.281580	8.265760
Ce	9.921600	10.099100	10.087759
Ce	7.092220	7.432000	11.334380
Ce	5.520240	9.156760	14.184180
Ce	11.311001	11.893899	5.512580
Ce	6.495680	11.440620	9.532340
Ce	11.285980	12.908840	12.102081
Ce	9.034920	6.663540	8.197380
Ce	14.625200	11.515180	11.935740
Ce	13.379580	11.588700	8.498920
Ce	11.994740	9.368660	13.450900
Ce	3.971600	9.335220	11.007380
Ce	11.856360	8.560420	6.998040
Ce	8.474220	11.059480	13.370720
Ce	8.417880	9.978660	6.616640
O	14.045700	13.652300	8.258960
O	6.801720	6.605120	9.256120
O	12.991900	15.963860	8.926160
O	9.552340	12.892700	13.367500
O	4.497900	11.263440	10.314161
O	10.808620	7.209640	13.912660
O	7.574420	8.839880	13.098140
O	11.573840	4.345260	11.337140
O	7.504520	7.904640	6.805520
O	9.198540	12.136620	6.173080
O	12.909859	10.601200	6.478980
O	10.887240	6.684600	6.812080
O	14.145319	9.430640	12.374001
O	6.798680	7.897020	15.500780
O	13.082560	12.492920	10.579540
O	12.111681	9.794780	9.149500
O	10.292660	12.412800	10.114080
O	12.846020	11.691160	13.240240
O	9.815040	8.848380	8.039300
O	11.940041	7.897960	11.487679
O	9.187500	7.755100	10.513620
O	6.118120	9.106000	10.080820
O	15.358300	13.497580	11.585840
O	7.252120	13.362660	8.952160
O	12.319740	14.782380	11.777340
O	6.531620	10.983200	14.358020
O	3.840480	9.925800	13.068500
O	14.808800	10.502400	9.847460
O	12.183141	13.796639	5.253860

O	7.985520	10.887380	11.167860
O	3.767000	8.269420	9.071320
O	10.555160	10.529640	12.147220
O	10.411921	9.801420	5.527500
O	8.593060	11.048460	8.544440
O	9.713740	9.634000	14.684180
O	13.537240	7.591840	8.045260
O	10.285140	15.087600	7.673920
O	10.224460	5.131360	9.760100
O	11.538121	12.688860	7.606420
O	5.160720	7.782540	12.296420
O	6.274160	10.207640	7.519800
O	8.465640	6.284620	12.804340
O	12.006299	3.833800	9.166040
Ag	12.716920	5.569840	7.999640
C	11.301920	4.383640	10.063440

66
Structure **Ag3b** E = -514.231 eV (E^f = -4.36 eV)

Ce	13.784000	8.425360	10.126539
Ce	12.350460	14.876400	7.225140
Ce	13.932860	14.649140	10.403920
Ce	10.497980	6.332300	11.815320
Ce	5.605920	8.209760	8.065380
Ce	8.766940	7.655000	14.606839
Ce	9.289960	13.302780	8.282120
Ce	9.918980	10.097340	10.060720
Ce	7.090720	7.436460	11.287400
Ce	5.552380	9.111180	14.179161
Ce	11.297320	11.927660	5.494900
Ce	6.492900	11.467659	9.550600
Ce	11.306100	12.885621	12.099460
Ce	8.977780	6.699280	8.127280
Ce	14.644940	11.479861	11.899340
Ce	13.371660	11.590220	8.473300
Ce	12.017200	9.321280	13.402539
Ce	3.971360	9.350000	11.019620
Ce	11.812780	8.580380	6.933540
Ce	8.496980	11.024880	13.364280
Ce	8.384260	10.045820	6.595940
O	14.054520	13.648500	8.244820
O	6.764580	6.644520	9.191440
O	13.011260	15.950580	8.942520
O	9.568440	12.862880	13.359060
O	4.499500	11.287659	10.344580
O	10.823560	7.162380	13.846300
O	7.586880	8.815240	13.070900
O	11.161060	4.134000	11.482379
O	7.465360	7.972120	6.771280

O	9.180320	12.184120	6.173800
O	12.887419	10.606240	6.439040
O	10.795820	6.725900	6.691260
O	14.175160	9.395780	12.318780
O	6.834320	7.829180	15.463740
O	13.092440	12.477280	10.561720
O	12.098540	9.794960	9.102620
O	10.301040	12.409860	10.112680
O	12.871220	11.647220	13.211740
O	9.789920	8.875480	7.993360
O	11.966320	7.869020	11.438900
O	9.182920	7.723940	10.429879
O	6.106240	9.131280	10.059000
O	15.384800	13.462300	11.559020
O	7.267480	13.393060	8.998180
O	12.351000	14.749939	11.792920
O	6.568200	10.932580	14.375840
O	3.868080	9.909360	13.084919
O	14.808960	10.492700	9.795100
O	12.178100	13.821959	5.249820
O	7.996160	10.868200	11.158080
O	3.736680	8.323980	9.060500
O	10.572460	10.500240	12.116580
O	10.383281	9.848220	5.495780
O	8.581500	11.073360	8.537420
O	9.743640	9.586840	14.650400
O	13.466140	7.539920	8.012980
O	10.299060	15.104340	7.695300
O	10.437419	5.147500	9.622040
O	11.534441	12.699260	7.597040
O	5.168260	7.771320	12.267120
O	6.244240	10.278380	7.513300
O	8.468760	6.276540	12.719460
O	11.217340	2.995380	9.500800
Ag	12.263080	5.730320	8.292980
C	10.967780	3.974600	10.190721

65

Structure **Ag3aV** E = -507.797 eV (E^f = -2.81 eV)

Ce	13.538102	8.058990	10.431672
Ce	12.540939	14.707506	7.437482
Ce	14.104794	14.272462	10.559289
Ce	10.384144	6.209019	11.723022
Ce	5.414338	8.279461	7.949961
Ce	8.528684	7.482332	14.476670
Ce	9.361063	13.217186	8.366929
Ce	9.825388	9.924528	10.100286
Ce	6.911726	7.339417	11.158057
Ce	5.377674	9.031994	14.067707

Ce	11.316772	12.020667	5.506157
Ce	6.461554	11.442732	9.519197
Ce	11.272815	12.685684	12.168000
Ce	8.674182	6.795750	7.895086
Ce	14.557153	11.084822	12.026254
Ce	13.374476	11.271906	8.540974
Ce	11.721892	9.164369	13.497705
Ce	3.847259	9.377244	10.921745
Ce	11.531295	8.693883	6.676916
Ce	8.361518	10.940309	13.356009
Ce	8.176442	10.200210	6.439967
O	14.169452	13.293259	8.395752
O	6.552060	6.612148	9.014386
O	13.379906	15.718617	9.116993
O	9.501768	12.775482	13.373462
O	4.479291	11.319031	10.362117
O	10.619582	7.031150	13.833352
O	7.419434	8.722294	12.965410
O	13.545213	5.592638	9.329330
O	7.227911	7.979505	6.633974
O	9.283349	12.381870	6.182290
O	12.859228	10.487148	6.371043
O	10.744778	6.630856	6.547320
O	13.948454	8.996201	12.464699
O	6.585077	7.689928	15.331017
O	13.106089	12.145149	10.649148
O	12.191538	9.325759	8.799336
O	10.375715	12.177458	10.120790
O	12.794546	11.393665	13.300452
O	9.667500	8.848993	7.796096
O	11.443932	8.204030	11.298642
O	8.976799	7.528087	10.181745
O	5.974228	9.094481	9.967883
O	15.465357	12.992904	11.714579
O	7.338921	13.372200	9.065459
O	12.485955	14.475935	11.879847
O	6.410903	10.858550	14.298402
O	3.686375	9.853945	13.008608
O	14.686391	10.068378	9.947554
O	12.308356	13.871373	5.380684
O	7.948013	10.709594	11.109636
O	3.544730	8.412946	8.948202
O	10.387715	10.480931	12.195541
O	10.371373	9.929609	5.316399
O	8.548551	11.022556	8.534665
O	9.517030	9.445678	14.638069
O	10.527278	15.008750	7.972136
O	11.579288	4.684886	10.044059
O	11.631216	12.542825	7.654384
O	4.988592	7.727673	12.129956

O	6.032454	10.364832	7.508479
O	8.208993	6.152107	12.608360
O	12.975769	5.698525	11.495333
Ag	12.188461	5.647953	7.646927
C	12.708101	5.263795	10.304627

65

Structure **Ag3bV** E = -507.533 eV (E^f = -2.54 eV)

Ce	13.681480	8.583600	10.210540
Ce	12.301660	14.986000	7.281680
Ce	13.919240	14.750720	10.451380
Ce	10.474460	6.205880	11.801521
Ce	5.544460	8.132260	8.102300
Ce	8.778740	7.671820	14.609261
Ce	9.319180	13.203760	8.223620
Ce	9.929240	10.030100	10.069600
Ce	7.042840	7.436480	11.386580
Ce	5.556300	9.185160	14.241120
Ce	11.350820	12.040020	5.456360
Ce	6.517380	11.338700	9.463280
Ce	11.291280	12.883540	12.063460
Ce	8.774020	6.596520	8.305220
Ce	14.646699	11.563200	11.928020
Ce	13.433460	11.733860	8.454080
Ce	11.939760	9.302540	13.430560
Ce	3.967100	9.386700	11.067520
Ce	12.069440	8.795160	6.860860
Ce	8.493140	11.041660	13.343400
Ce	8.457640	9.866740	6.453780
O	14.083420	13.795080	8.299040
O	6.706440	6.537280	9.327420
O	12.968560	16.047140	9.046220
O	9.573520	12.891120	13.314060
O	4.541000	11.278780	10.340540
O	10.836520	7.208380	13.875620
O	7.578600	8.865180	13.101300
O	11.076360	3.951400	11.209180
O	7.345780	7.723740	6.854620
O	9.291280	12.232760	6.137820
O	13.103060	10.812320	6.442940
O	14.142580	9.364180	12.241720
O	6.858620	7.903540	15.505140
O	13.134201	12.535360	10.570400
O	12.161601	9.866340	9.030640
O	10.325921	12.344400	10.088040
O	12.903640	11.659141	13.202519
O	9.985320	8.433860	7.692360
O	11.932200	8.002960	11.327339
O	9.099320	7.717840	10.383780

O	6.079880	9.077480	10.097980
O	15.393240	13.508720	11.615900
O	7.291280	13.249440	8.885520
O	12.328219	14.772700	11.818039
O	6.602900	11.010780	14.390600
O	3.897680	10.000360	13.137300
O	14.791380	10.482841	9.826520
O	12.168300	14.005700	5.294280
O	8.042440	10.800720	11.110060
O	3.687720	8.314980	9.142400
O	10.580760	10.515140	12.105980
O	10.655520	9.942640	5.359620
O	8.697380	10.865920	8.477240
O	9.769920	9.640220	14.627520
O	13.574880	7.463360	8.297740
O	10.269120	15.103641	7.768880
O	10.423599	5.020840	9.316520
O	11.599280	12.767839	7.603180
O	5.129860	7.814440	12.369680
O	6.184420	10.149940	7.535360
O	8.383180	6.287920	12.805800
O	10.867140	2.759320	9.268920
Ag	12.181820	5.994340	8.675640
C	10.823680	3.765100	9.953720

Structures at Fig. 2

65

Structure **1a** E = -509.962 eV (E^f = -0.26 eV)

Ce	13.576699	8.247640	9.982700
Ce	12.362480	14.891280	7.353400
Ce	14.014480	14.499320	10.472380
Ce	10.854720	6.310160	11.198660
Ce	5.722540	8.115660	7.978460
Ce	9.081680	7.314720	14.263080
Ce	9.287100	13.303860	8.360760
Ce	10.002620	10.037121	10.021460
Ce	7.281720	7.182100	11.116940
Ce	5.794840	8.669300	14.118040
Ce	11.288080	12.059280	5.493820
Ce	6.527360	11.357940	9.583620
Ce	11.296480	12.822840	12.148020
Ce	9.123740	6.703300	7.974520
Ce	14.646580	11.264040	11.793140
Ce	13.435481	11.532320	8.377660
Ce	11.953880	9.258480	13.185500
Ce	4.131080	9.073980	11.013200

Ce	11.848900	8.646380	6.761660
Ce	8.540040	10.880820	13.366020
Ce	8.428960	10.092040	6.563160
O	14.108520	13.607080	8.288100
O	6.961520	6.511600	8.955800
O	13.077900	15.881480	9.122100
O	9.587940	12.754660	13.458520
O	4.551080	11.052099	10.408840
O	11.186280	7.119860	13.420080
O	7.785920	8.578900	12.914660
O	12.914940	6.294060	10.601439
O	7.575140	7.978820	6.666940
O	9.185400	12.266639	6.217400
O	12.914280	10.720080	6.300920
O	10.774940	6.796680	6.583220
O	13.997940	9.118640	12.173740
O	7.192260	7.319880	15.214580
O	13.119640	12.349499	10.548840
O	12.076440	9.880540	8.901400
O	10.302440	12.471280	10.174000
O	12.888499	11.382600	13.177980
O	9.860000	8.899900	7.867260
O	11.571920	8.492040	11.079400
O	9.178240	7.839080	10.195720
O	6.234900	8.948560	10.005220
O	15.470181	13.193760	11.545280
O	7.252860	13.317360	9.061700
O	12.491800	14.608620	11.929100
O	6.722180	10.527380	14.486260
O	4.088900	9.517860	13.113900
O	14.770920	10.288900	9.765120
O	12.159660	13.977920	5.329220
O	7.982120	10.859040	11.204600
O	3.876580	8.122540	9.012160
O	10.531240	10.594480	12.136260
O	10.386159	9.971080	5.402340
O	8.628440	11.050640	8.554240
O	10.006540	9.335900	14.455980
O	13.497080	7.844500	7.880620
O	10.324500	15.126899	7.842760
O	10.095180	5.428300	9.397620
O	11.569420	12.717021	7.629300
O	5.377900	7.431440	12.156520
O	6.299220	10.218660	7.500100
O	8.811060	6.075620	12.407320
O	8.225300	3.436080	5.722980
C	8.420360	4.349460	6.373580

64
Structure **1aV** E = -503.243 eV (E^f = 1.57 eV)

Ce	13.673820	8.429780	9.851780
Ce	12.324780	14.946160	7.280800
Ce	13.986681	14.565020	10.410880
Ce	10.400820	6.212120	11.320961
Ce	5.553140	8.238360	8.024760
Ce	8.866820	7.385040	14.359961
Ce	9.252380	13.346601	8.274040
Ce	9.847220	10.127820	9.942560
Ce	7.047020	7.340920	11.181800
Ce	5.666400	8.943480	14.174780
Ce	11.246040	12.058460	5.454040
Ce	6.460180	11.474960	9.537640
Ce	11.378840	12.729959	12.067680
Ce	8.932240	6.777300	8.020840
Ce	14.697540	11.324880	11.732920
Ce	13.370820	11.622159	8.324880
Ce	12.024800	9.070060	13.168900
Ce	4.000460	9.317440	11.055779
Ce	11.697021	8.657260	6.803840
Ce	8.596420	10.855700	13.332160
Ce	8.270680	10.131320	6.433680
O	14.073180	13.681380	8.212220
O	6.808620	6.615020	9.077540
O	13.034000	15.937200	9.069580
O	9.711160	12.676980	13.405319
O	4.497500	11.269380	10.410380
O	10.872540	6.923640	13.430279
O	7.642540	8.669560	12.969919
O	10.605700	6.799520	6.658800
O	7.373140	7.910140	6.743440
O	9.206540	12.393640	6.126540
O	12.866920	10.692080	6.352820
O	14.150240	9.139120	11.957480
O	6.988260	7.544400	15.318840
O	13.148561	12.389081	10.480680
O	12.070220	9.840680	8.985980
O	10.299340	12.416900	10.104480
O	12.998320	11.395400	13.078820
O	9.742360	8.913900	7.905260
O	11.955640	7.748220	11.044440
O	9.109760	7.839980	10.330141
O	6.100120	9.097840	10.051841
O	15.474840	13.275000	11.479440
O	7.228680	13.419440	9.015280
O	12.468120	14.596519	11.864400
O	6.744080	10.729840	14.454900

O	4.009600	9.845420	13.154640
O	14.788880	10.369020	9.596900
O	12.158880	13.986599	5.292560
O	7.994560	10.875200	11.151839
O	3.710920	8.301780	9.112420
O	10.617599	10.386140	12.002640
O	10.432820	9.955540	5.376100
O	8.516340	11.136940	8.483540
O	9.898780	9.297260	14.479340
O	13.396720	7.709500	7.918680
O	10.288960	15.178360	7.784040
O	9.970660	5.394660	9.461440
O	11.528161	12.763439	7.594240
O	5.168440	7.668580	12.243659
O	6.072360	10.292380	7.544780
O	8.456140	6.089300	12.457180
O	8.424240	3.420360	5.711920
C	8.457780	4.335000	6.390500

65

Structure **2a** E = -511.392 eV (E^f = -1.69 eV)

Ce	13.410760	8.362040	10.133120
Ce	12.403840	14.953541	7.339580
Ce	14.036080	14.628500	10.484000
Ce	10.960580	6.307400	11.651860
Ce	5.693860	8.160540	7.976220
Ce	9.041200	7.539660	14.445800
Ce	9.261520	13.424420	8.246180
Ce	9.980220	10.334500	10.032820
Ce	7.353500	7.110380	11.142820
Ce	5.766020	8.797560	14.093440
Ce	11.373000	12.080940	5.540200
Ce	6.507460	11.412920	9.494860
Ce	11.271740	13.021860	12.160220
Ce	8.957640	6.721340	7.784300
Ce	14.595400	11.407400	11.874340
Ce	13.468781	11.632140	8.442520
Ce	11.870240	9.466880	13.307920
Ce	4.199100	9.088020	11.020221
Ce	11.766640	8.709980	6.939220
Ce	8.497000	11.041580	13.344600
Ce	8.357900	10.160480	6.473260
O	14.162240	13.692540	8.328460
O	6.956980	6.521600	8.846440
O	13.108600	15.978880	9.115640
O	9.520600	12.958140	13.392760
O	4.599660	11.091260	10.392060
O	11.245800	7.321180	13.704760
O	7.848300	8.684660	12.963240

O	12.895679	6.344580	10.844680
O	7.493880	7.924680	6.583000
O	9.310580	12.433840	6.123160
O	12.991900	10.737700	6.454180
O	10.730079	6.797800	6.639260
O	13.838880	9.252800	12.251199
O	7.139340	7.534100	15.331640
O	13.104481	12.489420	10.595000
O	12.018840	9.983120	9.010620
O	10.287480	12.610860	10.152940
O	12.866840	11.589500	13.252120
O	9.715780	8.808060	8.099320
O	11.307460	8.499500	11.213360
O	9.547140	6.460800	9.961740
O	6.302100	8.968460	9.981200
O	15.460920	13.305520	11.596900
O	7.227960	13.397380	8.925060
O	12.498940	14.780140	11.932720
O	6.693260	10.705700	14.412820
O	4.077900	9.639760	13.111340
O	14.703360	10.357220	9.879740
O	12.294700	14.000980	5.359720
O	8.029920	10.902960	11.135740
O	3.867840	8.122460	9.071600
O	10.541019	10.798481	12.159300
O	10.539020	9.970460	5.524600
O	8.649120	11.187741	8.492840
O	9.967780	9.602840	14.533340
O	13.376319	7.833400	8.086720
O	10.352620	15.229660	7.766260
O	9.775860	3.858820	8.240800
O	11.610320	12.797480	7.670220
O	5.380540	7.518460	12.236320
O	6.185340	10.266581	7.522920
O	8.967280	6.146120	12.722100
O	10.866580	3.642380	10.313120
C	10.317180	3.774520	9.281960

64

Structure **2aV** E = -504.355 eV (E^f = 0.46 eV)

Ce	13.611816	8.437173	10.397642
Ce	12.409087	14.919294	7.342686
Ce	14.015371	14.604383	10.448699
Ce	10.939112	6.448913	11.593568
Ce	5.631711	8.231695	8.029633
Ce	8.996208	7.555361	14.433488
Ce	9.284772	13.378422	8.315511
Ce	9.965382	10.127821	10.029062
Ce	7.301164	7.299048	11.200873

Ce	5.739790	8.879451	14.169016
Ce	11.341469	12.108634	5.486237
Ce	6.521578	11.492018	9.550198
Ce	11.223749	13.004944	12.115806
Ce	8.917375	6.892729	7.865661
Ce	14.609025	11.473848	11.960894
Ce	13.415415	11.543945	8.425410
Ce	11.856959	9.542287	13.417760
Ce	4.136975	9.208803	11.063799
Ce	11.688821	8.709851	6.649615
Ce	8.471621	11.107664	13.352199
Ce	8.295226	10.269750	6.428820
O	14.124002	13.601639	8.318328
O	6.924283	6.583848	9.034202
O	13.230869	15.995505	9.050190
O	9.489200	13.005589	13.388884
O	4.551050	11.164622	10.453770
O	11.162784	7.341497	13.730310
O	7.785414	8.747561	12.966882
O	10.821860	3.711089	10.281634
O	7.400879	7.938222	6.642833
O	9.285195	12.471478	6.145973
O	13.006433	10.784965	6.366532
O	13.996251	9.311909	12.444416
O	7.105447	7.594525	15.364318
O	13.112922	12.412885	10.598897
O	12.347925	9.540473	8.819413
O	10.307511	12.596316	10.158724
O	12.841574	11.633070	13.292103
O	9.730053	8.953777	7.782470
O	11.604220	8.636785	11.359378
O	9.341856	7.392077	10.117793
O	6.265355	9.028895	10.043871
O	15.456071	13.354475	11.639310
O	7.237662	13.449880	9.024113
O	12.461468	14.774272	11.843299
O	6.635730	10.754657	14.461436
O	3.999474	9.621890	13.184615
O	14.778143	10.341868	9.954221
O	12.258842	14.038688	5.344873
O	7.925441	10.985277	11.215373
O	3.806577	8.225353	9.120299
O	10.498209	10.783405	12.245733
O	10.466583	10.062939	5.277689
O	8.563654	11.192058	8.497996
O	9.910247	9.602425	14.595281
O	13.034389	6.480905	10.508981
O	10.405800	15.196875	7.872999
O	10.706735	6.586673	6.896508
O	11.598250	12.720119	7.659456

O	5.405601	7.571613	12.228749
O	6.109908	10.292617	7.554370
O	8.744698	6.228669	12.635756
O	9.556694	3.674646	8.300951
C	10.196291	3.678024	9.283253

64

Structure **2bV** E = -503.616 eV (E^f = 1.20 eV)

Ce	13.454598	8.347533	10.070683
Ce	12.351174	14.999535	7.398312
Ce	14.047512	14.619155	10.513240
Ce	10.839562	6.433674	11.548903
Ce	5.580431	8.172997	8.055711
Ce	8.991822	7.579432	14.445232
Ce	9.295998	13.287107	8.272578
Ce	9.950389	10.075647	10.087578
Ce	7.204627	7.313527	11.256292
Ce	5.714976	8.925175	14.203138
Ce	11.358187	12.168926	5.482345
Ce	6.521049	11.391480	9.502044
Ce	11.244916	12.987218	12.110070
Ce	8.779114	6.567322	8.081801
Ce	14.613453	11.357193	11.869302
Ce	13.456752	11.677208	8.460097
Ce	11.894262	9.475855	13.307033
Ce	4.083482	9.250844	11.080923
Ce	12.021043	8.892490	6.784667
Ce	8.474794	11.109608	13.355306
Ce	8.420990	9.986888	6.449080
O	14.149172	13.727157	8.353092
O	6.771321	6.500503	9.190702
O	13.085532	15.985471	9.174494
O	9.516850	12.989315	13.397560
O	4.541118	11.174309	10.406532
O	11.143944	7.290624	13.610164
O	7.742028	8.764544	12.997011
O	12.761663	6.373667	10.676062
O	7.340189	7.801284	6.753789
O	9.310802	12.334628	6.117147
O	13.087475	10.827296	6.392063
O	13.926847	9.229273	12.239496
O	7.112882	7.614353	15.376941
O	13.112268	12.455715	10.591127
O	12.073202	9.909568	8.855544
O	10.273789	12.622437	10.158876
O	12.878382	11.539564	13.237957
O	10.003815	8.393522	7.489660
O	11.512886	8.579285	11.243427

O	9.210612	7.523425	10.181147
O	6.205486	9.022020	10.073835
O	15.474306	13.254906	11.592473
O	7.248445	13.316145	8.896517
O	12.519372	14.738042	11.949492
O	6.662519	10.774977	14.506190
O	4.013209	9.725093	13.196018
O	14.697499	10.318532	9.825026
O	12.216151	14.103802	5.363507
O	7.900323	11.005384	11.224226
O	3.748699	8.261359	9.146981
O	10.505495	10.772796	12.233073
O	10.678123	10.013096	5.371411
O	8.652349	10.969377	8.444529
O	9.939979	9.563979	14.561740
O	13.361715	7.745663	7.940683
O	10.319775	15.172794	7.830346
O	10.183696	4.709839	9.051014
O	11.621230	12.793566	7.639737
O	5.325244	7.609552	12.298259
O	6.161122	10.197217	7.528729
O	8.783400	6.263572	12.612407
O	10.835011	3.908315	11.080248
C	10.551928	3.832138	9.879084

65

Structure **3a** E = -512.287 eV (E^f = -2.59 eV)

Ce	13.679620	8.440200	9.928040
Ce	12.337379	14.943900	7.289500
Ce	13.997580	14.582880	10.426340
Ce	10.498080	6.220220	11.548760
Ce	5.576260	8.202660	8.053560
Ce	8.884900	7.468040	14.475600
Ce	9.266500	13.331120	8.277660
Ce	9.865460	10.125180	9.985500
Ce	7.109900	7.352000	11.232560
Ce	5.667240	8.980260	14.192060
Ce	11.279880	12.041260	5.472200
Ce	6.469200	11.453900	9.537840
Ce	11.380980	12.757601	12.082380
Ce	8.939560	6.798860	8.026500
Ce	14.710040	11.350760	11.770279
Ce	13.389100	11.627580	8.358980
Ce	12.040560	9.147700	13.253779
Ce	4.012900	9.284080	11.063720
Ce	11.729360	8.663280	6.858260
Ce	8.584000	10.897440	13.349360
Ce	8.291960	10.121320	6.449360

O	14.086000	13.685300	8.231340
O	6.868780	6.612540	9.103240
O	13.014060	15.923820	9.085200
O	9.700600	12.714680	13.404460
O	4.505000	11.229000	10.408180
O	10.911460	6.995100	13.605320
O	7.675380	8.700920	13.013721
O	10.610980	6.786620	6.732940
O	7.408600	7.886580	6.760080
O	9.232420	12.365119	6.134680
O	12.895041	10.676820	6.392360
O	14.169480	9.174180	12.013420
O	6.991060	7.649680	15.398000
O	13.158900	12.401900	10.506140
O	12.073560	9.845760	9.026400
O	10.307480	12.404220	10.117300
O	12.998680	11.437060	13.116080
O	9.756160	8.904840	7.944480
O	11.948220	7.815480	11.165500
O	9.168880	7.720560	10.322241
O	6.123700	9.072800	10.070360
O	15.481761	13.295280	11.501660
O	7.237320	13.391820	9.010940
O	12.471800	14.612679	11.873960
O	6.707060	10.788980	14.428400
O	3.985260	9.817800	13.155100
O	14.795300	10.372360	9.645240
O	12.195400	13.957660	5.306420
O	8.006440	10.849880	11.154200
O	3.735340	8.247680	9.128640
O	10.614040	10.412461	12.048841
O	10.468060	9.929780	5.422240
O	8.537740	11.116199	8.499540
O	9.888780	9.393900	14.549780
O	13.400260	7.717820	7.999200
O	10.295240	15.155340	7.780180
O	9.926360	5.141840	9.426340
O	11.547420	12.752680	7.610000
O	5.221980	7.669120	12.270499
O	6.089180	10.258620	7.561600
O	8.514120	6.141760	12.580740
O	11.385880	4.255540	10.842140
O	10.878960	3.125380	8.921240
C	10.765560	4.073560	9.675520

65

Structure **3b** E = -511.976 eV (E^f = -2.28 eV)

Ce	13.399448	8.143046	10.068891
Ce	12.473136	14.688168	7.351822

Ce	14.097947	14.298844	10.495409
Ce	10.327573	6.181650	11.836358
Ce	5.621186	8.077869	8.023380
Ce	8.619720	7.652010	14.572533
Ce	9.337850	13.198919	8.340488
Ce	9.866635	9.887972	10.095898
Ce	6.953694	7.318568	11.270983
Ce	5.433474	9.130423	14.095621
Ce	11.286644	11.873506	5.516890
Ce	6.483026	11.386510	9.497280
Ce	11.288677	12.759283	12.115793
Ce	9.042802	6.617812	8.094454
Ce	14.565853	11.070423	11.907418
Ce	13.388617	11.316383	8.450449
Ce	11.753385	9.211070	13.324796
Ce	3.902500	9.301659	10.901084
Ce	11.752119	8.443136	6.773096
Ce	8.382016	11.057926	13.323543
Ce	8.378174	9.955232	6.597959
O	14.161152	13.343835	8.321655
O	6.704585	6.505170	9.154577
O	13.235129	15.681949	9.102962
O	9.537413	12.861331	13.344145
O	4.463500	11.223196	10.236671
O	10.777552	7.217363	13.869996
O	7.481933	8.802404	13.023533
O	13.251250	5.769538	10.090512
O	7.483140	7.868692	6.764238
O	9.203249	12.136382	6.227572
O	12.849898	10.506103	6.376499
O	10.714905	6.710433	6.501228
O	13.828382	8.974074	12.285719
O	6.711362	7.881564	15.435729
O	13.113413	12.199723	10.602486
O	11.957345	9.750180	9.135614
O	10.288326	12.366514	10.177140
O	12.777330	11.277820	13.253093
O	9.794932	8.763614	7.971244
O	11.475790	8.176717	11.342170
O	8.973296	7.638168	10.389359
O	6.064439	9.039595	10.016506
O	15.472440	12.950879	11.638376
O	7.287570	13.304102	8.977147
O	12.549677	14.502110	11.906319
O	6.461728	10.957780	14.317963
O	3.792221	9.917443	12.971739
O	14.672976	10.061333	9.883643
O	12.231481	13.759111	5.338694
O	7.916408	10.839546	11.152731
O	3.718732	8.230976	8.976797

O	10.425428	10.513779	12.167970
O	10.322642	9.806367	5.441121
O	8.602595	10.953118	8.564295
O	9.696399	9.575664	14.571460
O	13.492835	7.877259	8.004348
O	10.449476	14.992343	7.844639
O	11.090724	5.788445	9.403669
O	11.593806	12.549168	7.652627
O	5.037025	7.728542	12.220306
O	6.265747	10.180112	7.469743
O	8.257853	6.203771	12.827345
O	11.739715	4.347045	10.983800
C	12.072343	5.235350	10.113149

64

Structure **3aV** E = -506.422 eV (E = -1.61 eV)

Ce	13.637080	8.318460	10.470760
Ce	12.452240	14.695820	7.247440
Ce	14.028540	14.491900	10.401040
Ce	10.579920	6.367080	11.847000
Ce	5.603460	8.097580	8.122500
Ce	8.753960	7.658820	14.627460
Ce	9.350300	13.170919	8.334260
Ce	9.980580	9.951800	10.204740
Ce	7.095740	7.352100	11.354080
Ce	5.546500	9.106860	14.201820
Ce	11.313460	11.792279	5.524080
Ce	6.527740	11.372640	9.596320
Ce	11.297680	12.884361	12.132540
Ce	8.959180	6.630840	8.117140
Ce	14.634280	11.382300	11.990520
Ce	13.390460	11.381680	8.527440
Ce	11.881940	9.420320	13.593680
Ce	3.974020	9.285980	11.053500
Ce	11.675180	8.439560	6.876940
Ce	8.455680	11.088940	13.402940
Ce	8.354840	9.971060	6.671220
O	14.115180	13.428980	8.273880
O	6.769640	6.526440	9.214580
O	13.210080	15.822880	8.897420
O	9.522820	12.961000	13.340040
O	4.524660	11.214460	10.384361
O	10.849280	7.223420	13.964120
O	7.580540	8.819200	13.094480
O	12.830480	5.845980	10.552180
O	7.446120	7.894880	6.805880
O	9.206080	12.089339	6.224220
O	12.883000	10.379720	6.445580
O	14.092979	9.286880	12.512760
O	6.807120	7.843100	15.494360

O	13.129179	12.348900	10.605381
O	12.151680	9.605580	9.058480
O	10.382780	12.303060	10.126220
O	12.877240	11.675800	13.285280
O	9.791280	8.770440	8.044640
O	11.599981	8.360560	11.477480
O	9.113960	7.660640	10.436500
O	6.106500	9.037340	10.093800
O	15.455199	13.311460	11.602900
O	7.324240	13.292540	9.027520
O	12.432480	14.705260	11.747240
O	6.516200	10.965821	14.378880
O	3.826560	9.857000	13.104320
O	14.784980	10.295100	9.939260
O	12.225540	13.663480	5.257020
O	8.008000	10.804780	11.199360
O	3.733560	8.223060	9.093300
O	10.490780	10.652280	12.259700
O	10.346820	9.741600	5.528000
O	8.631060	10.947680	8.616800
O	9.680260	9.647980	14.721700
O	10.634400	6.767200	6.467300
O	10.428420	14.964679	7.766100
O	10.984460	5.601020	9.307300
O	11.599560	12.528200	7.608000
O	5.161920	7.724000	12.316700
O	6.261120	10.199800	7.558780
O	8.443920	6.249100	12.809600
O	12.778380	6.781440	8.515980
C	12.203200	6.036780	9.417300

64
Structure **3bV** E = -505.725 eV (E^f = -0.91 eV)

Ce	13.713188	8.447992	10.080919
Ce	12.333939	14.959724	7.307807
Ce	13.952404	14.691890	10.477154
Ce	10.465950	6.342881	11.778183
Ce	5.538269	8.126681	8.088256
Ce	8.763759	7.667552	14.617682
Ce	9.329744	13.191507	8.221754
Ce	9.949438	10.044769	10.077997
Ce	7.015694	7.422029	11.368062
Ce	5.539359	9.170221	14.226288
Ce	11.378802	12.035233	5.474834
Ce	6.525575	11.334766	9.462366
Ce	11.303267	12.873899	12.083580
Ce	8.775423	6.561968	8.322073
Ce	14.642910	11.466039	11.908211
Ce	13.462861	11.693404	8.476187
Ce	12.004255	9.309128	13.387446

Ce	3.956391	9.387677	11.047585
Ce	12.031446	8.795012	6.891997
Ce	8.494025	11.026038	13.340819
Ce	8.445129	9.835294	6.465196
O	14.104048	13.761777	8.304616
O	6.695414	6.530101	9.323670
O	12.970840	15.996900	9.071474
O	9.563448	12.863241	13.338627
O	4.537997	11.274484	10.328109
O	10.837351	7.164108	13.800439
O	7.568558	8.827123	13.096311
O	11.370682	4.299822	11.297265
O	7.338120	7.716363	6.851971
O	9.320338	12.196332	6.123500
O	13.076226	10.749081	6.441956
O	14.127953	9.357826	12.281659
O	6.847183	7.871798	15.495411
O	13.111573	12.502124	10.570345
O	12.099333	9.879674	8.996130
O	10.305028	12.353042	10.096784
O	12.873515	11.628181	13.206061
O	9.988475	8.397305	7.693942
O	11.822147	7.984166	11.320965
O	9.079332	7.736154	10.401815
O	6.073254	9.067165	10.093092
O	15.391884	13.445467	11.611719
O	7.293683	13.239862	8.871377
O	12.362820	14.741557	11.858141
O	6.592419	10.975401	14.391427
O	3.893769	9.995229	13.118078
O	14.810003	10.489706	9.800909
O	12.205526	13.971885	5.298100
O	8.025920	10.806123	11.119741
O	3.682646	8.313649	9.123849
O	10.563519	10.515709	12.119834
O	10.680244	9.856653	5.422038
O	8.696929	10.851030	8.475949
O	9.748479	9.585207	14.641062
O	13.362241	7.628868	7.915304
O	10.283270	15.078319	7.755766
O	10.157392	5.133844	9.636039
O	11.614848	12.744223	7.605491
O	5.115363	7.797821	12.355720
O	6.188526	10.141912	7.530171
O	8.453373	6.279033	12.754045
O	11.086280	3.065778	9.390917
C	10.906186	4.062417	10.071455

Transition states

66

Pd1b->**Pd2a** E= -515.052 eV

Ce	13.528497	8.284063	10.076368
Ce	12.353375	14.885528	7.337977
Ce	13.990500	14.511685	10.469604
Ce	10.712384	6.213249	11.263853
Ce	5.675257	8.051382	7.954097
Ce	8.972190	7.385293	14.312516
Ce	9.280103	13.244594	8.284879
Ce	9.938955	9.984823	9.984344
Ce	7.193139	7.184300	11.147287
Ce	5.698846	8.764731	14.103777
Ce	11.333530	12.038670	5.472617
Ce	6.502632	11.310204	9.482318
Ce	11.252780	12.809110	12.089986
Ce	9.009258	6.623136	8.017067
Ce	14.608260	11.270796	11.839277
Ce	13.434660	11.543720	8.397372
Ce	11.872715	9.253265	13.224653
Ce	4.082309	9.091498	10.961465
Ce	11.797574	8.692063	6.755275
Ce	8.478605	10.907853	13.303130
Ce	8.360697	10.034201	6.432689
O	14.117647	13.612530	8.301300
O	6.902840	6.453339	9.013847
O	13.063295	15.885078	9.117390
O	9.530776	12.788491	13.360738
O	4.540068	11.059160	10.351198
O	11.097159	7.157533	13.523141
O	7.709311	8.627443	12.914008
O	12.959602	6.140994	10.278164
O	7.516404	7.772314	6.677788
O	9.285159	12.305688	6.129128
O	12.981997	10.666397	6.356958
O	11.034291	6.553634	6.727265
O	13.967531	9.097099	12.199880
O	7.077116	7.455115	15.260031
O	13.110784	12.338670	10.547777
O	12.095629	9.807000	8.888528
O	10.292687	12.402329	10.117614
O	12.850811	11.386874	13.176310
O	9.849478	8.781587	7.811763
O	11.568282	8.415329	11.129096
O	9.156477	7.682262	10.158193
O	6.192206	8.921326	9.985085
O	15.427855	13.189954	11.590116
O	7.235552	13.271354	8.958955

O	12.436759	14.610775	11.879283
O	6.657475	10.629255	14.416312
O	4.021330	9.591688	13.066113
O	14.736675	10.240477	9.787607
O	12.215588	13.969340	5.329474
O	7.988025	10.786844	11.114054
O	3.822728	8.081516	9.009570
O	10.499656	10.570716	12.102189
O	10.538424	9.908369	5.344913
O	8.619124	10.997707	8.486433
O	9.921465	9.415371	14.459331
O	13.369725	7.572318	7.933550
O	10.320423	15.087270	7.821008
O	9.938024	5.152230	9.187932
O	11.600034	12.688241	7.618269
O	5.292062	7.469451	12.172744
O	6.179902	10.135334	7.482029
O	8.650343	6.094273	12.516210
O	11.585306	2.821070	8.670796
Pd	12.252115	5.931397	8.321406
C	11.770340	3.950709	8.464581

66

Pd2a->**Pd3a** E = -515.03 eV

Ce	13.417709	8.129619	10.169537
Ce	12.526586	14.774808	7.413622
Ce	14.100096	14.339732	10.576081
Ce	10.612271	6.293610	11.643584
Ce	5.562329	8.294021	7.912483
Ce	8.729342	7.488469	14.402115
Ce	9.353794	13.328207	8.371503
Ce	9.901344	9.958868	10.070726
Ce	7.068275	7.255839	11.122111
Ce	5.534952	8.907740	14.054257
Ce	11.348518	12.017045	5.528511
Ce	6.506982	11.515149	9.533684
Ce	11.248982	12.828548	12.188376
Ce	8.791332	6.776918	7.831097
Ce	14.552782	11.102273	11.955411
Ce	13.432357	11.402271	8.513449
Ce	11.748195	9.311666	13.373662
Ce	3.999934	9.297729	10.902070
Ce	11.733500	8.664649	6.778853
Ce	8.386859	11.042942	13.359754
Ce	8.347759	10.196060	6.536962
O	14.201982	13.422014	8.389789
O	6.677840	6.588862	8.913318
O	13.245118	15.744302	9.190021
O	9.505183	12.869498	13.464553
O	4.487539	11.240693	10.293137

O	10.900906	7.147717	13.736768
O	7.584158	8.709028	12.889954
O	13.249888	7.395195	7.894071
O	7.390894	8.050832	6.558775
O	9.256924	12.291219	6.190457
O	12.925135	10.565917	6.389160
O	10.900372	6.589239	6.598591
O	13.837099	9.028599	12.407725
O	6.802604	7.560640	15.267955
O	13.086927	12.227659	10.640624
O	12.038732	9.690303	8.924998
O	10.282277	12.577369	10.212080
O	12.772520	11.356905	13.302095
O	9.696175	8.821214	7.796867
O	11.418024	8.377535	11.333561
O	9.090913	7.363796	10.123104
O	6.156338	9.071656	9.946266
O	15.472260	12.983906	11.682605
O	7.292279	13.443197	8.985789
O	12.575572	14.539233	12.004385
O	6.517174	10.715207	14.440473
O	3.865938	9.748173	12.996088
O	14.694082	10.133049	9.901025
O	12.339132	13.851407	5.372000
O	7.812946	11.030539	11.230990
O	3.713658	8.351744	8.917640
O	10.407348	10.632198	12.223241
O	10.389016	9.937130	5.408180
O	8.594050	11.105179	8.532461
O	9.728296	9.466095	14.556876
O	10.492716	15.099729	7.811802
O	10.308509	4.403282	8.604957
O	11.630197	12.625012	7.677773
O	5.154656	7.609859	12.092459
O	6.240163	10.359938	7.443411
O	8.436964	6.140758	12.605217
O	11.112542	3.484292	10.605847
O	12.497623	5.892093	10.263381
Pd	11.909150	5.989813	8.295227
C	10.904251	4.103989	9.620934

65

Pd1aV->**Pd2aV** E = -507.95 eV

Ce	13.520782	8.367182	10.151004
Ce	12.365857	14.925125	7.360011
Ce	14.033632	14.581244	10.482765
Ce	10.828450	6.359504	11.612782
Ce	5.610040	8.124849	8.064238
Ce	8.967289	7.537705	14.443638
Ce	9.272482	13.289430	8.299761

Ce	9.926778	9.992456	10.039718
Ce	7.221897	7.235308	11.230619
Ce	5.713360	8.891081	14.183861
Ce	11.325459	12.090275	5.485517
Ce	6.501915	11.388521	9.525463
Ce	11.241305	12.932971	12.093151
Ce	8.860679	6.633981	7.936038
Ce	14.623535	11.355877	11.892277
Ce	13.435316	11.605246	8.446974
Ce	11.874075	9.440317	13.330482
Ce	4.099224	9.189228	11.075195
Ce	11.863899	8.801565	6.803047
Ce	8.476502	11.061687	13.344481
Ce	8.328831	10.066593	6.463471
O	14.136260	13.646007	8.317876
O	6.790954	6.479000	9.135205
O	13.117744	15.940906	9.116737
O	9.538411	12.941923	13.389585
O	4.539468	11.133424	10.422822
O	11.140500	7.278622	13.693310
O	7.757141	8.721861	12.976996
O	13.009555	6.255724	10.479075
O	7.363858	7.831649	6.691981
O	9.287320	12.339542	6.111007
O	13.002669	10.685060	6.401384
O	13.969890	9.208253	12.273993
O	7.087506	7.615982	15.384935
O	13.122661	12.406009	10.577657
O	12.111274	9.827835	8.898045
O	10.276095	12.592242	10.152323
O	12.869300	11.514210	13.237675
O	9.865626	8.677396	7.698869
O	11.578417	8.489761	11.269538
O	9.230306	7.406982	10.191829
O	6.227898	8.988079	10.071614
O	15.474864	13.239874	11.600602
O	7.233293	13.339422	8.934906
O	12.517722	14.701576	11.910088
O	6.655684	10.753330	14.469080
O	4.001467	9.672200	13.189156
O	14.725525	10.284922	9.837077
O	12.221079	14.004862	5.339181
O	7.907861	10.970504	11.214443
O	3.768800	8.195990	9.152881
O	10.509739	10.699618	12.189906
O	10.575356	9.941409	5.371273
O	8.606926	11.031183	8.471248
O	9.930192	9.554644	14.553825
O	13.291230	7.552329	7.982379
O	10.346991	15.142983	7.814898

O	10.885403	5.574932	7.093967
O	11.607026	12.724723	7.633655
O	5.335672	7.565513	12.279429
O	6.137518	10.188559	7.550190
O	8.700048	6.170689	12.685417
O	10.474743	3.609364	9.340059
Pd	12.209122	5.928367	8.604636
C	11.103816	4.485754	8.877676

65

Pd2aV->**Pd3aV** E = -507.72 eV

Ce	13.443556	8.077055	10.266492
Ce	12.540590	14.726075	7.422222
Ce	14.095558	14.259930	10.558081
Ce	10.358716	6.167724	11.652467
Ce	5.435870	8.369206	7.903381
Ce	8.588336	7.464625	14.441422
Ce	9.368542	13.298146	8.409850
Ce	9.909225	9.905747	10.064039
Ce	6.925757	7.324304	11.136234
Ce	5.433449	8.993751	14.061435
Ce	11.334839	11.977757	5.539949
Ce	6.495197	11.534148	9.568560
Ce	11.262431	12.709668	12.190161
Ce	8.637811	6.831134	7.880165
Ce	14.540389	11.038266	11.963097
Ce	13.402119	11.300515	8.474177
Ce	11.731212	9.151844	13.438503
Ce	3.889050	9.395429	10.918276
Ce	11.600540	8.560356	6.702683
Ce	8.379114	10.968939	13.357133
Ce	8.291842	10.242236	6.536692
O	14.185219	13.325613	8.373553
O	6.601152	6.634721	8.874084
O	13.304957	15.700541	9.157988
O	9.511839	12.793965	13.431096
O	4.460042	11.315186	10.329641
O	10.650934	6.980466	13.737390
O	7.471663	8.704337	12.901169
O	13.681190	6.250686	9.040049
O	7.308016	8.072616	6.585058
O	9.223155	12.306434	6.211338
O	12.886713	10.541265	6.355687
O	10.405043	6.302593	6.839904
O	13.896978	8.917524	12.316258
O	6.651267	7.638775	15.293796
O	13.088181	12.121621	10.609354
O	12.141014	9.441438	8.846931
O	10.302006	12.437123	10.197896
O	12.826964	11.329702	13.277328

O	9.602160	8.819240	7.789356
O	11.440667	8.151850	11.264462
O	8.954890	7.391053	10.121120
O	6.033498	9.103632	9.930006
O	15.455089	12.929198	11.689111
O	7.318411	13.445733	9.056956
O	12.548838	14.459989	11.945324
O	6.440216	10.784818	14.370210
O	3.743080	9.835960	12.996314
O	14.661477	9.988433	9.875805
O	12.307264	13.832607	5.386425
O	7.811182	10.938537	11.197872
O	3.601518	8.457883	8.894453
O	10.388591	10.509067	12.229521
O	10.271542	9.944166	5.386728
O	8.554965	11.081192	8.522077
O	9.560008	9.395149	14.625329
O	10.523040	15.064561	7.887700
O	11.838830	4.178975	10.358788
O	11.611540	12.562915	7.684535
O	5.024152	7.695748	12.090123
O	6.168056	10.407460	7.429125
O	8.229640	6.121430	12.554013
O	13.123647	5.752177	11.343057
Pd	11.868165	5.949227	8.254086
C	12.597295	5.158018	10.373964

66

Ag1a->**Ag2b** E = -512.06 eV

Ce	13.541725	8.297839	10.063227
Ce	12.389405	14.922802	7.350781
Ce	14.012015	14.533889	10.494404
Ce	10.750632	6.300963	11.254985
Ce	5.703663	8.086783	7.962919
Ce	9.012026	7.360684	14.284687
Ce	9.307270	13.306472	8.351935
Ce	9.978755	10.023923	10.028652
Ce	7.255882	7.185542	11.110741
Ce	5.746498	8.740128	14.086641
Ce	11.371791	12.089643	5.492235
Ce	6.537402	11.369003	9.554530
Ce	11.264297	12.857832	12.123357
Ce	9.076271	6.697672	8.006638
Ce	14.629048	11.286178	11.832912
Ce	13.456519	11.549397	8.421791
Ce	11.904790	9.296060	13.204536
Ce	4.118193	9.104522	10.961363
Ce	11.833994	8.679358	6.813939
Ce	8.508300	10.930809	13.325026
Ce	8.433037	10.074037	6.569809

O	14.131142	13.641412	8.307635
O	6.945615	6.497795	8.950378
O	13.102362	15.918995	9.121995
O	9.539266	12.821747	13.402431
O	4.566583	11.082013	10.370511
O	11.129508	7.142423	13.470945
O	7.763896	8.602124	12.897426
O	12.874950	6.264884	10.582210
O	7.567880	7.915360	6.653852
O	9.238006	12.293057	6.155902
O	12.955630	10.714282	6.336765
O	10.815763	6.733447	6.663237
O	13.961626	9.119228	12.212938
O	7.125698	7.414128	15.236150
O	13.126481	12.377625	10.555898
O	12.142842	9.815211	8.875751
O	10.297910	12.539928	10.148511
O	12.887743	11.403786	13.206673
O	9.838147	8.893089	7.841623
O	11.552857	8.480550	11.142441
O	9.194344	7.687809	10.154107
O	6.235464	8.950440	9.964320
O	15.474061	13.229893	11.577893
O	7.256106	13.351891	9.012810
O	12.495248	14.652315	11.913939
O	6.670784	10.596297	14.426451
O	4.039379	9.555470	13.077285
O	14.751396	10.287875	9.803260
O	12.205521	13.984802	5.340073
O	7.936000	10.882143	11.179659
O	3.869758	8.148420	8.975319
O	10.534257	10.618297	12.159773
O	10.472848	9.951016	5.397450
O	8.616106	11.060221	8.459005
O	9.979615	9.380156	14.482440
O	13.472841	7.641519	7.908628
O	10.338155	15.141699	7.830788
O	10.140089	5.221158	9.377493
O	11.592783	12.728069	7.624112
O	5.338914	7.469296	12.150062
O	6.274816	10.218388	7.440544
O	8.755418	6.087090	12.437041
O	11.274574	2.876737	9.090962
Ag	12.625104	5.611342	8.107956
C	11.510327	3.944191	8.699328

66

Ag2b->**Ag3a** E = -512.58 eV

Ce	13.666947	8.261639	10.229263
Ce	12.372569	14.913189	7.303622

Ce	13.991292	14.620014	10.452758
Ce	10.806530	6.431295	11.679480
Ce	5.650565	8.140974	8.016481
Ce	8.907862	7.557183	14.468510
Ce	9.322891	13.292763	8.303754
Ce	10.036533	10.081996	10.098782
Ce	7.224705	7.309963	11.228403
Ce	5.659157	8.959917	14.140713
Ce	11.356200	12.018368	5.496908
Ce	6.541343	11.395684	9.539469
Ce	11.301368	12.943720	12.133580
Ce	8.973986	6.698586	7.962070
Ce	14.638857	11.412290	11.885778
Ce	13.458159	11.581028	8.476683
Ce	11.967992	9.448727	13.414023
Ce	4.057396	9.206346	11.004670
Ce	11.906163	8.663794	6.885574
Ce	8.504617	11.039648	13.371416
Ce	8.445741	10.075516	6.575716
O	14.089148	13.677230	8.282139
O	6.796785	6.573316	9.064330
O	13.048296	15.970151	9.025006
O	9.516478	12.917469	13.397144
O	4.566142	11.153454	10.355769
O	11.038581	7.186688	13.736559
O	7.689700	8.753007	12.983189
O	12.742451	6.095330	10.529115
O	7.499551	7.985684	6.638969
O	9.192087	12.200006	6.192085
O	12.937950	10.724285	6.357346
O	10.968419	6.585556	6.850085
O	14.088656	9.317735	12.413619
O	6.962038	7.676151	15.369279
O	13.087145	12.480897	10.577848
O	12.027134	9.905540	8.962358
O	10.314257	12.415129	10.126140
O	12.847643	11.654004	13.233995
O	9.785644	8.874160	7.911536
O	11.603992	8.480550	11.350909
O	9.244660	7.487220	10.246789
O	6.190214	9.026106	10.010047
O	15.436585	13.407723	11.566896
O	7.268326	13.318460	8.984873
O	12.405092	14.761515	11.853030
O	6.595141	10.829108	14.373919
O	3.935782	9.724330	13.076460
O	14.827660	10.505613	9.849854
O	12.208341	13.919399	5.287566
O	8.040091	10.798401	11.135833
O	3.813123	8.203026	9.037380

O	10.518416	10.702890	12.196233
O	10.409080	9.908992	5.457436
O	8.642815	11.039927	8.533913
O	9.810627	9.552284	14.604533
O	13.496735	7.717105	7.773141
O	10.308708	15.113655	7.768160
O	9.757972	4.253216	8.632411
O	11.569600	12.745978	7.600160
O	5.301098	7.627702	12.217337
O	6.314230	10.238402	7.495300
O	8.641254	6.220967	12.641816
O	11.399683	3.537821	10.149910
Ag	12.219726	5.884312	8.479201
C	10.803108	4.130893	9.266505

65

Ag1aV->**Ag2aV** E = -505.68 eV

Ce	13.798070	8.368152	9.834753
Ce	12.300627	14.947150	7.346177
Ce	13.961459	14.556295	10.458710
Ce	10.544574	6.169280	11.393255
Ce	5.631461	8.097996	7.964289
Ce	8.943539	7.313298	14.385133
Ce	9.236790	13.290628	8.304868
Ce	9.913198	10.075103	9.929455
Ce	7.168027	7.233368	11.143045
Ce	5.699621	8.773965	14.132844
Ce	11.250858	12.112043	5.462435
Ce	6.481675	11.334226	9.529869
Ce	11.368265	12.685487	12.096233
Ce	9.020709	6.754793	7.979425
Ce	14.704144	11.315247	11.751297
Ce	13.392414	11.618629	8.357818
Ce	12.104462	9.063823	13.185169
Ce	4.051520	9.109308	10.998924
Ce	11.750267	8.742744	6.723450
Ce	8.595961	10.750315	13.318577
Ce	8.287927	10.099385	6.398906
O	14.052352	13.695873	8.252330
O	6.956288	6.501114	9.012429
O	12.998985	15.943568	9.119023
O	9.665523	12.583611	13.406994
O	4.520440	11.076262	10.411956
O	10.957519	6.866447	13.510478
O	7.709239	8.540413	12.951982
O	7.446101	7.830818	6.687225
O	9.205377	12.379069	6.140542
O	12.886474	10.735164	6.319375
O	10.684327	6.723862	6.588900
O	14.236778	9.200146	12.056587

O	7.043244	7.435145	15.307891
O	13.124049	12.372758	10.501738
O	12.106044	9.789490	8.901900
O	10.292488	12.340389	10.110676
O	12.961339	11.403468	13.107350
O	9.786822	8.853013	7.855879
O	12.000675	7.714203	11.121280
O	9.224912	7.686328	10.263461
O	6.160704	8.940190	10.000421
O	15.444903	13.299183	11.515336
O	7.206141	13.296155	9.055106
O	12.413493	14.567139	11.893233
O	6.704189	10.594600	14.392537
O	3.992144	9.577607	13.110276
O	14.839032	10.439503	9.594795
O	12.132181	14.039422	5.327966
O	8.021299	10.709817	11.120063
O	3.792001	8.112014	9.037921
O	10.629942	10.319020	11.996522
O	10.459616	9.975664	5.339753
O	8.549324	11.061804	8.478683
O	9.898711	9.251790	14.504911
O	13.429873	7.615014	7.621252
O	10.250701	15.135354	7.859104
O	10.171224	5.269654	9.371061
O	11.524571	12.754086	7.610511
O	5.277347	7.481428	12.179679
O	6.101111	10.175053	7.524552
O	8.606185	6.002663	12.439406
O	12.181791	3.877580	10.231242
Ag	12.517216	5.704581	7.793867
C	11.946031	4.281239	9.156289

65

Ag2aV->**Ag3aV** E = -504.85 eV

Ce	13.615452	8.195168	10.396017
Ce	12.526972	14.718524	7.412943
Ce	14.075240	14.305524	10.554694
Ce	10.344279	6.288440	11.569347
Ce	5.493195	8.246307	7.946856
Ce	8.554153	7.465577	14.435393
Ce	9.350816	13.233110	8.385032
Ce	9.870633	9.931534	10.073464
Ce	6.888373	7.329264	11.152145
Ce	5.389822	9.024004	14.060980
Ce	11.303583	12.008002	5.506405
Ce	6.487130	11.451001	9.536916
Ce	11.245084	12.712142	12.168396
Ce	8.815578	6.739886	8.003386
Ce	14.561281	11.151032	12.048719

Ce	13.399879	11.313308	8.511680
Ce	11.728184	9.179100	13.464221
Ce	3.860956	9.374272	10.900371
Ce	11.584902	8.683384	6.659624
Ce	8.365178	10.956649	13.335742
Ce	8.231903	10.148531	6.490734
O	14.180346	13.315716	8.378374
O	6.632664	6.578826	9.051667
O	13.365748	15.732846	9.107437
O	9.492685	12.791003	13.402939
O	4.466821	11.298983	10.341545
O	10.655460	6.982962	13.657117
O	7.436191	8.700265	12.946316
O	13.973058	5.521828	9.335986
O	7.332793	7.935447	6.701597
O	9.284599	12.341688	6.186091
O	12.883378	10.506985	6.355671
O	10.858591	6.658644	6.542428
O	13.960887	9.011351	12.448609
O	6.641098	7.633853	15.297239
O	13.107293	12.153281	10.640121
O	12.292491	9.335168	8.797092
O	10.351766	12.342763	10.164822
O	12.806513	11.394641	13.306515
O	9.743693	8.827565	7.811588
O	11.515417	8.143845	11.292188
O	8.980940	7.594658	10.254138
O	6.006367	9.085827	9.979142
O	15.458124	13.034921	11.730372
O	7.326799	13.389597	9.055761
O	12.495524	14.497751	11.895258
O	6.424295	10.820805	14.332910
O	3.721103	9.855574	13.005216
O	14.742905	10.114785	9.958197
O	12.288766	13.861976	5.367870
O	7.874874	10.841032	11.181124
O	3.609557	8.381339	8.930629
O	10.414932	10.500598	12.241738
O	10.383544	9.903311	5.322365
O	8.542219	11.050196	8.524877
O	9.558921	9.378875	14.622066
O	10.535347	15.026586	7.941762
O	10.842218	5.206575	9.761474
O	11.635687	12.539511	7.661936
O	4.982293	7.710039	12.136134
O	6.086775	10.317730	7.495638
O	8.301145	6.117655	12.510343
O	12.906678	5.476525	11.360400
Ag	11.996828	5.485167	7.865458
C	13.071015	5.311665	10.163878

65

TS **1a** -> **2a** E = -508.944

Ce	13.526723	8.298769	10.058153
Ce	12.381065	14.925836	7.345813
Ce	14.024010	14.556223	10.480434
Ce	10.830953	6.235059	11.224421
Ce	5.729121	8.111790	7.982533
Ce	9.082839	7.375766	14.296509
Ce	9.292256	13.338667	8.338537
Ce	9.985259	10.068676	10.032929
Ce	7.290405	7.171923	11.136705
Ce	5.796083	8.716790	14.122626
Ce	11.320391	12.071780	5.513182
Ce	6.532456	11.384366	9.542850
Ce	11.281471	12.894162	12.134813
Ce	9.080475	6.726715	7.960218
Ce	14.618037	11.328378	11.841528
Ce	13.443073	11.580062	8.417162
Ce	11.915085	9.298755	13.206067
Ce	4.162071	9.088178	11.007230
Ce	11.838982	8.675996	6.823776
Ce	8.527950	10.941319	13.348010
Ce	8.432462	10.107100	6.563903
O	14.126997	13.644080	8.303245
O	6.935638	6.526025	8.993025
O	13.084798	15.916960	9.116940
O	9.545584	12.831507	13.414812
O	4.578506	11.068393	10.397419
O	11.157554	7.143578	13.446729
O	7.803068	8.608330	12.909858
O	12.843530	6.280041	10.700802
O	7.569884	7.994296	6.671286
O	9.217561	12.283657	6.200217
O	12.938012	10.738366	6.357163
O	10.809243	6.845302	6.660907
O	13.943727	9.163475	12.210772
O	7.194315	7.423247	15.258480
O	13.114189	12.411987	10.569866
O	12.055293	9.929616	8.963038
O	10.298028	12.499694	10.163237
O	12.868410	11.457493	13.195567
O	9.827082	8.942838	7.923418
O	11.511022	8.484893	11.121066
O	9.264380	7.610692	10.178877
O	6.273233	8.955077	10.007484
O	15.461588	13.244731	11.572237
O	7.248632	13.342688	9.017643
O	12.486230	14.675167	11.923517
O	6.712099	10.596290	14.449041

O	4.096632	9.542235	13.107879
O	14.738560	10.322040	9.822773
O	12.209405	13.983467	5.333543
O	8.009238	10.854865	11.167381
O	3.888482	8.128710	9.032257
O	10.533918	10.654392	12.140424
O	10.410101	9.974190	5.452502
O	8.632123	11.095059	8.539426
O	9.991857	9.411084	14.472685
O	13.466312	7.852532	7.996477
O	10.336037	15.154144	7.817131
O	9.987587	4.943923	9.211186
O	11.587862	12.754595	7.641159
O	5.398818	7.448483	12.177873
O	6.305403	10.227491	7.497104
O	8.825417	6.073501	12.456094
O	9.275139	3.230709	7.411950
C	9.261877	4.348577	7.788119

65
TS **2a** -> **3a** E = 511.123

Ce	13.678696	8.446881	9.923084
Ce	12.335992	14.936755	7.288824
Ce	13.994930	14.579244	10.425456
Ce	10.347159	6.358828	11.488894
Ce	5.584508	8.199196	8.043607
Ce	8.884862	7.475588	14.495554
Ce	9.264698	13.328959	8.278365
Ce	9.867940	10.134581	9.980252
Ce	7.059369	7.358624	11.228678
Ce	5.669564	8.981573	14.190524
Ce	11.278528	12.036554	5.473056
Ce	6.471205	11.453714	9.537698
Ce	11.381161	12.756245	12.081293
Ce	8.982673	6.753696	8.089843
Ce	14.711198	11.349095	11.769040
Ce	13.388145	11.625514	8.355569
Ce	12.042896	9.144060	13.253970
Ce	4.009098	9.289349	11.058089
Ce	11.721767	8.648499	6.854488
Ce	8.585534	10.896606	13.347313
Ce	8.294563	10.105166	6.461568
O	14.086767	13.686404	8.232668
O	6.847459	6.607253	9.115742
O	13.016704	15.930746	9.084924
O	9.702196	12.716577	13.404641
O	4.503849	11.231939	10.409019
O	10.887786	7.013128	13.602810
O	7.674403	8.698832	13.013527

O	10.632657	6.792358	6.715919
O	7.399747	7.892073	6.766210
O	9.232203	12.367586	6.136607
O	12.895899	10.681334	6.392530
O	14.175491	9.175007	12.016793
O	6.986789	7.650446	15.402844
O	13.159037	12.403053	10.506222
O	12.076256	9.847766	9.029749
O	10.307996	12.409203	10.117646
O	12.998954	11.438563	13.115013
O	9.756224	8.903915	7.943922
O	11.952270	7.820826	11.159619
O	9.108561	7.847525	10.337853
O	6.120799	9.077474	10.072225
O	15.483102	13.296593	11.502426
O	7.236556	13.395045	9.012605
O	12.472001	14.615394	11.876076
O	6.706472	10.789100	14.429486
O	3.984774	9.817319	13.155959
O	14.798553	10.375942	9.644586
O	12.194530	13.961040	5.306021
O	8.003944	10.854852	11.156018
O	3.735837	8.248816	9.131660
O	10.613580	10.411103	12.051475
O	10.462763	9.937286	5.415195
O	8.533417	11.121976	8.495967
O	9.886795	9.399105	14.554965
O	13.406297	7.711791	8.001117
O	10.294967	15.159190	7.779990
O	9.997082	5.365856	9.560239
O	11.546930	12.753754	7.611101
O	5.212340	7.667410	12.274165
O	6.092234	10.262564	7.561700
O	8.461836	6.145244	12.599807
O	11.830577	4.377441	11.373320
O	12.650646	4.255918	9.192234
C	12.051944	4.392439	10.185870

64

TS **1aV** -> **2aV** E = 502.611

Ce	13.684664	8.427142	9.864150
Ce	12.323505	14.951991	7.284982
Ce	13.990300	14.566233	10.415857
Ce	10.402299	6.199086	11.322476
Ce	5.538775	8.243642	8.017494
Ce	8.872647	7.387660	14.359828
Ce	9.248451	13.348716	8.276713
Ce	9.850777	10.131189	9.940022
Ce	7.038671	7.342115	11.194956

Ce	5.665386	8.941314	14.179956
Ce	11.233617	12.082911	5.444614
Ce	6.457864	11.473931	9.538888
Ce	11.377515	12.732999	12.071161
Ce	8.871844	6.788479	8.057906
Ce	14.694812	11.323215	11.736752
Ce	13.375700	11.632941	8.330933
Ce	12.014002	9.060783	13.139774
Ce	3.999050	9.319191	11.056370
Ce	11.735274	8.680205	6.779074
Ce	8.592969	10.854331	13.333042
Ce	8.267931	10.138067	6.435080
O	14.076812	13.686296	8.214981
O	6.786497	6.619467	9.091046
O	13.033513	15.935214	9.073062
O	9.711660	12.675123	13.405364
O	4.495458	11.269982	10.411061
O	10.879180	6.930482	13.429191
O	7.642024	8.671967	12.971457
O	10.593303	6.598700	6.422537
O	7.349275	7.938554	6.748116
O	9.195083	12.407892	6.133783
O	12.877955	10.703207	6.339899
O	14.148757	9.133681	11.962602
O	6.990864	7.545694	15.316416
O	13.146772	12.392149	10.484395
O	12.078846	9.846237	8.978364
O	10.298305	12.415422	10.107749
O	12.984729	11.377952	13.077501
O	9.772921	8.886420	7.861773
O	11.956390	7.759856	11.060308
O	9.103271	7.844627	10.333804
O	6.098319	9.099845	10.053760
O	15.472100	13.272121	11.479510
O	7.227021	13.418911	9.017842
O	12.467339	14.596798	11.866751
O	6.744214	10.729592	14.456252
O	4.010702	9.845132	13.155252
O	14.794500	10.375865	9.602629
O	12.160453	13.997252	5.292715
O	7.997624	10.877202	11.151770
O	3.706127	8.303874	9.114777
O	10.614246	10.386856	11.999230
O	10.417855	10.002601	5.354980
O	8.518122	11.134968	8.478827
O	9.910116	9.302468	14.469616
O	13.419793	7.688302	7.937173
O	10.290289	15.179859	7.785684
O	9.997020	5.410308	9.465855
O	11.527368	12.766187	7.594987

O	5.163220	7.667249	12.249012
O	6.076835	10.298634	7.546458
O	8.458805	6.090459	12.461326
O	9.473101	4.213497	5.633902
C	9.329848	5.261524	6.094336

64

TS **2aV** –> **3aV** E = 503.547

Ce	13.470224	8.204910	10.607221
Ce	12.465463	14.688895	7.266141
Ce	14.032937	14.464985	10.401975
Ce	10.707266	6.454759	11.929948
Ce	5.577882	8.124390	8.132564
Ce	8.765908	7.684026	14.637778
Ce	9.336958	13.197019	8.361360
Ce	9.959642	9.982038	10.214908
Ce	7.129599	7.339600	11.356761
Ce	5.565149	9.096261	14.197587
Ce	11.291506	11.863977	5.507228
Ce	6.530274	11.407068	9.615893
Ce	11.257490	12.900603	12.145072
Ce	8.887261	6.723257	8.050593
Ce	14.601918	11.339767	11.985605
Ce	13.389690	11.334010	8.541383
Ce	11.842539	9.510840	13.675320
Ce	3.992183	9.279835	11.066473
Ce	11.628358	8.571153	6.767277
Ce	8.457494	11.130897	13.406401
Ce	8.303448	10.050738	6.662536
O	14.117649	13.375427	8.283078
O	6.764412	6.529429	9.187500
O	13.244050	15.813645	8.889011
O	9.503571	13.021386	13.364931
O	4.509289	11.199480	10.396330
O	10.892733	7.267772	13.997664
O	7.604705	8.808886	13.072488
O	12.634798	6.223072	10.604900
O	7.421689	7.899638	6.775478
O	9.199363	12.131270	6.195985
O	12.876616	10.416696	6.401320
O	14.044492	9.210303	12.506223
O	6.808528	7.830887	15.489047
O	13.118101	12.297902	10.590365
O	12.289015	9.334363	8.927161
O	10.364954	12.418159	10.134736
O	12.918854	11.684675	13.297530
O	9.748083	8.806318	7.934750
O	11.562700	8.510107	11.554699
O	9.201239	7.423835	10.331275
O	6.132754	9.019601	10.090463

O	15.452318	13.276815	11.588676
O	7.304803	13.334975	9.032612
O	12.459253	14.715474	11.733334
O	6.502893	10.950272	14.401975
O	3.825727	9.830716	13.115150
O	14.717147	10.191073	9.972377
O	12.235084	13.699453	5.248498
O	7.928370	10.889490	11.233678
O	3.720785	8.227062	9.106482
O	10.472772	10.748398	12.352104
O	10.327866	9.782478	5.444338
O	8.578987	11.001587	8.566480
O	9.663569	9.652268	14.762020
O	10.771662	6.721870	6.575919
O	10.451114	14.980276	7.740558
O	10.934824	3.971844	10.621592
O	11.604303	12.502406	7.605324
O	5.203627	7.701681	12.305684
O	6.218699	10.205483	7.547776
O	8.483083	6.266988	12.824054
O	11.253431	4.818297	8.458871
C	11.204247	4.462692	9.620002
